# Discovery of novel reversible inhibitor of DprE1 based on benzomorpholine for the treatment of tuberculosis

**DOI:** 10.1128/spectrum.04721-22

**Published:** 2023-09-12

**Authors:** Wang Xiang, Hualong He, Xianjie Duan, Zhiqun He, Xinyue Xu, Mengya Liao, Fei Teng, Xiao Li, Tianwen Luo, Jumei Zeng, Luoting Yu, Chao Gao

**Affiliations:** 1 State Key Laboratory of Biotherapy/Collaborative Innovation Center for Biotherapy, West China Hospital, West China Medical School, Sichuan University, Chengdu, Sichuan, China; 2 West China School of Public Health and West China Fourth Hospital, Sichuan University, Chengdu, Sichuan, China; 3 Center of Gerontology and Geriatrics, West China Hospital, Sichuan University, Chengdu, Sichuan, China; 4 Center of Infectious Diseases and Laboratory of Human Diseases and Immunotherapies and Institute of Immunology and Inflammation, Frontiers Science Center for Disease-related Molecular Network, West China Hospital, Sichuan University, Chengdu, Sichuan, China; Indian Institute of Science Bangalore, Bangalore, Karnataka, India

**Keywords:** tuberculosis, DprE1, benzomorpholine, antimycobacterial agents

## Abstract

About a quarter of the world’s population is infected with *Mycobacterium tuberculosis*, equivalent to about two billion people. With the emergence of multidrug-resistant tuberculosis, those existing anti-tuberculosis drugs no longer meet the demand for cure anymore; there is an urgent need for the development of new anti-tuberculosis drugs. Decaprenylphosphoryl-β-D-ribose 2´-epimerase (DprE1) has been proven to be a potential antimycobacterial target, and several inhibitors have entered clinical trial. Herein, we designed and synthesized a series of compounds based on the indole and benzomorpholine by using the strategy of scaffold hopping. The preferred compound **B18** showed strong antimycobacterial activity in H37Rv and drug-resistant clinical isolates. In addition, compound **B18** did not exhibit antimycobacterial efficacy against other species of strains. Subsequently, the target of **B18** was identified as DprE1 by analyzing spontaneous compound-resistant mutation data, and a docking study was performed to illustrate the binding mode between **B18** and DprE1. In general, compound **B18** is compatible to current DprE1 inhibitors, even higher phosphodiesterase 6C selectivity and plasma protein binding rate, which represent a new type of effective reversible DprE1 inhibitor.

**IMPORTANCE:**

Drug therapy remains the cornerstone of tuberculosis (TB) treatment, yet first-line anti-tuberculosis drugs are associated with significant adverse effects that can compromise patient outcomes. Moreover, prolonged and widespread use has led to an alarming rise in drug-resistant strains of *Mycobacterium tuberculosis*, including multidrug-resistant [MDR-tuberculosis (TB)] and extensively drug-resistant (XDR-TB) forms. Urgent action is needed to develop novel anti-tuberculosis agents capable of overcoming these challenges. We report that compound **B18**, a decaprenylphosphoryl-β-D-ribose 2´-epimerase inhibitor with a benzomorpholine backbone, exhibits potent activity against not only the non-pathogenic strain H37Ra, but also the pathogenic strain H37Rv and clinical MDR and XDR strains. Preliminary druggability studies indicate that **B18** possesses high safety and acceptable pharmacokinetic properties, rendering it a promising candidate for further development as a novel anti-tuberculosis agent.

## INTRODUCTION

Tuberculosis (TB), a chronic disease caused by *Mycobacterium tuberculosis* (*Mtb*) infection, is one of the top 10 causes of death worldwide and the leading cause of death from a single infectious agent (1). While the global incidence rate of TB was reduced by 11% from 2015 to 2020 as a result of the worldwide TB prevention and control efforts (1). However, this number falls significantly behind the 2020 milestone of a 20% reduction in the TB incidence rate set by the World Health Organization (2).

The approved first-line drugs that form the core of current standard TB treatment regimens for drug-susceptible TB, including rifampicin and isoniazid—the two most effective first-line drugs, were all developed in the 1950s and 1960s. The prolonged and widespread use of these drugs over the last 30 years led to a rapid growth of drug-resistant TB worldwide (3). A global total of 206,030 people with multidrug- or rifampicin-resistant TB (MDR/RR-TB) were detected and notified in 2019, a 10% increase from 186,883 in 2018 (2). The main reason leading to the rise of drug-resistant TB has been attributed to the practical or logistical difficulties that hinder patient adherence to medication regimens (4). Due to the low efficacy and high toxicity of all currently available anti-TB agents, treatment regimens are always lengthy and complicated, and cause a significant health resource burden, especially with treatment for drug-resistant TB (3). The premature treatment discontinuation among a fraction of patients is almost inevitable. It is urgently needed to develop new classes of anti-TB drugs with novel targets that are safer, superior in terms of efficacy, and less prone to resistance.

Decaprenylphosphoryl-β-D-ribose 2´-epimerase (DprE1), an essential enzyme for the biosynthesis of the cell wall of mycobacteria, has been proven to be a potential anti-TB drug target in recent years (5, 6). Several DprE1 inhibitors are currently under evaluation in clinical trials (6 – 9). However, the slow clinical progress of covalent inhibitors BTZ-043 and PBTZ-169 may be attributed to potential safety risks and unsatisfactory pharmacokinetic properties (10). The synthetic challenge posed by reversible inhibitor OPC-167832 may result in a high drug price (9). TBA-7371 (8) is a reversible inhibitor of DprE1 currently in a phase II trial, which shows a high antimycobacterial effect *in vitro*, but its off-target activity against phosphodiesterase 6 (PDE6) presents potential safety issues. There are ongoing efforts seeking better TBA-7371 analogs. Several design examples based on TBA-7371 demonstrated either maintained or improved antibacterial activity (Fig. S1), which provided us with the confidence to further explore potential anti-TB drugs (11 – 13).

In the present study, we have designed and synthesized a series of compounds derived from TBA-7371, in which the azaindole core of TBA-7371 has been replaced by a benzomorpholine core utilizing a scaffold hopping strategy. We evaluated the *in vitro* potency of the new series in susceptibility testing against both drug-susceptible *Mtb* strains and drug-resistant *Mtb* clinical isolates, and identified compound **B18** as the most active compound in the series that demonstrated a promising novel class of reversible DprE1 inhibitor with potent antimycobacterial activity.

## RESULTS AND DISCUSSION

### Rational design of DprE1 inhibitor

**Fig 1:**
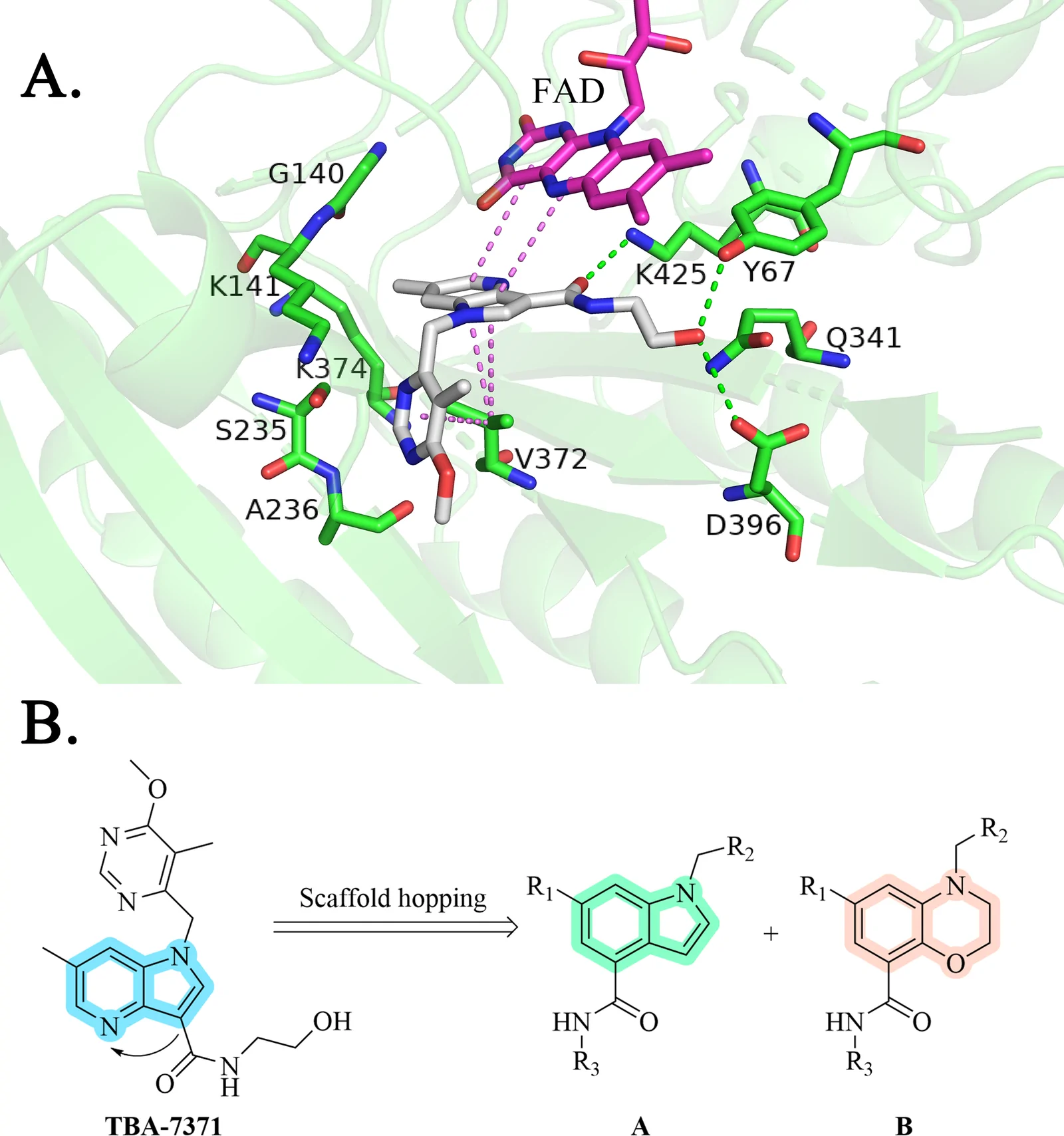
(**A**) Predicted binding model of TBA-7371 in complex with DprE1. TBA-7371 is depicted by gray solid line, FAD is depicted by purple solid line, residues of DprE1 are depicted by green solid line, hydrogen bonds are depicted by green dashed lines, alkyl-pi interactions are depicted by purple dashed lines. (**B**) Design strategy of target compounds.

The docking study of TBA-7371 with DprE1 suggested that the replacement of the core may be tolerated because 4-azaindole binds to a larger substrate binding cavity (Fig. 1A). As a common skeleton in medicinal chemistry, indole has the advantages of high biological activity and low chemical synthesis difficulty, so it was introduced in our investigation. Furthermore, indole is an aromatic ring with higher electronic density than 4-azaindole, which may be helpful to maintain π-π stacking interaction between 4-azaindole and flavin adenine dinucleotide (FAD) or DprE1. In addition, we previously reported the discovery of some potent Enhancer of Zeste Homolog 2 (EZH2) inhibitors based on a benzomorpholine core that exhibited favorable physicochemical properties and relatively good metabolic stability (14). In the present study, we attempted to derive a new class of DprE1 inhibitors from TBA-7371 using a scaffold hopping approach that replaces the central azaindole core of TBA-7371 with a benzomorpholine ring structure. The pyrrole ring of TBA-7371 is closer to the solvent region, and the replaced morpholine ring has a higher hydrophilic character and may be a suitable choice. Considering the difficulty of synthesis, we placed the attachment site of the amide side chain on the benzene ring of indole and benzomorpholine, and a similar transposition was successful in the design of DprE1 inhibitors based on benzimidazole. These designs were aimed at introducing some desirable changes in the physicochemical profile of the compound with novel core that in turn could potentially improve their biological activities.

### Synthesis of compounds

**Fig 2:**
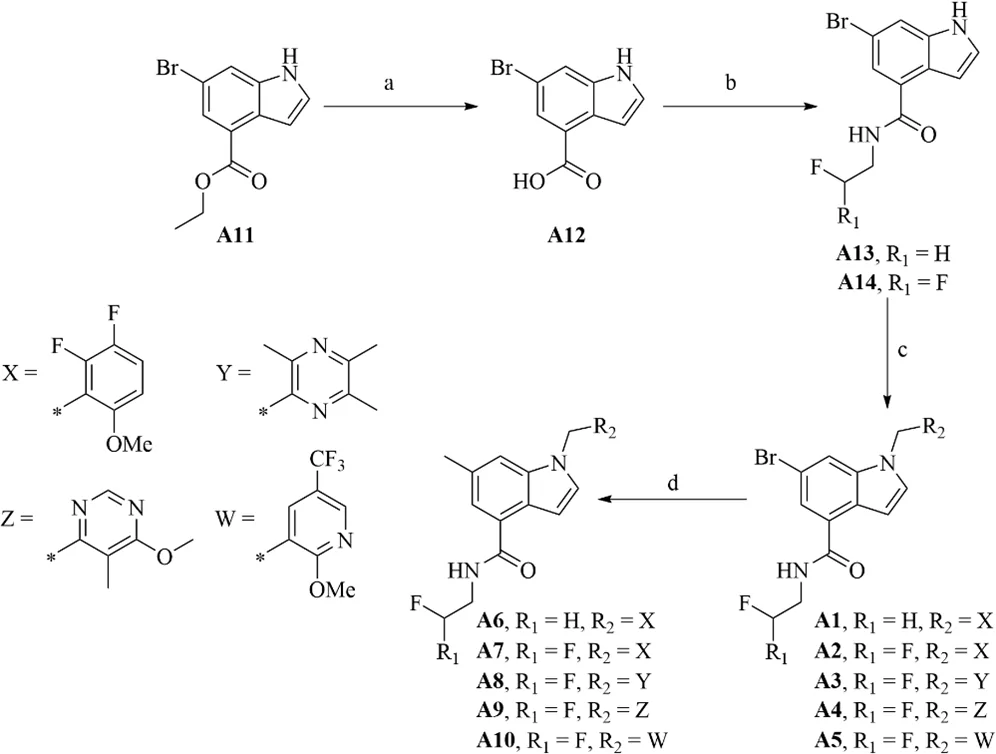
Synthesis of compounds A1–10. Reaction conditions: (a) NaOH, MeOH, H_2_O, 40°C, 4 h; (b) N-(3-Dimethylaminopropyl)-N′-ethylcarbodiimide hydrochloride (EDCI), N-Hydroxy-7-azobenzotriazole (HOAt), 4-Methylmorpholine (NMM), Dimethyl sulfoxide (DMSO), room temperature (rt), overnight; (c) ArCH_2_Br, K_2_CO_3_, *N*,*N*-Dimethylformamide (DMF), rt, 4 h; (d) CH_3_BF_3_K, Pd(dppf)Cl_2_, K_2_CO_3_, 1,4-dioxane, H_2_O, 105°C, N_2_, 5 h.

The synthesis of final compounds **A1–10** was shown in Fig. 2. The commercially available **A11** was hydrolyzed to obtain the intermediate **A12**, and then **A1–5** was obtained by amide condensation and nucleophilic substitution reaction. Subsequently, the palladium-catalyzed coupling reaction of **A1–5** afforded **A6–10**, respectively.

**Fig 3:**
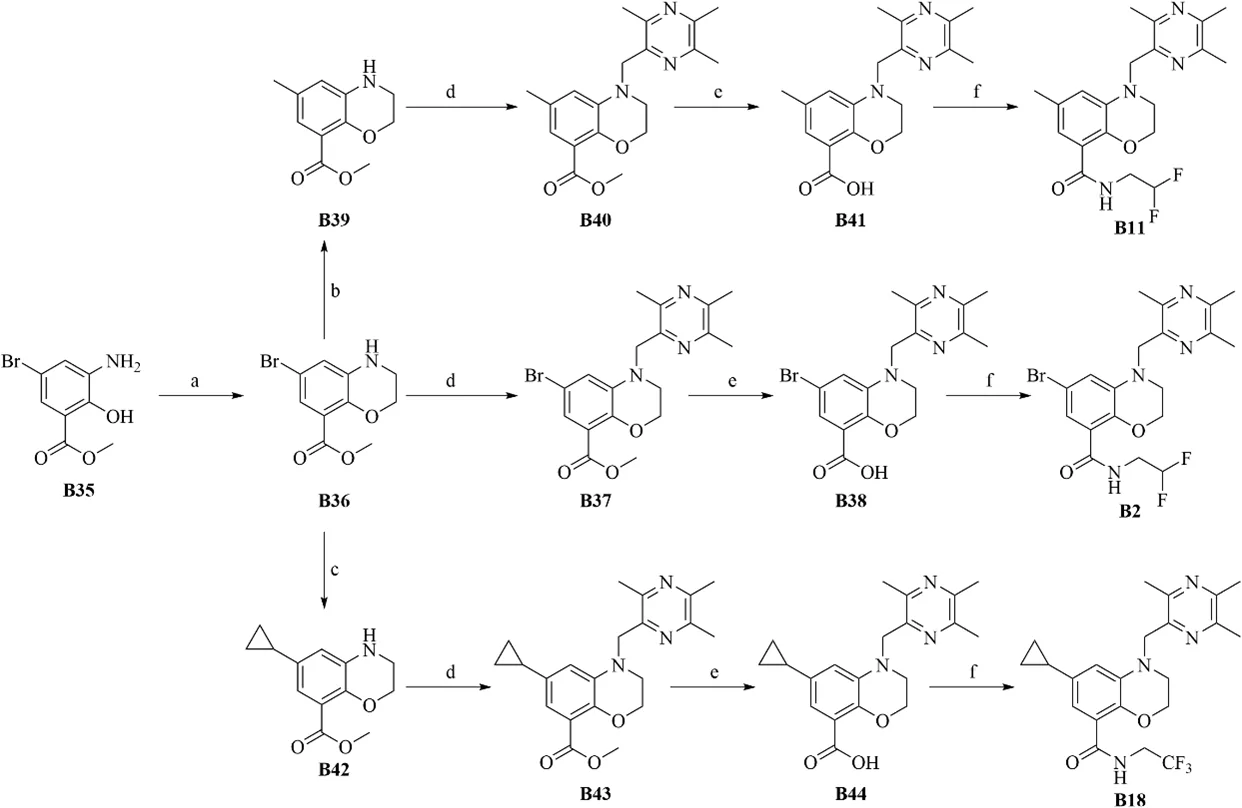
Synthesis of compounds **B2**, **B11,** and** B18**. Reaction conditions: (a) 1,2-dibromoethane, K_2_CO_3_, DMF, 80°C, 8 h, 95.6%; (b) CH_3_BF_3_K, Pd(dppf)_2_Cl_2_, K_2_CO_3_, 1,4-dioxane, H_2_O, N_2_, 110°C, 1 day，57%; (c) cyclopropylboronic acid, Pd(OAc)_2_, (C_6_H_11_)_3_P, K_3_PO_4_, PhMe, H_2_O, 100°C, overnight, 64%; (d) 2-(bromomethyl)-3,5,6-trimethylpyrazine, Na_2_CO_3_, DMF, 80°C, 3 h, 62%; (e) NaOH, MeOH:H_2_O:THF = 1:1:1, 1 h, 70°C; (f) 2,2-difluoroethylamine hydrochloride or 2,2,2-trifluoroethylamine hydrochloride, 2-(7-Azabenzotriazol-1-yl)-N,N,N',N'-tetramethyluronium hexafluorophosphate (HATU), N,N-Diisopropylethylamine (DIPEA), DMF, dichloromethane (DCM), rt, 2 h，60%–90%.

The synthesis of final compounds **B2**, **B11**, and **B18** was outlined in Fig. 3. Nucleophilic Aromatic Substitution reaction (SNAr) displacement reaction of commercially available **B35** with 1,2-dibromoethane afforded crucial intermediate **B36**. Undergoing a palladium-catalyzed coupling reaction, **B36** was transformed to **B39** and **B42**, which subsequently undergoes displacement, deprotection and an amide condensation reaction to afford final compounds **B2**, **B11**, and **B18** with an excellent overall yield.

**Fig 4:**
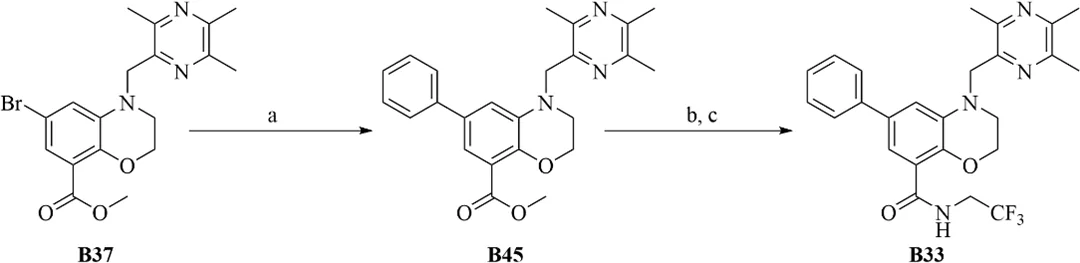
5Synthesis of compound **B33**. Reaction conditions: (a) Phenylboronic acid, Pd(dppf)_2_Cl_2_, Na_2_CO_3_, 1,4-dioxane, H_2_O, N_2_, 100°C, 6 h, 68%; (b) NaOH, MeOH:H_2_O:THF = 1:1:1, 1 h, 70°C; (c) 2,2,2-trifluoroethylamine hydrochloride, HATU, DIPEA, DMF, rt, 2 h, 83%.

The synthesis of compound **B33** was outlined in Fig. 4. Compound **B45** was obtained by a Suzuki coupling reaction between compound **B37** and phenylboronic acid, and followed by a two-step reaction of hydrolysis and amide condensation to afford the compound **B33**.

### Structure−activity relationship

**Fig 5:**
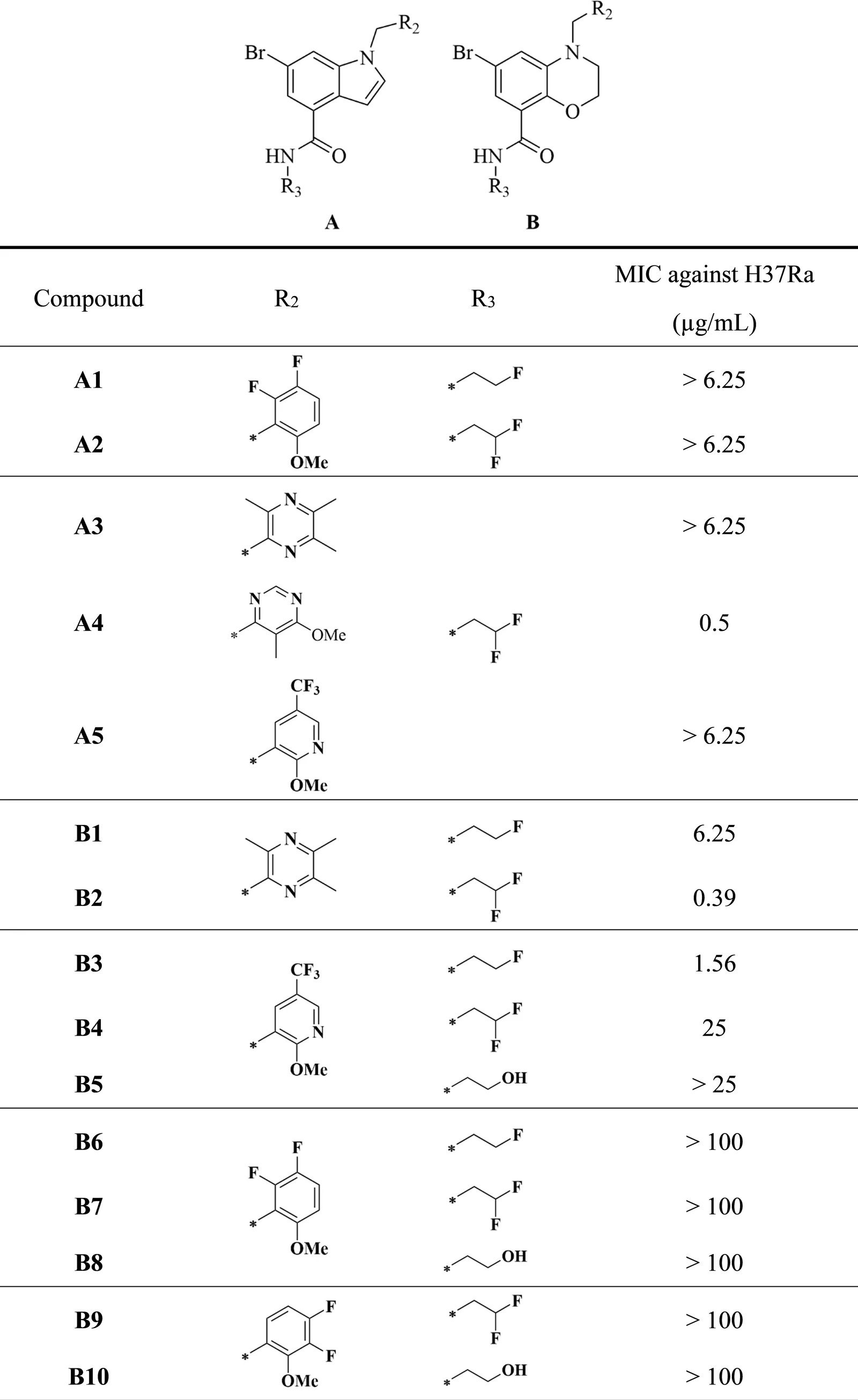
The antimycobacterial activity of compounds A1–5 and B1–10 against H37Ra.

We first kept R_1_ as the bromine atom while investigating the effects of different substituents at R_2_ and R_3_ (Fig. 5) on antimycobacterial activity by *in vitro* susceptibility testing against non-pathogenic drug-susceptible *Mtb* strain H37Ra. The selected substituents are either previously used in the design of TBA-7371 or are commercially available. In the indole series, most of the compounds were inactive except **A4**, which had introduced the same group as TBA-7371 at the R_2_ position and has a minimum inhibitory concentration (MIC) of 0.5 µg/mL. In benzomorpholine series, compounds **B6–10**, which were introduced either a 2,3-difluoro-6-methoxyphenyl or a 3,4-difluoro-2-methoxyphenyl at the R_2_ position, were essentially devoid of activity. Compounds **B3–5** with a 2-methoxy-5-(trifluoromethyl)pyridine group introduced at the R_2_ position exhibited moderate activity (at the µg/mL level) at least about one order of magnitude lower than TBA-7371. However, the introduction of trimethylpyrazine at the R_2_ position (Compounds **1–2,** Fig. 5) could significantly improve the antimycobacterial activity against H37Ra. **B2,** which was also introduced a 2,2-difluoroethyl group at the R_3_ position, showed a potent antimycobacterial activity (MIC = 0.39 µg/mL) approximating the level of TBA-7371. Compounds **B3–5** with a 2-methoxy-5-(trifluoromethyl)pyridine group introduced at the R_2_ position exhibited antimycobacterial activity at the microgram level, but still had an order of magnitude difference with TBA-7371.

**Fig 6:**
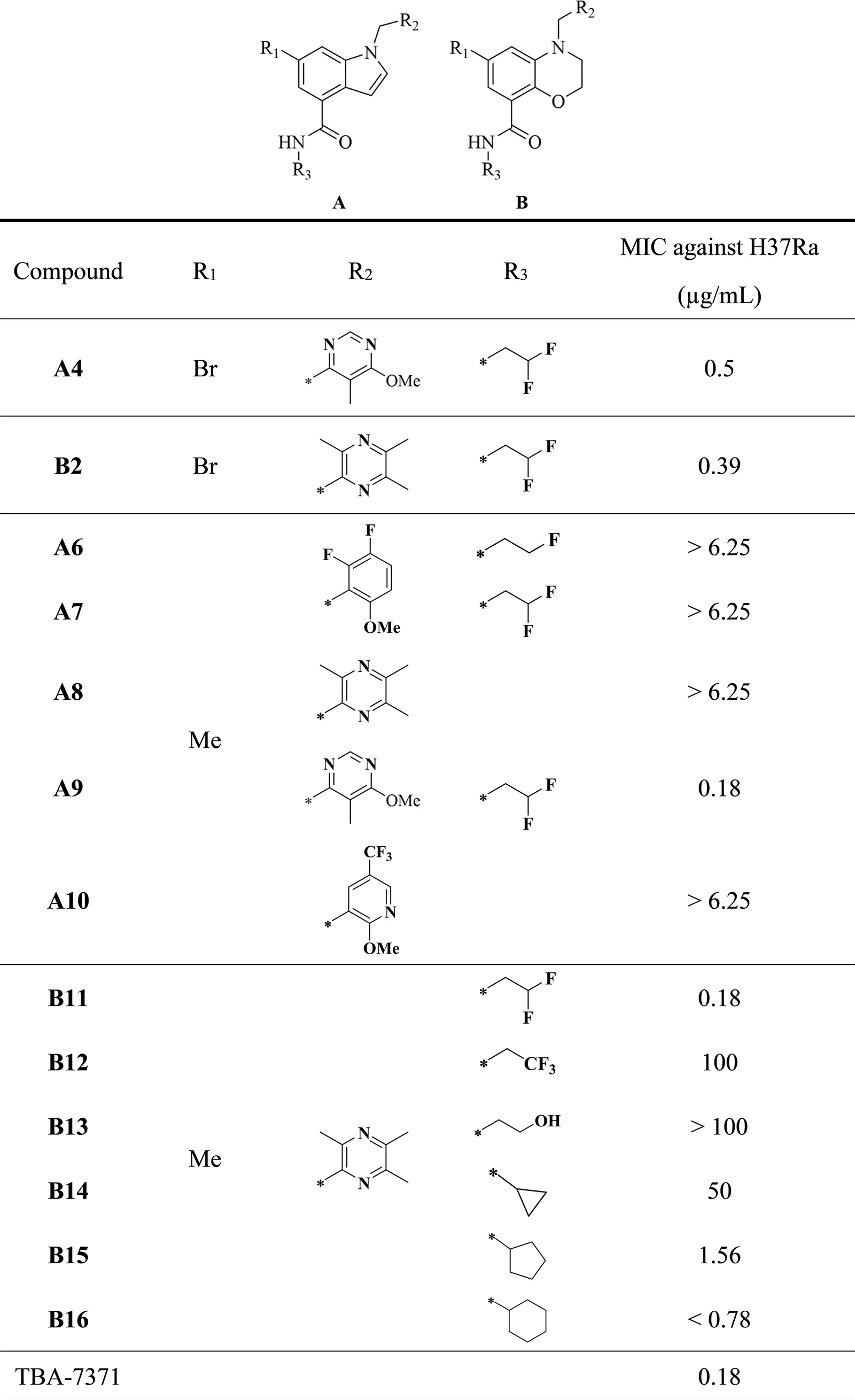
The antimycobacterial activity of compounds A6–10 and B11–16 against H37Ra.

Encouraged by its promising activity, **A4** and **B2** were chosen to serve as the bases for further synthetic optimization that explored the effects of other R_1_ substituents. We first attempted the replacement of the bromine atom with a methyl group, a preference of TBA-7371 at the R_1_ position. In the indole series, we methylated **A1–5** to afford **A6–10**, respectively. Except for **A9**, which had a twofold increase in antimycobacterial potency, the other compounds had no activity, which was consistent with the previous results. In benzomorpholine series, this substitution (**B11** vs **B2**) also resulted in a twofold increase in antimycobacterial potency. **B11** was further optimized by probing the effect of different substitutions at the R_3_ position. Replacing the 2,2-difluoroethyl group with a 2,2,2-trifluoroethyl or an ethanol group to form **B12** and **B13** (Fig. 6), however, almost abolished activity completely (MIC ≥100 µg/mL). While the cyclic alkane substitutions (**B14–16**, Fig. 6) did not improve the antimycobacterial potency as compared to **B2**, they demonstrated that bulkier substituents at the R_3_ position are more optimal.

**Fig 7:**
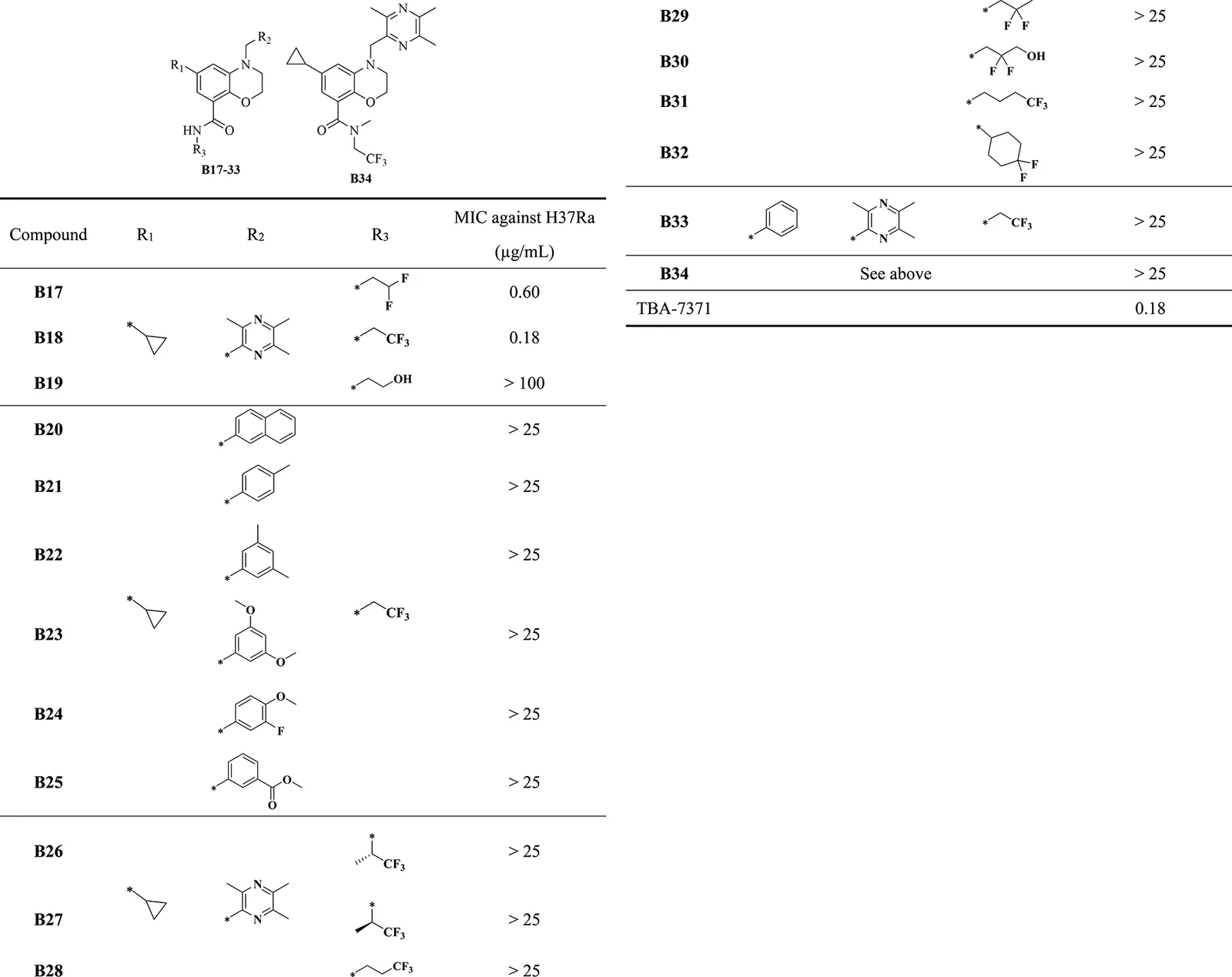
The antimycobacterial activity of compounds B17–34 against H37Ra.

We, furthermore, explored the introduction of a cyclopropyl moiety at position R_1_ in **B11–13**, which resulted in **B17–19**. In contrast to **B17** and **B19** that were found to have no improvement on the MIC against H37Ra, compound **B18** showed a very potent antimycobacterial activity against H37Ra and it has an MIC value equivalent to that of TBA-7371. Notably, compound **B18** may be bactericidal (MBC/MIC <4, Figure S2) (15). All attempts to further optimize **B18**, including the introduction of electron-rich aromatic groups at the R_2_ position (**B20-25,** Fig. 7) and modulation of linker length, methylation of -NH, or introduction of hydroxyl at the R_3_ position (**B26-34,** Fig. 7), have been unable to achieve any further improvement in antimycobacterial activity.

**Fig 8:**
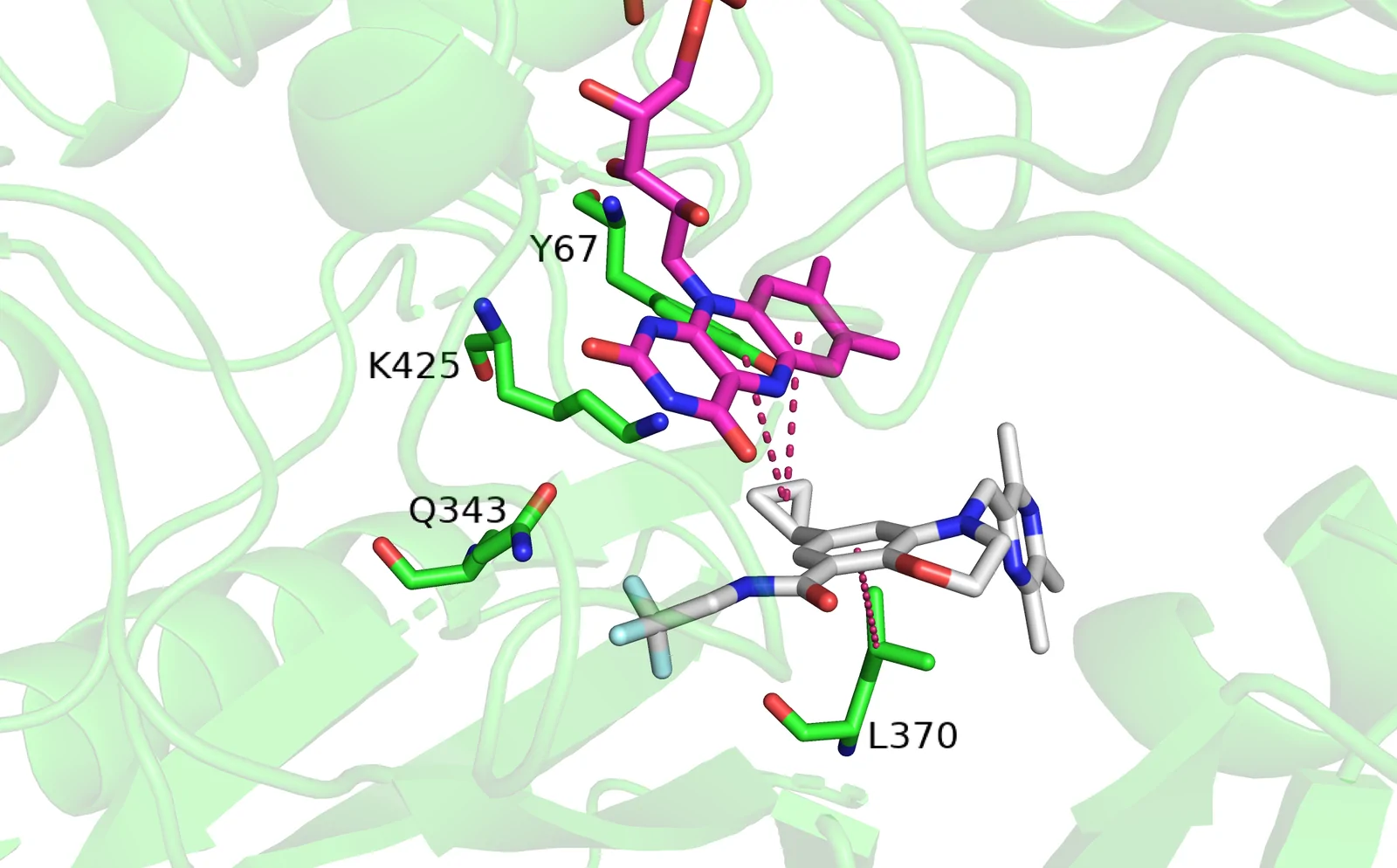
Predicted binding model of **B18** in complex with DprE1 (4F4Q). **B18** is depicted by gray solid line, FAD is depicted by purple solid line, residues of DprE1 are depicted by green solid line, alkyl-pi interactions are depicted by purple dashed lines.

### Antibacterial activity study *in vitro*

Currently, first-line anti-TB drugs have lengthy treatment cycles and poor patient adherence in the management of drug-sensitive TB. Meanwhile, second-line anti-TB drugs exhibit high toxicity and serious drug resistance when treating MDR/extensively drug-resistant (XDR)-TB patients, with a lack of strict evidence regarding their efficacy (16). Therefore, the effectiveness of new anti-TB drugs against these resistant strains is a crucial determinant for their developmental value. As the most active compounds among the whole new series of TBA-7371 derivatives, the antimycobacterial activity of **B18** was further investigated in a susceptibility testing performed on the drug-susceptible pathogenic *Mtb* strain H37Rv as well as drug-resistant clinical isolates that included MDR strains Y48 and Y17, and XDR strain Y198 (Table 1; Table S1). In addition, **B11** and **A9** were discarded due to failure in the simultaneous cultivation experiment of H37Ra resistant strains. Although **B18** has equivalent MIC to TBA-7371 against H37Ra, their activities are very different against this expanded testing panel. As shown in Table 1, **B18** was similar to TBA7371, and it showed strong potency against all the strains that we tested.

**TABLE 1:** MIC values of selected compounds against H37Rv and clinical isolates

	Strain		B18	TBA-7371	Fold increase
MIC (µg/mL)	H37Rv	3 days	0.625	2.5	4
		7 days	0.625	0.625	1
	Y48 (MDR)		0.6	0.6	1
	Y17 (MDR)		0.6	0.6	1
	Y198 (XDR)		5	1.25	0.25

### Antibacterial selectivity study

We also assessed the susceptibility of a panel of 10 representative strains of common Gram-positive and Gram-negative pathogenic bacteria toward compound **B18** (Table 2). Neither of compounds **B18** and TBA-7371 showed growth inhibitory activity on any of these strains in a treatment time of 24 h, which suggested that like TBA-7371, **B18** is basically specific antimycobacterial.

**TABLE 2:** The MIC of selected compounds against other strains

Species	Strain	24 h (µg/mL)	
		B18	TBA-7371
*Staphylococcus aureus*	ATCC29213	>64	>64
*S. aureus*	ATCC33591	>64	>64
*S. epidermidis*	ATCC12228	>64	>64
*Enterococcus faecalis*	ATCC29212	>64	>64
*E. faecium*	ATCC19434	>64	>64
*Streptococcus pneumoniae*	ATCC49619	>64	>64
*Escherichia coli*	ATCC25922	64	>64
*E. coli*	ATCC35218	>64	>64
*Klebsiella pneumoniae*	ATCC700603	64	>64
*Pseudomonas aeruginosa*	ATCC27853	64	>64

### Antibacterial mechanism study

The whole-genome sequencing (WGS) of spontaneous *Mtb* H37Ra resistant mutants, upon exposure to either **B18** or TBA-7371, was performed in order to establish cellular compound-target (DprE1 protein) engagement by the existence of DprE1 gene mutation(s) (Table 3). Spontaneous resistant mutants were isolated by growing *Mtb* H37Ra in the media containing 5×, 10×, 20× MIC of corresponding compounds. In total, 11 compound **B18**-resistant and 6 TBA-7371-resistant mutants were isolated and sequenced. All the mutants were found to possess the same point mutation at codon 314 of MRa3830 (corresponding to Rv3790 in H37Rv) that encodes the enzyme DprE1, which results in Tyr→His substitution in DprE1 that has been described previously in the resistant mutants to other DprE1 inhibitors (Table 3). These data indicated that DprE1 is a target of both **B18** and TBA-7371 and DprE1 modification confers the resistance to them.

**TABLE 3:** Sequencing of spontaneous resistant mutants identifies SNPs^*a*^ in DprE1

Compound	Mutant	Genome position of SNP	Base change	Amino-acid substitution
B18	11 resistant mutants	4236718	940 TAC→CAC	Tyr→His
TBA-7371	6 resistant mutants	4236718	940 TAC→CAC	Tyr→His

^*a*^ SNPs: single nucleotide polymorphisms.

To understand the binding mode between **B18** and DprE1, we conducted docking analysis (Fig. 8). The best binding pose showed that **B18** can bind to the receptor cavity of DprE1. Specifically, the cyclopropyl group of **B18** extends into a small pocket formed by Y67, Q341, and K425, which may be difficult to accommodate a larger group. Meanwhile, cyclopropyl also forms several important alkyl-pi interactions with FAD, which proves that the introduction of cyclopropyl is appropriate.

### Preliminary druggability study

The off-target effect of TBA-7371 on human PDE6C has been reported previously, and this potentially toxicological effect has been considered as a safety risk (6). Our fluorescence polarization assays showed the target selectivity of **B18** is significantly better than that of TBA-7371, and its IC_50_ value of PDE6C is 81 µM (Table 4). The solubility of **B18** is slightly lower than that of TBA-7371; however, **B18** has a higher plasma protein binding rate (Table 4). Moreover, cell viability assays demonstrated that neither **B18** nor TBA-7371 exhibited discernible cytotoxicity in A549 and VERO cell lines.

**TABLE 4:** Preliminary druggability study of compound B18

Compound	B18	TBA-7371
cLogP	3.8	1.21
PDE6C IC_50_/µM·L^−1^	81	5
A549 IC_50_/µM·L^−1^	>40	>40
VERO IC_50_/µM·L^−1^	>40	>40
Water solubility/µg·mL^−1^	21	54
Rat plasma protein binding rate	95.8%	33.3%

### Conclusion

Development of more effective new anti-TB regimen relies on the discovery of new classes of anti-TB agents, especially those that would target novel therapeutic targets in *Mtb*. Over the last decade, DprE1 has emerged as a promising novel antimycobacterial target, and currently, several clinical trials with DprE1 inhibitors are underway. Here, we reported the design, synthesis, and structure-activity relationship (SAR) study of a series of indole- and benzomorpholine-based novel compounds derived from TBA-7371. Scaffold hopping from the azaindole core of TBA-7371 to the benzomorpholine ring and complementing with systematic side chain optimizations led to the discovery of several lead compounds that possess potent *in vitro* antimycobacterial activity comparable or equivalent to that of TBA-7371 but have completely new structures. Particularly, the representative compound **B18,** which was identified after extensive structure optimization, showed strong activity not only against non-pathogenic strain H37Ra (MIC = 0.18 µg/mL) but also against pathogenic H37Rv and the clinical MDR and XDR isolates. **B18** is specifically antimycobacterial, as it showed no activity against other common pathogenic bacteria other than *Mtb*, both Gram-positive and Gram-negative species. Our whole-genome sequencing revealed that **B18** resistant mutants and TBA-7371 resistant mutants bear the same point mutation. In addition, the docking study showed that **B18** can bind to the receptor cavity of DprE1. It thus proved that **B18** exerted strong antimycobacterial efficacy by targeting DprE1 in the same way as TBA-7371, demonstrating better target selectivity. Furthermore, **B18** exhibits significantly reduced off-target activity on PDE6C when compared to TBA-7371. Our study suggests that **B18** exhibits benzomorpholine-based selectivity, moderate water solubility, and an ultra-high plasma protein binding rate. Together with the promising drug-like profile of **B18**, these data provide significant confidence for the discovery of a class of DprE1 inhibitors with a completely new structure.

### MATERIALS AND METHODS

### Chemistry

Unless otherwise noted, all materials were obtained from commercial suppliers and used without further purification. Positive compound TBA-7371 (catalog number: HY-19750) was purchased from Selleck. The ^1^H and ^13^C NMR spectra were recorded on a Bruker Avance 400 spectrometer at 25°C using DMSO-d_6_ or CDCl_3_ as the solvent. Chemical shifts (δ) are reported in parts per million (ppm) relative to Me4Si (internal standard), coupling constants (J) are reported in hertz, and peak multiplicity is reported as s (singlet), d (doublet), t (triplet), q (quartet), m (multiplet), or br s (broad singlet). High-resolution mass analysis was performed on a Waters Q-TOF Premier mass spectrometer with electron spray ionization (ESI). Thin layer chromatography (TLC) was performed on 0.20 mm silica gel F-254 plates (Qingdao Haiyang Chemical, China). Visualization of TLC was accomplished with UV light and/or aqueous potassium permanganate or I2 in a silica gel. Column chromatography was performed using silica gel 60 of 300–400 mesh (Qingdao Haiyang Chemical, China).

### The minimum inhibitory concentration determination and resistant mutant generation

*M. tuberculosis* H37Ra was grown at 37°C in Middlebrook 7H9 liquid medium (Difco) supplemented with 0.5% albumin, 0.2% glucose, 0.085% NaCl, 0.2% glycerol, and 0.05% Tween 80 (7H9-ADN-Tw) (17). The MIC of compounds in current research was determined by microplate Alamar blue assay. Briefly, each compound was placed in a 96-well clear bottom plate with twofold dilutions; the culture of *M. tuberculosis* H37Ra was aliquoted into the 96-well plates. The inhibitory concentrations of compounds were determined as previously described, based on color change following overnight incubation after addition of Alamar blue, with pink indicating growth and blue indicating absence of growth (18). The MIC was defined as the concentration of compound that caused 90% inhibition of bacterial growth. Spontaneous *M. tuberculosis* H37Ra resistant mutants were generated by plating 10^8^ cells from a mid-log phase culture on solid media containing either 5×, 10×, or 20× MIC of compounds **B18** and TBA-7371 (17, 19), respectively. Potentially resistant colonies were inoculated into liquid media, cultured to mid-log growth phase, and selected on solid media containing 10× MIC of compounds **B18** and TBA-7371 to confirm phenotypic resistance.

The MICs against *M. tuberculosis* clinical isolates were determined by a microdilution plate assay. Final drug concentration ranges were as follows: for MDR-TB and XDR-TB strains, 0.0156 to 64 µg/mL. TBA-7371 was used as positive control in each experiment; control wells were prepared with bacterial suspension only.

### Sequencing of resistant mutants

Wild-type *M. tuberculosis* H37Ra parent and the *M. tuberculosis* H37Ra resistant mutants of compounds **B18** and TBA-7371 were characterized by WGS (Illumina). Briefly, *M. tuberculosis* H37Ra genomic DNA was extracted by hexadecyl trimethyl ammonium bromide (CTAB), and the purified genomic DNA was determined by NanoDrop 2000 and Qubit 3.0 Fluorometer (Thermo). The DNA libraries were purified using Agencourt AMPure XP beads (Beckman Coulter Genomics). Fragment sizes were determined using an Agilent Technologies 2100 Bioanalyzer with a high sensitivity DNA chip, the libraries were sequenced using Illumina’s high-throughput sequencing platform NovaSeq 6000 (Gene-optimal, Shanghai). Reads were aligned to the reference genome *M. tuberculosis* H37Rv (accession: NC_000962), followed by paired comparison referred to wild-type *M. tuberculosis* H37Ra parent strain.

### The minimum inhibitory concentration determination against other strains

All selected strains were clinically isolated pathogens collected from the Chengdu area and were identified with the collection unit by using a VITEK-60 automatic microbial identification instrument. Cells were cultured in conventional Mueller-Hinton (MH) medium at 35°C–37°C for 18–24 h. The MIC was determined by the agar double dilution method as recommended by the Clinical and Laboratory Standards Institute using the following agar medium: 1% peptone, 0.3% beef powder, 0.5% NaCl, and 1.2% agar powder. One milliliter of the test solution was added to a sterile plate and 14 mL of melted MH(A) medium (~50°C) was added. The final concentrations of the drugs in each plate were 128, 64, 32, 16, 8, 4, 2, 1, 0.5, 0.25, 0.125, 0.06, 0.03, 0.015, 0.008 mg/mL. After cooling, bacteria were inoculated with a multi-point inoculator and the agar plate was covered. The agar plates were incubated at 35°C–37°C for 18–24 h. At the end of the incubation period, the minimum concentration of the sample without bacterial growth on the plate was determined to be the minimum bacteriostatic concentration. A control without drugs and a solvent control with DMSO were also prepared and analyzed using the same procedure described above.

### Cell viability assay

Cells were seeded in 96-well plates at 1,000–3,000 cells per well and treated with compounds **B18** and TBA-7371 for 48 h. Cell viabilities were measured by 3-(4,5-dimethylthiazol-2-yl)−2,5-diphenyltetrazolium bromide (MTT) assay. Briefly, 20 µL of 5 mg/mL MTT solution was added to the culture medium and incubated at 37°C for 3 h. Then 50 µL of 20% (wt/vol) SDS was directly added to each well and incubated at 37°C overnight. The plates were shaken for 15 s and the fluorescence reading were obtained at 570 nm wavelength. The IC_50_ values were calculated using the GraphPad Prism 8 software.

### Protein binding of compounds in mouse plasma

0.1 M sodium phosphate and 0.15 M NaCl buffer (pH = 7.4 ± 0.1) were preheated. The frozen plasma was thawed at 37°C. Plasma was centrifuged at 12,000 rpm for 5 min to remove clots. Pipette and collect the supernatant as the plasma to be used in the experiment. Dialysis membrane strips were soaked in distilled water for an hour. Add 20% by volume ethanol and soak for a further 20 min. The membrane strips were then rinsed in distilled water three times before use. Five microliters of 10 mM stock solution were added into 95 µL of acetonitrile (ACN) for reference and test compounds, which was 0.5 mM solution A. Eight microliters of 10 mM stock solution were added into 192 µL of ACN for reference and test compounds, which was 0.02 mM solution B. A 96-well plate with 380 µL aliquots of plasma was preloaded in the wells designated for plasma, respectively. Twenty microliters of solution B (0.02 mM) were spiked into the pre-loaded plasma in the 96-well plate. The final concentration is 1 µM, containing 0.01% DMSO. Aliquots of 100 µL of blank dialysis buffer were applied to the receiver side of dialysis chambers. Then, aliquots of 100 µL of the plasma spiked with test and reference compounds were applied to the donor side of the dialysis chambers (always add the blank buffer to the receiver first, clearly mark the buffer and plasma chamber holes to avoid cross-contamination). The dialysis block was covered with a plastic lid and the entire apparatus was placed in a shaker (120 rpm) for 5 h at 37°C. At end of incubation, each of post-dialysis samples will be drawn from the buffer and the plasma chambers and processed. All the samples (0, 5 h) were vortexed at 600 rpm for 10 min followed by centrifugation at 6,000 rmp for 15 min. Transfer 100 µL of the supernatant from each well into a 96-well sample plate containing 100 µL of ultrapure water for LC/MS analysis.

### IMAP fluorescence polarization assay for screening compounds against PDE6C

Prepare assay buffer and stop buffer were prepared. PDE6C (BPS, catalog number: 60060) was added in 1× assay buffer, which was the 2× enzyme solution. Fluorescein Amidite - cyclic Guanosine Monophosphate (FAM-cGMP) was added in the 1× assay buffer for PDE6C, which was the 2× substrate solution. Two times enzyme solution was transferred to the assay plate: (i) assay plate already contained corresponding volume of compound in 100% DMSO; (ii) add 2× enzyme solution to each well of the 384-well assay plate; and (iii) incubate at room temperature for 15 min. Two times substrate solution was transferred to the assay plate. PDE reaction and termination: (i) incubate at 25°C for 40 min; (ii) add stop buffer to stop reaction, and incubate at RT for 60 min. Data were collected on Envision (Perkin Elmer). Data were fitted in Excel to obtain inhibition values using equation (1). Equation (1): Inh % = (Max − Signal)/(Max − Min) × 100. Fit the data in XL-Fit to obtain IC_50_ values using equation (2). Equation (2): Y = Bottom + (Top − Bottom)/[1 + (IC_50_/X) × HillSlope]. Y is the % inhibition and X is the compound concentration.

### Aqueous solubility measurement

Aqueous solubility of selected compounds was determined by High Performance Liquid Chromatography (HPLC) analysis. A solution of known concentration of target compound was prepared in methanol by volumetric flask (1,000 µg/mL, 200 µg/mL, 40 µg/mL, 8 µg/mL, 1.6 µg/mL). The absorption intensity of different concentrations of solutions was obtained by HPLC. The linear regression equation between absorption intensity and concentration was obtained by statistical method (*R* ^2^ >0.99). A supersaturated aqueous solution of the target compound was prepared and shaken in 37°C water bath for 24 h. The supersaturated solution was centrifuged and filtered. The absorption intensity of the supersaturated solution was obtained by HPLC. The concentration of saturated aqueous solution was obtained by linear regression equation, which was the aqueous solubility of target compound.

### Chemistry

#### General method for the preparation of A12

To a solution of ethyl 6-bromo-1*H*-indole-4-carboxylate (500 mg, 1.86 mmol) in solvent (9 mL, THF:MeOH:H_2_O = 1:1:1), NaOH (134 mg, 5.58 mmol) was added. The mixture was stirred at 40°C for 4 h and monitored by TLC. Upon completion, the mixture was concentrated in reduced pressure. Subsequently, the residue was dissolved in methanol and filtered with celite. The organic phase was concentrated to afford the title compound (423 mg, 95%) without further purification. ^1^H NMR (400 MHz, DMSO-*d*_6_) δ 12.95 (s, 1H), 11.98–11.01 (m, 1H), 7.83 (d, *J* = 1.7 Hz, 1H), 7.75 (d, *J* = 1.8 Hz, 1H), 7.54 (t, *J* = 2.8 Hz, 1H), 6.95 (t, *J* = 2.5 Hz, 1H).

#### General method for the preparation of A13

To a solution of **A12** (200 mg, 0.83 mmol) and 2-fluoroethylamine hydrochloride (100 mg, 1 mmol) in DMSO (6 mL) was added EDCI (286 mg, 1.49 mmol), HOAt (203 mg, 1.49 mmol), and NMM (0.46 mL, 4.15 mmol). The mixture was stirred at ambient temperature overnight. Upon completion, the mixture was diluted with ethyl acetate and washed three times with H_2_O. The organic phase was concentrated in reduced pressure and purified by biotage column chromatography (10%–30% ethyl acetate in hexane) to afford title compound (236 mg, quant yield). ^1^H NMR (400 MHz, DMSO-*d*_6_) δ 11.41 (s, 1H), 8.58 (t, *J* = 5.6 Hz, 1H), 7.72 (dd, *J* = 1.7, 0.9 Hz, 1H), 7.56 (d, *J* = 1.7 Hz, 1H), 7.48 (t, *J* = 2.8 Hz, 1H), 7.01–6.79 (m, 1H), 4.63 (t, *J* = 5.2 Hz, 1H), 4.51 (t, *J* = 5.2 Hz, 1H), 3.62 (q, *J* = 5.3 Hz, 1H), 3.56 (q, *J* = 5.3 Hz, 1H).

#### 6-bromo-*N*-(2,2-difluoroethyl)-1*H*-indole-4-carboxamide (A14)

Prepared from the general methods for **A13** by replacing 2-fluoroethylamine hydrochloride with 2,2-difluoroethylamine hydrochloride. ^1^H NMR (400 MHz, DMSO-*d*_6_) δ 11.45 (s, 1H), 8.75 (t, *J* = 5.9 Hz, 1H), 8.04–7.68 (m, 1H), 7.59 (d, *J* = 1.7 Hz, 1H), 7.50 (t, *J* = 2.8 Hz, 1H), 6.86 (t, *J* = 2.5 Hz, 1H), 6.41–5.91 (m, 1H), 3.81–3.55 (m, 2H).

#### General method for the preparation of A1

To a solution of **A13** (118 mg, 0.41 mmol) and 2,3-difluoro-6-methoxybenzyl bromide (118 mg, 0.49 mmol) in DMF (6 mL), K_2_CO_3_ (170 mg, 1.23 mmol) was added. The mixture was stirred at ambient temperature for 3 h and monitored by TLC. Upon completion, the mixture was diluted with ethyl acetate and washed three times with H_2_O. The organic phase was concentrated in reduced pressure and purified by biotage column chromatography (10%–30% ethyl acetate in hexane) to afford title compound (145 mg, 80.2%). ^1^H NMR (400 MHz, chloroform-*d*) δ 7.94–7.82 (m, 1H), 7.62–7.53 (m, 1H), 7.36–7.32 (m, 1H), 7.09 (q, *J* = 9.4 Hz, 1H), 6.80 (d, *J* = 3.2 Hz, 1H), 6.66–6.58 (m, 1H), 6.51 (s, 1H), 5.33 (d, *J* = 2.0 Hz, 2H), 4.69 (t, *J* = 4.8 Hz, 1H), 4.57 (t, *J* = 4.8 Hz, 1H), 3.89–3.87 (m, 3H), 3.86–3.75 (m, 2H). ^13^C NMR (101 MHz, chloroform-*d*) δ 167.37, 137.67, 130.92, 127.78, 124.97, 122.40, 116.06, 114.49, 101.30, 83.73, 82.08, 56.10, 40.41, 38.23. MS(ESI): calcd for C_19_H_17_BrF_3_N_2_O_2_^+^, *m/z*, 441.0.

#### 6-bromo-1-(2,3-difluoro-6-methoxybenzyl)-*N*-(2,2-difluoroethyl)-1*H*-indole-4-carboxamide (A2)

Prepared from the general methods for **A1**. ^1^H NMR (400 MHz, chloroform-*d*) δ 7.99–7.80 (m, 1H), 7.59 (t, *J* = 1.4 Hz, 1H), 7.42–7.33 (m, 1H), 7.16–7.01 (m, 1H), 6.85–6.70 (m, 1H), 6.67–6.50 (m, 1H), 6.39 (s, 1H), 6.18–5.77 (m, 1H), 5.42–5.20 (m, 2H), 3.95–3.79 (m, 5H). ^13^C NMR (101 MHz, chloroform-*d*) δ 167.60, 137.68, 131.14, 127.01, 125.00, 122.52, 116.99, 116.79, 116.40, 114.44, 113.68, 105.93, 101.22, 56.11, 42.09. MS(ESI): calcd for C_19_H_16_BrF_4_N_2_O_2_^+^, [M + H]^+^, *m/z*, 459.0.

#### 6-bromo-*N*-(2,2-difluoroethyl)-1-((3,5,6-trimethylpyrazin-2-yl)methyl)-1*H*-indole-4-carboxamide (A3)

Prepared from the general methods for **A1**. ^1^H NMR (400 MHz, chloroform-*d*) δ 7.83 (s, 1H), 7.68–7.54 (m, 1H), 7.21 (d, *J* = 3.3 Hz, 1H), 6.83 (d, *J* = 3.2 Hz, 1H), 6.43 (s, 1H), 6.21–5.79 (m, 1H), 5.36 (s, 2H), 4.01–3.82 (m, 2H), 2.50 (s, 6H), 2.44 (s, 3H). ^13^C NMR (101 MHz, chloroform-*d*) δ 151.45, 144.85, 137.96, 130.65, 122.73, 116.51, 115.69, 114.58, 113.67, 111.27, 101.49, 50.23, 21.62, 21.46, 20.85. MS(ESI): calcd for C_19_H_20_BrF_2_N_2_O^+^, [M + H]^+^, *m/z*, 437.1.

#### 6-bromo-*N*-(2,2-difluoroethyl)-1-((6-methoxy-5-methylpyrimidin-4-yl)methyl)-1*H*-indole-4-carboxamide (A4)

Prepared from the general methods for **A1**. ^1^H NMR (400 MHz, chloroform-*d*) δ 8.57 (s, 1H), 7.68–7.64 (m, 1H), 7.60 (t, *J* = 1.4 Hz, 1H), 6.92–6.82 (m, 1H), 6.42 (s, 1H), 6.22–5.80 (m, 1H), 5.32 (s, 2H), 4.00 (d, *J* = 1.1 Hz, 3H), 3.95–3.80 (m, 2H), 2.17 (s, 3H). ^13^C NMR (101 MHz, chloroform-*d*) δ 168.25, 160.11, 155.53, 137.97, 131.04, 127.35, 125.25, 122.88, 116.01, 115.97, 114.75, 107.03, 101.66, 54.31, 49.21, 10.09. MS(ESI): calcd for C_18_H_18_BrF_2_N_4_O_2_^+^, [M + H]^+^, *m/z*, 439.1.

#### 6-bromo-*N*-(2,2-difluoroethyl)-1-((2-methoxy-5-(trifluoromethyl)pyridin-3-yl)methyl)-1*H*-indole-4-carboxamide (A5)

Prepared from the general methods for **A1**. ^1^H NMR (400 MHz, chloroform-*d*) δ 8.39 (d, *J* = 2.3 Hz, 1H), 7.63 (d, *J* = 1.5 Hz, 1H), 7.57 (d, *J* = 1.5 Hz, 1H), 7.25 (d, *J* = 3.3 Hz, 1H), 7.13–7.05 (m, 1H), 6.94 (d, *J* = 3.2 Hz, 1H), 6.43 (s, 1H), 6.23–5.80 (m, 1H), 5.29 (s, 2H), 4.10 (d, *J* = 1.1 Hz, 3H), 3.97–3.81 (m, 2H). ^13^C NMR (101 MHz, chloroform-*d*) δ 167.42, 144.37, 137.61, 130.60, 127.59, 125.42, 122.96, 119.92, 115.66, 115.01, 113.65, 102.41, 54.48, 44.80. MS(ESI): calcd for C_19_H_16_BrF_5_N_3_O_2_^+^, [M + H]^+^, *m/z*, 492.0.

#### General method for the preparation of A6

CH_3_BF_3_K (120 mg, 0.27 mmol), **A1** (99 mg, 0.81 mmol), Pd(dppf)Cl_2_ (12 mg, 0.027 mmol), and K_2_CO_3_ (0.77 mg, 0.54 mmol) were added in 1,4-dioxane (4 mL) and H_2_O (1 mL). The mixture was sparged with nitrogen for 15 min and heated under nitrogen at 110°C for 1 day. Upon completion, the reaction mixture was concentrated to remove solvent. The residue was filtered through celite and washed with solvent (hexane/ethyl acetate = 1/1). The filtrate was concentrated and purified by biotage column chromatography (20%–50% ethyl acetate in hexanes) to afford the title compound as brown liquid (53 mg, 53%). ^1^H NMR (400 MHz, chloroform-*d*) δ 6.97 (d, *J* = 2.1 Hz, 1H), 6.53 (d, *J* = 2.1 Hz, 1H), 4.44–4.22 (m, 2H), 3.87 (s, 3H), 3.57–3.25 (m, 2H), 2.20 (s, 3H).

#### 1-(2,3-difluoro-6-methoxybenzyl)-*N*-(2,2-difluoroethyl)-6-methyl-1*H*-indole-4-carboxamide (A7)

Prepared from the general methods for **A6**. ^1^H NMR (400 MHz, chloroform-*d*) δ 7.54 (s, 1H), 7.36 (s, 1H), 7.30 (d, *J* = 3.0 Hz, 1H), 7.12–7.01 (m, 1H), 6.72 (d, *J* = 3.2 Hz, 1H), 6.63–6.52 (m, 1H), 6.44 (t, *J* = 6.3 Hz, 1H), 6.19–5.71 (m, 1H), 5.33 (t, *J* = 1.5 Hz, 2H), 3.93–3.79 (m, 5H), 2.52 (s, 3H). ^13^C NMR (101 MHz, chloroform-*d*) δ 168.96, 154.01, 137.30, 131.02, 130.00, 125.39, 123.83, 121.37, 116.66, 116.48, 114.84, 114.70, 113.88, 113.43, 113.40, 100.43, 56.09, 42.05, 41.79, 21.77. MS(ESI): calcd for C_20_H_19_F_4_N_2_O_2_^+^, [M + H]^+^, *m/z*, 395.1.

#### *N*-(2,2-difluoroethyl)-6-methyl-1-((3,5,6-trimethylpyrazin-2-yl)methyl)-1*H*-indole-4-carboxamide (A8)

Prepared from the general methods for **A6**. ^1^H NMR (400 MHz, chloroform-*d*) δ 7.43 (s, 1H), 7.37 (s, 1H), 7.14 (d, *J* = 3.3 Hz, 1H), 6.77 (d, *J* = 3.3 Hz, 1H), 6.48 (d, *J* = 6.5 Hz, 1H), 6.23–5.79 (m, 1H), 5.36 (s, 2H), 3.88 (tdt, *J* = 15.0, 10.6, 4.7 Hz, 2H), 2.53–2.46 (m, 6H), 2.40 (s, 3H). ^13^C NMR (101 MHz, chloroform-*d*) δ 168.90, 151.13, 149.08, 148.61, 145.48, 137.57, 131.23, 129.32, 125.61, 124.14, 121.55, 113.52, 100.89, 50.09, 21.61, 21.47, 20.86. MS(ESI): calcd for C_20_H_23_F_2_N_4_O^+^, [M + H]^+^, *m/z*, 373.2.

#### *N*-(2,2-difluoroethyl)-1-((6-methoxy-5-methylpyrimidin-4-yl)methyl)-6-methyl-1*H*-indole-4-carboxamide (A9)

Prepared from the general methods for **A6**. ^1^H NMR (400 MHz, chloroform-*d*) δ 8.59 (s, 1H), 7.37 (s, 1H), 7.30 (s, 1H), 7.20 (d, *J* = 3.2 Hz, 1H), 6.81 (d, *J* = 3.3 Hz, 1H), 6.46 (s, 1H), 6.21–5.78 (m, 1H), 5.33 (s, 2H), 3.99 (s, 3H), 3.95–3.80 (m, 2H), 2.47 (s, 3H), 2.14 (s, 3H). ^13^C NMR (101 MHz, chloroform-*d*) δ 168.87, 168.25, 160.78, 155.42, 137.55, 131.42, 129.72, 125.74, 124.05, 121.70, 116.15, 113.22, 101.05, 54.24, 49.28, 42.07, 21.69, 10.05. MS(ESI): calcd for C_19_H_21_F_2_N_4_O_2_^+^, [M + H]^+^, *m/z*, 375.2.

#### *N*-(2,2-difluoroethyl)-1-((2-methoxy-5-(trifluoromethyl)pyridin-3-yl)methyl)-6-methyl-1*H*-indole-4-carboxamide (A10)

Prepared from the general methods for **A6**. ^1^H NMR (400 MHz, chloroform-*d*) δ 8.36 (s, 1H), 7.39 (s, 1H), 7.20 (t, *J* = 2.8 Hz, 1H), 7.17 (s, 1H), 7.04 (s, 1H), 6.89 (d, *J* = 3.1 Hz, 1H), 6.48 (d, *J* = 6.4 Hz, 1H), 6.23–5.80 (m, 1H), 5.29 (s, 2H), 4.10 (d, *J* = 2.3 Hz, 3H), 4.02–3.76 (m, 2H), 2.47 (d, *J* = 2.3 Hz, 3H). ^13^C NMR (101 MHz, chloroform-*d*) δ 168.83, 162.97, 137.22, 132.63, 131.75, 129.48, 125.97, 124.28, 121.76, 120.62, 113.85, 112.83, 111.45, 101.73, 54.39, 44.58, 42.10, 21.64. MS(ESI): calcd for C_20_H_19_F_5_N_3_O_2_^+^, [M + H]^+^, *m/z*, 428.1.

#### General method for the preparation of B35

To a solution of methyl 3-amino-5-bromo-2-hydroxybenzoate (4.92 g, 20 mmol) and K_2_CO_3_ (5.52 g, 40 mmol) in DMF (23 mL), 1,2-dibromoethane (8.6 mL, 100 mmol) was added. The mixture was stirred at 80°C for 8 h and monitored by TLC. Upon completion, the reaction mixture was poured into ice cold water. The solid formed was filtered and dried in reduced pressure to afford title compound as brown solid (5.2 g, 95.6%). ^1^H NMR (400 MHz, chloroform-*d*) δ 7.24 (d, *J* = 2.4 Hz, 1H), 6.81 (d, *J* = 2.4 Hz, 1H), 4.35–4.28 (m, 2H), 3.99 (s, 1H), 3.87 (s, 3H), 3.48–3.41 (m, 2H).

#### General method for the preparation of B36

To a solution of **B35** (272 mg, 1 mmol) and Na_2_CO_3_ (212 mg, 2 mmol) in DMF (6 mL) was added 2-(bromomethyl)-3,5,6-trimethylpyrazine (215 mg, 1 mmol). The mixture was stirred at 80°C for 3 h. Upon completion, the reaction mixture was poured into ice cold water, extracted with ethyl acetate. The organic layer was dried with Na_2_SO_4_, purified by biotage column chromatography (30%–60% ethyl acetate in hexanes) to afford the title compound as brown liquid (252 mg, 62%). ^1^H NMR (400 MHz, chloroform-*d*) δ 7.20 (d, *J* = 2.4 Hz, 1H), 6.98 (d, *J* = 2.3 Hz, 1H), 4.47 (s, 2H), 4.33 (t, 2H), 3.86 (s, 3H), 3.44 (t, *J* = 4.5 Hz, 2H), 2.52 (s, 3H), 2.49 (s, 3H), 2.46 (s, 3H).

#### General method for the preparation of B38

CH_3_BF_3_K (1.22 g, 10 mmol), **B35** (1.36 g, 5 mmol), Pd(dppf)Cl_2_ (0.366 g, 0.5 mmol), and K_2_CO_3_ (1.73 g, 12.5 mmol) were added in 1,4-dioxane (12 mL) and H_2_O (4 mL). The mixture was sparged with nitrogen for 15 min and heated under nitrogen at 110°C for 1 day. Upon completion, the reaction mixture was concentrated to remove solvent. The residue was filtered through celite and washed with solvent (hexane/ethyl acetate = 1/1). The filtrate was concentrated and purified by biotage column chromatography (20%–50% ethyl acetate in hexanes) to afford the title compound as brown liquid (589 mg, 57%). ^1^H NMR (400 MHz, chloroform-*d*) δ 6.97 (d, *J* = 2.1 Hz, 1H), 6.53 (d, *J* = 2.1 Hz, 1H), 4.44–4.22 (m, 2H), 3.87 (s, 3H), 3.57–3.25 (m, 2H), 2.20 (s, 3H).

#### General method for the preparation of B41

Pd(OAc)_2_ (473 mg, 2.1 mmol), **B35** (1.9 g, 7 mmol), cyclopropylboronic acid (0.96 g, 11.2 mmol), (C_6_H_11_)_3_P (588 mg, 2.1 mmol), and K_3_PO_4_ (4.45 g, 21 mmol) were added in PhMe (22.5 mL) and H_2_O (0.5 mL). The mixture was sparged with nitrogen for 15 min and heated under nitrogen at 100°C for 3 h. Upon completion, the reaction mixture was concentrated to remove solvent. The residue was filtered through celite and washed with solvent (hexane/ethyl acetate = 1/1). The filtrate was concentrated and purified by biotage column chromatography (20%–50% ethyl acetate in hexanes) to afford the title compound as brown liquid (1.2 g, 64%).

#### General method for the preparation of B37

A solution of **B36** (120 mg, 0.29 mmol) and NaOH (27 mg, 0.58 mmol) in solvent (6 mL, THF:MeOH:H_2_O = 1:1:1) was stirred at 70°C for 1 h. Upon completion, the mixture was concentrated in reduced pressure to afford the title compound without further purification.

#### General method for the preparation of B2

To a solution of **B37** (40 mg, 0.1 mmol), 2,2-difluoroethylamine hydrochloride (12 mg, 0.1 mmol), and HATU (38 mg, 0.1 mmol) in DMF (3 mL), DIPEA (40 mg, 0.3 mmol) was added. The mixture was stirred at ambient temperature for 4 h. Upon completion, the mixture was concentrated to remove solvent. The residue was purified by biotage column chromatography (3%–5% MeOH in dichloromethane) to afford title compound as white solid (38.8 mg, 85.2%). ^1^H NMR (400 MHz, DMSO-*d*_6_) δ 8.49 (t, *J* = 6.0 Hz, 1H), 7.05 (d, *J* = 2.4 Hz, 1H), 6.99 (d, *J* = 2.3 Hz, 1H), 6.10 (m, 1H), 4.62 (s, 2H), 4.35–4.28 (m, 2H), 3.66 (m, 2H), 3.47 (m, 2H), 2.47 (s, 3H), 2.41 (s, 3H), 2.38 (s, 3H). ^13^C NMR (101 MHz, DMSO-*d*_6_) δ 165.27, 149.79, 148.97, 148.31, 146.76, 141.53, 138.10, 123.59, 119.49, 116.48, 115.02, 113.03, 65.14, 53.30, 46.86, 21.72, 21.51, 20.74. High Resolution Microwave Survey (HRMS). Direct analysis in real time - Time of Flight (DART-TOF). HRMS (DART-TOF): calcd for C_19_H_22_BrF_2_N_2_O_4_^+^, [M + H]^+^, *m/z*, 455.0845.

#### General method for the preparation of B45

Phenylboronic acid (0.45 g, 3.7 mmol), **B37** (1 g, 2.5 mmol), Pd(dppf)Cl_2_ (0.18 g, 0.25 mmol), and Na_2_CO_3_ (0.78 g, 7.4 mmol) were added in 1,4-dioxane (12 mL) and H_2_O (3 mL). The mixture was sparged with nitrogen for 15 min and heated under nitrogen at 100°C for 6 h. Upon completion, the reaction mixture was concentrated to remove solvent. The residue was filtered through celite and washed with solvent (DCM/MeOH = 10/1). The filtrate was concentrated and purified by biotage column chromatography (20%–50% ethyl acetate in hexanes) to afford the title compound as brown liquid (685 mg, 68%). ^1^H NMR (400 MHz, chloroform-*d*) δ 7.53–7.46 (m, 2H), 7.42–7.35 (m, 3H), 7.34–7.22 (m, 2H), 4.55 (s, 2H), 4.40 (t, *J* = 4.4 Hz, 2H), 3.89 (s, 3H), 3.49 (d, *J* = 3.9 Hz, 2H), 2.55 (s, 3H), 2.49 (s, 6H).

#### General method for the preparation of B33

A solution of **B45** (403 mg, 1 mmol) and NaOH (120 mg, 3 mmol) in solvent (9 mL, THF:MeOH:H_2_O = 1:1:1) was stirred at 70°C for 1 h. Upon completion, the mixture was concentrated in reduced pressure.

The residue was dissolved in DMF (8 mL). Then, HATU (570 mg), DIPEA (520 µL, 3 mmol), and 2,2,2-trifluoroethylamine hydrochloride (203 mg, 1.5 mmol) were added to the solution and the mixture was stirred for 2 h. Upon completion, the mixture was concentrated to remove solvent. The residue was purified by biotage column chromatography (3%–5% MeOH in dichloromethane) to afford title compound as white solid (390 mg, 83%). ^1^H NMR (400 MHz, chloroform-*d*) δ 8.16 (t, *J* = 6.2 Hz, 1H), 7.81 (d, *J* = 2.2 Hz, 1H), 7.56–7.49 (m, 2H), 7.39 (dd, *J* = 8.4, 6.8 Hz, 2H), 7.34–7.23 (m, 2H), 4.57 (s, 2H), 4.48 (t, *J* = 4.4 Hz, 2H), 4.22–4.09 (m, 2H), 3.55 (t, *J* = 4.5 Hz, 2H), 2.56 (s, 3H), 2.49 (s, 6H). ^13^C NMR (101 MHz, chloroform-*d*) δ 207.34, 197.73, 185.59, 165.93, 148.69, 146.66, 144.43, 142.55, 140.44, 136.01, 134.50, 134.35, 128.66, 127.06, 126.77, 120.03, 119.29, 115.16, 65.64, 64.83, 59.12, 55.11, 47.12, 21.53, 19.31. MS(ESI): calcd for C_25_H_26_F_3_N_4_O_2_^+^, [M + H]^+^, *m/z*, 471.2.

#### 6-bromo-*N*-(2-fluoroethyl)−4-((3,5,6-trimethylpyrazin-2-yl)methyl)−3,4-dihydro-2*H*-benzo[2, 4]oxazine-8-carboxamide (B1)

Prepared from the general methods for **B35–37** and **B2**. ^1^H NMR (400 MHz, chloroform-*d*) δ 8.06 (s, 1H), 7.60 (d, *J* = 2.4 Hz, 1H), 6.97 (d, *J* = 2.4 Hz, 1H), 4.65 (t, *J* = 4.8 Hz, 1H), 4.53 (t, *J* = 4.8 Hz, 1H), 4.48 (s, 2H), 4.43–4.36 (m, 2H), 3.76 (m, 2H), 3.51–3.44 (m, 2H), 2.56–2.43 (m, 9H). ^13^C NMR (101 MHz, DMSO-*d*_6_) δ 164.88, 149.78, 148.96, 148.31, 146.81, 141.40, 138.06, 124.20, 119.51, 116.25, 112.99, 81.81, 65.08, 53.33, 46.89, 21.72, 21.50, 20.72. MS(ESI): calcd for C_19_H_23_BrFN_4_O_2_^+^, [M + H]^+^, *m/z*, 437.1.

#### 6-bromo-*N*-(2-fluoroethyl)-4-((2-methoxy-5-(trifluoromethyl)pyridin-3-yl)methyl)-3,4-dihydro-2*H*-benzo[2, 4]oxazine-8-carboxamide (B3)

Prepared from the general methods for **B35–37** and **B2**. ^1^H NMR (400 MHz, chloroform-*d*) δ 8.40 (d, *J* = 2.2 Hz, 1H), 8.03 (m, 1H), 7.63 (d, *J* = 2.4 Hz, 1H), 7.57 (d, *J* = 2.3 Hz, 1H), 6.73 (d, *J* = 2.4 Hz, 1H), 4.66 (t, *J* = 4.8 Hz, 1H), 4.54 (t, *J* = 4.8 Hz, 1H), 4.49–4.42 (m, 2H), 4.40 (s, 2H), 4.08 (s, 3H), 3.81 (q, *J* = 5.1 Hz, 1H), 3.74 (q, *J* = 5.1 Hz, 1H), 3.54–3.47 (m, 2H). ^13^C NMR (101 MHz, chloroform-*d*) δ 164.43, 163.64, 143.72, 141.72, 136.43, 132.46, 122.83, 122.42, 119.68, 117.69, 114.56, 83.71, 82.06, 64.95, 54.33, 50.80, 47.22, 40.30. HRMS (DART-TOF): calcd for C_19_H_19_BrF_4_N_3_O_3_^+^, [M + H]^+^, *m/z*, 492.0536.

#### 6-bromo-*N*-(2-hydroxyethyl)-4-((2-methoxy-5-(trifluoromethyl)pyridin-3-yl)methyl)-3,4-dihydro-2*H*-benzo[2, 4]oxazine-8-carboxamide (B4)

Prepared from the general methods for **B35–37** and **B2**. ^1^H NMR (400 MHz, chloroform-*d*) δ 8.41 (d, *J* = 2.3 Hz, 1H), 8.06 (s, 1H), 7.63 (d, *J* = 2.4 Hz, 1H), 7.57 (d, *J* = 2.4 Hz, 1H), 6.74 (d, *J* = 2.4 Hz, 1H), 4.43 (t, *J* = 4.5 Hz, 2H), 4.40 (s, 2H), 4.08 (s, 3H), 3.83 (s, 2H), 3.63 (m, 2H), 3.50 (t, *J* = 4.5 Hz, 2H). ^13^C NMR (101 MHz, chloroform-*d*) δ 165.63–165.54 (m), 163.65, 143.75, 141.67, 136.39, 132.50, 132.47, 122.84, 122.44, 119.66, 117.73, 117.72, 114.58, 64.97, 62.82, 54.31, 50.78, 47.21, 43.04. HRMS (DART-TOF): calcd for C_19_H_20_BrF_3_N_3_O_4_^+^, [M + H]^+^, *m/z*, 490.0609.

#### 6-bromo-*N*-(2,2-difluoroethyl)-4-((2-methoxy-5-(trifluoromethyl)pyridin-3-yl)methyl)-3,4-dihydro-2*H*-benzo[b][1,4]oxazine-8-carboxamide (B5)

Prepared from the general methods for **B35–37** and **B2**. ^1^H NMR (400 MHz, chloroform-*d*) δ 8.41 (d, *J* = 2.0 Hz, 1H), 7.96 (s, 1H), 7.63 (d, *J* = 2.4 Hz, 1H), 7.60–7.54 (m, 1H), 6.75 (d, *J* = 2.4 Hz, 1H), 5.96 (m, 1H), 4.48–4.43 (m, 2H), 4.41 (s, 2H), 4.08 (s, 3H), 3.83 (m, 2H), 3.51 (t, *J* = 4.5 Hz, 2H). MS(ESI): calcd for C_19_H_18_BrF_5_N_3_O_3_^+^, [M + H]^+^, *m/z*, 510.0.

#### 6-bromo-4-(2,3-difluoro-6-methoxybenzyl)-*N*-(2-fluoroethyl)-3,4-dihydro-2*H*-benzo[b][1,4]oxazine-8-carboxamide (B6)

Prepared from the general methods for **B35–37** and **B2**. ^1^H NMR (400 MHz, chloroform-*d*) δ 8.04 (s, 1H), 7.58 (d, *J* = 2.4 Hz, 1H), 7.26 (s, 1H), 7.09 (m, 1H), 6.62 (m, 1H), 4.63 (t, *J* = 4.8 Hz, 1H), 4.51 (t, *J* = 4.8 Hz, 1H), 4.45 (d, *J* = 1.7 Hz, 2H), 4.31 (t, *J* = 4.4 Hz, 2H), 3.89 (s, 3H), 3.78 (m, 1H), 3.74–3.67 (m, 1H), 3.47 (t, *J* = 4.4 Hz, 2H). ^13^C NMR (101 MHz, chloroform-*d*) δ 164.71, 151.01, 141.81, 136.42, 128.94, 121.96, 121.94, 120.38, 118.63, 116.31, 114.49, 114.32–114.12 (m), 105.72–105.40, 83.72, 82.07, 64.99, 56.17, 46.36, 44.15. HRMS (DART-TOF): calcd for C_19_H_19_BrF_3_N_2_O_3_^+^, [M + H]^+^, *m/z*, 459.0515.

#### 6-bromo-4-(2,3-difluoro-6-methoxybenzyl)-*N*-(2,2-difluoroethyl)-3,4-dihydro-2*H*-benzo[b][1,4]oxazine-8-carboxamide (B7)

Prepared from the general methods for **B35–37** and **B2**. ^1^H NMR (400 MHz, chloroform-d) δ 8.01 (d, *J* = 6.4 Hz, 1H), 7.56 (d, *J* = 2.4 Hz, 1H), 7.05 (d, *J* = 9.3 Hz, 1H), 6.72–6.54 (m, 2H), 6.11–5.74 (m, 1H), 4.76 (d, *J* = 2.0 Hz, 2H), 4.45 (d, *J* = 1.8 Hz, 2H), 4.31 (t, *J* = 4.4 Hz, 2H), 3.89 (s, 3H), 3.48 (t, *J* = 4.4 Hz, 2H). ^13^C NMR (101 MHz, chloroform-d) δ 165.12 , 147.18–145.39 (m), 144.68–143.76 (m), 141.88 , 136.44 , 121.87 , 121.25 , 118.85 , 116.52–116.08 (m), 116.04–115.21 (m), 114.66–114.34 (m), 114.30 , 105.43–105.03 (m), 65.09 , 57.81–55.55 (m), 54.19–53.74 (m), 48.17–45.69 (m), 44.11. HRMS (DART-TOF): calcd for C_19_H_18_BrF_4_N_2_O_3_^+^, [M + H]^+^, *m/z*, 477.0435.

#### 6-bromo-4-(2,3-difluoro-6-methoxybenzyl)-*N*-(2-hydroxyethyl)-3,4-dihydro-2*H*-benzo[b][1,4]oxazine-8-carboxamide (B8)

Prepared from the general methods for **B35–37** and **B2**. ^1^H NMR (400 MHz, chloroform-*d*) δ 8.08 (s, 1H), 7.58 (d, *J* = 2.4 Hz, 1H), 7.27 (s, 1H), 7.09 (m, 1H), 6.62 (m, 1H), 4.46 (d, *J* = 1.7 Hz, 2H), 4.31 (t, *J* = 4.4 Hz, 2H), 3.89 (s, 3H), 3.80 (t, *J* = 4.9 Hz, 2H), 3.59 (m, 2H), 3.52–3.43 (m, 2H), 2.86 (s, 1H). HRMS (DART-TOF): calcd for C_19_H_20_BrF_2_N_2_O_4_^+^, [M + H]^+^, *m/z*, 457.0559.

#### 6-bromo-4-(3,4-difluoro-2-methoxybenzyl)-*N*-(2,2-difluoroethyl)-3,4-dihydro-2*H*-benzo[b][1,4]oxazine-8-carboxamide (B9)

Prepared from the general methods for **B35–37** and **B2**. ^1^H NMR (400 MHz, chloroform-*d*) δ 7.99 (s, 1H), 7.59 (d, *J* = 2.4 Hz, 1H), 6.94 (m, 1H), 6.79 (d, *J* = 2.4 Hz, 1H), 6.74 (m, 1H), 5.96 (m, 1H), 4.41 (t, *J* = 4.5 Hz, 2H), 4.36 (s, 2H), 3.84 (s, 3H), 3.83–3.75 (m, 2H), 3.48 (t, *J* = 4.4 Hz, 2H). ^13^C NMR (101 MHz, chloroform-*d*) δ 164.92, 141.68, 136.66, 122.24, 121.54, 120.43, 117.94, 116.18, 116.05, 115.86, 114.66, 113.78, 100.92, 100.71, 65.08, 56.05, 50.20, 46.80, 42.18. HRMS (DART-TOF): calcd for C_19_H_18_BrF_4_N_2_O_3_^+^, [M + H]^+^, *m/z*, 477.0435.

#### 6-bromo-4-(3,4-difluoro-2-methoxybenzyl)-*N*-(2-hydroxyethyl)-3,4-dihydro-2*H*-benzo[b][1,4]oxazine-8-carboxamide (B10)

Prepared from the general methods for **B35–37** and **B2**. ^1^H NMR (400 MHz, chloroform-*d*) δ 8.09 (s, 1H), 7.59 (d, *J* = 2.4 Hz, 1H), 6.94 (m, 1H), 6.80–6.69 (m, 2H), 4.40 (m, 2H), 4.36 (s, 2H), 3.84 (s, 3H), 3.82 (m, 2H), 3.62 (m, 2H), 3.47 (q, *J* = 4.5, 3.8 Hz, 2H). HRMS (DART-TOF): calcd for C_19_H_20_BrF_2_N_2_O_4_^+^, [M + H]^+^, *m/z*, 457.0563.

#### *N*-(2,2-difluoroethyl)-6-methyl-4-((3,5,6-trimethylpyrazin-2-yl)methyl)-3,4-dihydro-2*H*-benzo[b][1,4]oxazine-8-carboxamide (B11)

Prepared from the general methods for **B35–38** and **B11**. ^1^H NMR (400 MHz, chloroform-*d*) δ 8.09 (s, 1H), 7.33 (d, *J* = 2.0 Hz, 1H), 6.76 (d, *J* = 2.1 Hz, 1H), 5.96 (m, 1H), 4.49 (s, 2H), 4.37 (t, *J* = 4.4 Hz, 2H), 3.81 (m, 2H), 3.40 (t, *J* = 4.5 Hz, 2H), 2.55 (s, 3H), 2.50 (s, 3H), 2.47 (s, 3H), 2.23 (s, 3H). ^13^C NMR (101 MHz, chloroform-*d*) δ 166.45, 150.17, 148.80, 148.66, 146.66, 141.05, 135.43, 130.71, 120.73, 119.74, 117.04, 113.97, 65.23, 54.44, 46.56, 21.56, 21.08, 20.77. HRMS (DART-TOF): calcd for C_20_H_25_F_2_N_4_O_2_^+^, [M + H]^+^, *m/z*, 391.1950.

#### 6-methyl-*N*-(2,2,2-trifluoroethyl)-4-((3,5,6-trimethylpyrazin-2-yl)methyl)-3,4-dihydro-2*H*-benzo[b][1,4]oxazine-8-carboxamide (B12)

Prepared from the general methods for **B35–38** and **B11**. ^1^H NMR (400 MHz, chloroform-*d*) δ 8.18 (t, *J* = 6.2 Hz, 1H), 7.34 (d, *J* = 1.8 Hz, 1H), 6.76 (d, *J* = 2.0 Hz, 1H), 4.49 (s, 2H), 4.46–4.35 (m, 2H), 4.12 (m, 2H), 3.50–3.32 (m, 2H), 2.55 (s, 3H), 2.50 (s, 3H), 2.47 (s, 3H), 2.23 (s, 3H). ^13^C NMR (101 MHz, chloroform-*d*) δ 166.16, 150.16, 148.81, 148.62, 146.61, 141.01, 135.44, 130.78, 120.84, 119.47, 117.17, 65.26, 54.38, 46.56, 21.53, 21.06, 20.74. HRMS (DART-TOF): calcd for C_20_H_24_F_3_N_4_O_2_^+^, [M + H]^+^, *m/z*, 409.1840.

#### *N*-(2-hydroxyethyl)-6-methyl-4-((3,5,6-trimethylpyrazin-2-yl)methyl)-3,4-dihydro-2*H*-benzo[b][1,4]oxazine-8-carboxamide (B13)

Prepared from the general methods for **B35–38** and **B11**. ^1^H NMR (400 MHz, chloroform-*d*) δ 8.21 (t, *J* = 5.6 Hz, 1H), 7.29 (d, *J* = 15.5 Hz, 1H), 6.81–6.72 (m, 1H), 4.47 (d, *J* = 10.8 Hz, 2H), 4.35 (t, *J* = 4.5 Hz, 2H), 3.81 (t, *J* = 4.8 Hz, 2H), 3.60 (m, 2H), 3.37 (t, *J* = 4.5 Hz, 2H), 2.54 (s, 3H), 2.49 (s, 3H), 2.47 (s, 3H), 2.23 (s, 3H). ^13^C NMR (101 MHz, chloroform-*d*) δ 167.46, 150.14, 148.79, 148.70, 146.73, 140.94, 135.36, 130.58, 120.70, 120.27, 116.83, 65.17, 62.99, 54.47, 46.54, 43.11, 21.52, 21.09, 20.73. HRMS (DART-TOF): calcd for C_20_H_27_N_4_O_3_^+^, [M + H]^+^, *m/z*, 371.2078.

#### *N*-cyclopropyl-6-methyl-4-((3,5,6-trimethylpyrazin-2-yl)methyl)-3,4-dihydro-2*H*-benzo[b][1,4]oxazine-8-carboxamide (B14)

Prepared from the general methods for **B35–38** and **B11**. ^1^H NMR (400 MHz, chloroform-*d*) δ 7.80 (s, 1H), 7.34 (d, *J* = 2.0 Hz, 1H), 6.73 (d, *J* = 2.1 Hz, 1H), 4.47 (s, 2H), 4.40 – 4.28 (m, 2H), 3.44 – 3.32 (m, 2H), 2.80 (s, 1H), 2.56 – 2.43 (m, 9H), 2.23 (s, 3H), 0.83 (m, 2H), 0.61 – 0.52 (m, 2H). ^13^C NMR (101 MHz, chloroform-*d*) δ 167.19, 150.12, 148.74, 146.79, 140.82, 135.27, 130.55, 120.80, 120.76, 116.58, 65.03, 54.55, 46.55, 22.81, 21.59 – 21.55 (m), 21.53, 21.09, 20.79, 6.78. HRMS (DART-TOF): calcd for C_21_H_27_N_4_O_2_^+^, [M + H]^+^, *m/z*, 367.2133.

#### *N*-cyclopentyl-6-methyl-4-((3,5,6-trimethylpyrazin-2-yl)methyl)-3,4-dihydro-2*H*-benzo[b][1,4]oxazine-8-carboxamide (B15)

Prepared from the general methods for **B35–38** and **B11**. ^1^H NMR (400 MHz, chloroform-*d*) δ 7.70 (d, *J* = 6.9 Hz, 1H), 7.32 (d, *J* = 2.0 Hz, 1H), 6.73 (d, *J* = 2.1 Hz, 1H), 4.47 (s, 2H), 4.37–4.29 (m, 2H), 3.40–3.33 (m, 2H), 2.57–2.45 (m, 9H), 2.23 (s, 3H), 2.12–1.98 (m, 2H), 1.77–1.58 (m, 5H), 1.56–1.42 (m, 2H). ^13^C NMR (101 MHz, chloroform-*d*) δ 165.25, 150.11, 148.76, 148.73, 146.84, 140.70, 135.27, 130.51, 121.26, 120.80, 116.37, 64.98, 54.59, 51.35, 46.56, 33.25, 23.82, 21.58–21.55 (m), 21.53, 21.12, 20.81. HRMS (DART-TOF): calcd for C_23_H_31_N_4_O_2_^+^, [M + H]^+^, *m/z*, 395.2443.

#### *N*-cyclohexyl-6-methyl-4-((3,5,6-trimethylpyrazin-2-yl)methyl)-3,4-dihydro-2*H*-benzo[b][1,4]oxazine-8-carboxamide (B16)

Prepared from the general methods for **B35–38** and **B11**. ^1^H NMR (400 MHz, chloroform-*d*) δ 7.65 (d, *J* = 7.9 Hz, 1H), 7.32 (d, *J* = 2.1 Hz, 1H), 6.73 (d, *J* = 2.1 Hz, 1H), 4.47 (s, 2H), 4.41–4.29 (m, 2H), 3.45–3.33 (m, 2H), 2.57–2.44 (m, 9H), 2.23 (s, 3H), 1.99 (m, 2H), 1.75–1.65 (m, 1H), 1.43 (m, 2H), 1.36–1.20 (m, 5H), 0.84 (s, 1H). ^13^C NMR (101 MHz, chloroform-*d*) δ 164.72, 150.11, 148.77, 146.86, 140.70, 135.27, 130.49, 121.42, 120.84, 116.33, 66.09–64.06 (m), 54.61, 46.55, 33.03, 25.78, 24.72, 21.56, 21.53, 21.10, 20.77. HRMS (DART-TOF): calcd for C_24_H_33_N_4_O_2_^+^, [M + H]^+^, *m/z*, 409.2589.

#### 6-cyclopropyl-*N*-(2,2-difluoroethyl)-4-((3,5,6-trimethylpyrazin-2-yl)methyl)-3,4-dihydro-2*H*-benzo[b][1,4]oxazine-8-carboxamide (B17)

Prepared from the general methods for **B35–37, B41,** and **B18**. ^1^H NMR (400 MHz, chloroform-*d*) δ 8.08 (t, *J* = 6.0 Hz, 1H), 7.26 (d, *J* = 6.2 Hz, 1H), 6.73 (d, *J* = 2.1 Hz, 1H), 5.95 (m, 1H), 4.48 (s, 2H), 4.39 (m, 2H), 3.80 (m, 2H), 3.45 (m, 2H), 2.67–2.38 (m, 9H), 1.78 (m, 1H), 0.87 (m, 2H), 0.60 (m, 2H). ^13^C NMR (101 MHz, chloroform-*d*) δ 166.39, 150.21, 148.73, 146.86, 141.05, 136.79, 135.53, 119.74, 117.56, 114.23, 114.22, 65.34, 55.06, 47.00, 21.58–21.54 (m), 21.53, 20.86, 15.11, 8.65. HRMS (DART-TOF): calcd for C_22_H_27_F_2_N_4_O_2_^+^, [M + H]^+^, *m/z*, 417.2094.

#### 6-cyclopropyl-*N*-(2,2,2-trifluoroethyl)-4-((3,5,6-trimethylpyrazin-2-yl)methyl)-3,4-dihydro-2*H*-benzo[b][1,4]oxazine-8-carboxamide (B18)

Prepared from the general methods for **B35–37, B41,** and **B18**. ^1^H NMR (400 MHz, chloroform-*d*) δ 8.16 (t, *J* = 6.3 Hz, 1H), 7.26 (d, *J* = 2.2 Hz, 1H), 6.74 (d, *J* = 2.2 Hz, 1H), 4.48 (s, 2H), 4.41–4.35 (m, 2H), 4.12 (m, 2H), 3.54–3.42 (m, 2H), 2.55 (s, 3H), 2.49 (d, *J* = 2.2 Hz, 6H), 1.77 (m, 1H), 0.93–0.80 (m, 2H), 0.67–0.51 (m, 2H). ^13^C NMR (101 MHz, chloroform-*d*) δ 166.09, 150.23, 148.76, 148.71, 146.82, 141.01, 136.89, 135.54, 119.49, 117.74, 114.37, 65.38, 55.02, 47.01, 41.01, 40.67, 21.54, 20.85, 15.11, 8.66. HRMS (DART-TOF): calcd for C_22_H_26_F_3_N_4_O_2_^+^, [M + H]^+^, *m/z*, 435.2003.

#### 6-cyclopropyl-*N*-(2-hydroxyethyl)-4-((3,5,6-trimethylpyrazin-2-yl)methyl)-3,4-dihydro-2*H*-benzo[b][1,4]oxazine-8-carboxamide (B19)

Prepared from the general methods for **B35–37, B41,** and **B18**. ^1^H NMR (400 MHz, chloroform-*d*) δ 8.19 (t, *J* = 5.6 Hz, 1H), 7.24 (d, *J* = 2.2 Hz, 1H), 6.72 (d, *J* = 2.2 Hz, 1H), 4.47 (s, 2H), 4.42–4.33 (m, 2H), 3.80 (t, *J* = 4.8 Hz, 2H), 3.60 (m, 2H), 3.42 (t, *J* = 4.5 Hz, 2H), 2.54 (s, 3H), 2.50 (s, 3H), 2.49 (s, 3H), 1.77 (m, 1H), 0.92–0.79 (m, 2H), 0.66–0.55 (m, 2H). ^13^C NMR (101 MHz, chloroform-*d*) δ 167.42, 150.19, 148.76, 148.73, 146.93, 140.94, 136.67, 135.46, 120.27, 117.56, 114.02, 65.28, 63.08, 55.09, 46.99, 43.16, 21.52, 20.84, 15.12, 8.64. HRMS (DART-TOF): calcd for C_22_H_29_N_4_O_3_^+^, [M + H]^+^, *m/z*, 397.2238.

#### 6-cyclopropyl-4-(naphthalen-2-ylmethyl)-*N*-(2,2,2-trifluoroethyl)-3,4-dihydro-2*H*-benzo[b][1,4]oxazine-8-carboxamide (B20)

Prepared from the general methods for **B35–37, B41,** and **B18**. ^1^H NMR (400 MHz, chloroform-*d*) δ 8.20 (t, *J* = 6.4 Hz, 1H), 7.84 (d, *J* = 8.2 Hz, 2H), 7.81–7.78 (m, 1H), 7.72 (s, 1H), 7.48 (m, 2H), 7.40 (m, 1H), 7.23 (d, *J* = 2.2 Hz, 1H), 6.68 (d, *J* = 2.2 Hz, 1H), 4.62 (s, 2H), 4.41 (t, *J* = 4.5 Hz, 2H), 4.13 (m, 2H), 3.45 (t, *J* = 4.5 Hz, 2H), 1.73 (m, 1H), 0.92–0.72 (m, 2H), 0.61–0.44 (m, 2H). ^13^C NMR (101 MHz, chloroform-*d*) δ 166.12, 140.76, 137.27, 135.65, 134.91, 133.46, 132.85, 128.73, 127.75, 127.71, 126.38, 125.94, 125.72, 125.16, 123.08, 119.73, 116.98, 114.63, 65.27, 55.80, 46.71, 41.04, 40.70, 15.12, 8.66. MS(ESI): calcd for C_25_H_24_F_3_N_2_O_2_^+^, [M + H]^+^, *m/z*, 441.2.

#### 6-cyclopropyl-4-(4-methylbenzyl)-*N*-(2,2,2-trifluoroethyl)-3,4-dihydro-2*H*-benzo[b][1,4]oxazine-8-carboxamide (B21)

Prepared from the general methods for **B35–37, B41,** and **B18**. ^1^H NMR (400 MHz, chloroform-*d*) δ 8.18 (t, *J* = 6.6 Hz, 1H), 7.22 (d, *J* = 2.2 Hz, 1H), 7.16 (s, 4H), 6.62 (d, *J* = 2.2 Hz, 1H), 4.43 (s, 2H), 4.37 (t, *J* = 4.5 Hz, 2H), 4.12 (m, 2H), 3.39 (t, *J* = 4.4 Hz, 2H), 2.35 (s, 3H), 1.75 (m, 1H), 0.83 (h, *J* = 4.6 Hz, 2H), 0.63–0.52 (m, 2H). ^13^C NMR (101 MHz, chloroform-*d*) δ 166.13, 140.68, 137.16, 137.09, 135.64, 134.28, 129.48, 127.08, 125.84, 123.07, 119.61, 116.84, 114.43, 65.24, 55.22, 46.55, 21.11, 21.08, 15.11, 8.63. HRMS (DART-TOF): calcd for C_22_H_24_F_3_N_2_O_2_^+^, [M + H]^+^, *m/z*, 405.1771.

#### 6-cyclopropyl-4-(3,5-dimethylbenzyl)-*N*-(2,2,2-trifluoroethyl)-3,4-dihydro-2*H*-benzo[b][1,4]oxazine-8-carboxamide (B22)

Prepared from the general methods for **B35–37, B41,** and **B18**. ^1^H NMR (400 MHz, chloroform-*d*) δ 8.18 (s, 1H), 7.22 (d, *J* = 2.2 Hz, 1H), 6.92 (s, 1H), 6.89 (s, 2H), 6.62 (d, *J* = 2.2 Hz, 1H), 4.38 (d, *J* = 5.3 Hz, 4H), 4.13 (m, 2H), 3.52–3.37 (m, 2H), 2.31 (s, 6H), 1.75 (m, 1H), 0.85–0.82 (m, 2H), 0.63–0.54 (m, 2H). ^13^C NMR (101 MHz, chloroform-*d*) δ 166.14, 140.69, 138.42, 137.45, 137.17, 135.75, 129.05, 124.87, 119.60, 116.82, 114.45, 65.22, 55.59, 46.67, 21.37, 21.34, 15.10, 8.62. HRMS (DART-TOF): calcd for C_23_H_26_F_3_N_2_O_2_^+^, [M + H]^+^, *m/z*, 419.1932.

#### 6-cyclopropyl-4-(3,5-dimethoxybenzyl)-*N*-(2,2,2-trifluoroethyl)-3,4-dihydro-2*H*-benzo[b][1,4]oxazine-8-carboxamide (B23)

Prepared from the general methods for **B35–37, B41,** and **B18**. ^1^H NMR (400 MHz, chloroform-*d*) δ 8.18 (t, *J* = 6.3 Hz, 1H), 7.22 (d, *J* = 2.2 Hz, 1H), 6.61 (d, *J* = 2.2 Hz, 1H), 6.43 (d, *J* = 2.2 Hz, 2H), 6.38 (t, *J* = 2.3 Hz, 1H), 4.39 (s, 4H), 4.12 (m, 2H), 3.77 (s, 6H), 3.41 (t, *J* = 4.5 Hz, 2H), 1.75 (m, 1H), 0.92–0.75 (m, 2H), 0.65–0.54 (m, 2H). ^13^C NMR (101 MHz, chloroform-*d*) δ 166.10, 161.26, 140.71, 140.12, 137.18, 135.55, 125.84, 123.07, 119.64, 117.02, 114.56, 104.99, 98.95, 65.24, 55.74, 55.37, 55.34, 46.77, 41.03, 40.68, 15.10, 8.65. HRMS (DART-TOF): calcd for C_23_H_26_F_3_N_2_O_4_^+^, [M + H]^+^, *m/z*, 451.1827.

#### 6-cyclopropyl-4-(3-fluoro-4-methoxybenzyl)-*N*-(2,2,2-trifluoroethyl)-3,4-dihydro-2*H*-benzo[b][1,4]oxazine-8-carboxamide (B24)

Prepared from the general methods for **B35–37, B41,** and **B18**. ^1^H NMR (400 MHz, chloroform-*d*) δ 8.17 (t, *J* = 6.4 Hz, 1H), 7.23 (d, *J* = 2.2 Hz, 1H), 7.10–6.89 (m, 3H), 6.58 (d, *J* = 2.2 Hz, 1H), 4.39 (d, *J* = 5.4 Hz, 4H), 4.12 (m, 2H), 3.88 (s, 3H), 3.40 (t, *J* = 4.5 Hz, 2H), 1.75 (m, 1H), 0.92–0.75 (m, 2H), 0.64–0.51 (m, 2H). ^13^C NMR (101 MHz, chloroform-*d*) δ 166.03, 153.84, 151.39, 146.96, 146.85, 140.75, 137.19, 135.35, 130.47, 130.42, 122.66, 119.73, 117.19, 114.51, 113.69, 65.22, 56.38, 56.33, 54.79, 46.71, 15.09, 8.65. MS(ESI): calcd for C_22_H_23_F_4_N_2_O_3_^+^, [M + H]^+^, *m/z*, 439.2.

#### methyl 3-((6-cyclopropyl-8-((2,2,2-trifluoroethyl)carbamoyl)-2,3-dihydro-4*H*-benzo[b][1,4]oxazin-4-yl)methyl)benzoate (B25)

Prepared from the general methods for **B35–37, B41,** and **B18**. ^1^H NMR (400 MHz, chloroform-*d*) δ 8.17 (t, *J* = 6.3 Hz, 1H), 7.96 (d, *J* = 7.5 Hz, 2H), 7.46 (m, 2H), 7.23 (s, 1H), 6.56 (s, 1H), 4.50 (s, 2H), 4.41 (t, *J* = 4.5 Hz, 2H), 4.19–4.06 (m, 2H), 3.92 (s, 3H), 3.44 (t, *J* = 4.5 Hz, 2H), 1.73 (m, 1H), 0.82 (d, *J* = 8.3 Hz, 2H), 0.56 (t, *J* = 5.5 Hz, 2H). ^13^C NMR (101 MHz, chloroform-*d*) δ 166.88, 166.03, 140.80, 138.11, 137.22, 135.32, 131.45, 130.79, 128.98, 128.72, 128.19, 119.76, 117.32, 117.30, 114.55, 114.53, 65.18, 55.51, 52.24, 46.99, 15.07, 8.63. MS(ESI): calcd for C_23_H_24_F_3_N_2_O_4_^+^, [M + H]^+^, *m/z*, 449.1701.

#### (S)-6-cyclopropyl-*N*-(1,1,1-trifluoropropan-2-yl)-4-((3,5,6-trimethylpyrazin-2-yl)methyl)-3,4-dihydro-2*H*-benzo[b][1,4]oxazine-8-carboxamide (B26)

Prepared from the general methods for **B35–37, B41,** and **B18**. ^1^H NMR (400 MHz, chloroform-*d*) δ 8.00 (d, *J* = 9.3 Hz, 1H), 7.25 (d, *J* = 2.2 Hz, 1H), 6.73 (d, *J* = 2.2 Hz, 1H), 5.02–4.87 (m, 1H), 4.48 (s, 2H), 4.38 (t, 2H), 3.45 (t, *J* = 4.5 Hz, 2H), 2.55 (s, 3H), 2.50 (s, 3H), 2.49 (s, 3H), 1.85–1.72 (m, 1H), 1.38 (d, *J* = 6.9 Hz, 3H), 0.91–0.80 (m, 2H), 0.63–0.55 (m, 2H). MS(ESI): calcd for C_23_H_28_F_3_N_4_O_2_^+^, [M + H]^+^, *m/z*, 449.2.

#### (R)-6-cyclopropyl-*N*-(1,1,1-trifluoropropan-2-yl)-4-((3,5,6-trimethylpyrazin-2-yl)methyl)-3,4-dihydro-2*H*-benzo[b][1,4]oxazine-8-carboxamide (B27)

Prepared from the general methods for **B35–37, B41,** and **B18**. ^1^H NMR (400 MHz, chloroform-*d*) δ 8.00 (s, 1H), 7.24 (d, *J* = 2.2 Hz, 1H), 6.73 (d, *J* = 2.2 Hz, 1H), 5.01–4.87 (m, 1H), 4.48 (s, 2H), 4.38 (t, *J* = 4.5 Hz, 2H), 3.45 (t, *J* = 4.4 Hz, 2H), 2.55 (s, 3H), 2.49 (s, 6H), 1.85–1.72 (m, 1H), 1.39 (d, *J* = 6.9 Hz, 3H), 0.92–0.79 (m, 2H), 0.66–0.55 (m, 2H). ^13^C NMR (101 MHz, chloroform-*d*) δ 165.44, 150.19, 148.71 (d, *J* = 3.7 Hz), 146.84, 140.95, 136.83, 135.53, 119.71, 117.59 (d, *J* = 2.2 Hz), 114.21 (d, *J* = 2.7 Hz), 65.27 (d, *J* = 3.8 Hz), 55.02, 47.01, 21.51 (d, *J* = 3.9 Hz), 20.84 (d, *J* = 4.5 Hz), 15.10, 14.65, 8.63. MS(ESI): calcd for C_23_H_28_F_3_N_4_O_2_^+^, [M + H]^+^, *m/z*, 449.2.

#### 6-cyclopropyl-*N*-(3,3,3-trifluoropropyl)-4-((3,5,6-trimethylpyrazin-2-yl)methyl)-3,4-dihydro-2*H*-benzo[b][1,4]oxazine-8-carboxamide (B28)

Prepared from the general methods for **B35–37, B41,** and **B18** ^1^H NMR (400 MHz, chloroform-*d*) δ 8.13 (t, *J* = 5.9 Hz, 1H), 7.25 (d, *J* = 2.2 Hz, 1H), 6.72 (d, *J* = 2.2 Hz, 1H), 4.48 (s, 2H), 4.37–4.32 (m, 2H), 3.70 (t, *J* = 6.3 Hz, 2H), 3.48–3.39 (m, 2H), 2.54 (s, 3H), 2.49 (s, 3H), 2.49 (s, 3H), 2.53–2.38 (m, 2H), 1.82–1.72 (m, 1H), 0.91–0.82 (m, 2H), 0.63–0.55 (m, 2H). ^13^C NMR (101 MHz, chloroform-*d*) δ 165.90, 150.18, 148.75, 148.71, 146.91, 140.96, 136.70, 135.48, 120.19, 117.45, 113.95 (d, *J* = 2.6 Hz), 65.15, 55.09, 47.02, 33.32–32.91 (m), 21.84–21.23 (m), 20.88, 15.12, 8.64. MS(ESI): calcd for C_23_H_28_F_3_N_4_O^+^, [M + H]^+^, *m/z*, 449.2.

#### 6-cyclopropyl-*N*-(2,2-difluoropropyl)-4-((3,5,6-trimethylpyrazin-2-yl)methyl)-3,4-dihydro-2*H*-benzo[b][1,4]oxazine-8-carboxamide (B29)

Prepared from the general methods for **B35–37, B41,** and **B18**. ^1^H NMR (400 MHz, chloroform-*d*) δ 8.09 (t, *J* = 6.2 Hz, 1H), 7.25 (d, *J* = 2.2 Hz, 1H), 6.73 (d, *J* = 2.2 Hz, 1H), 4.48 (s, 2H), 4.38 (t, *J* = 4.4 Hz, 2H), 3.98–3.76 (m, 2H), 3.44 (t, *J* = 4.4 Hz, 2H), 2.55 (s, 3H), 2.49 (s, 6H), 1.84–1.72 (m, 1H), 1.64 (t, *J* = 18.7 Hz, 3H), 0.91–0.80 (m, 2H), 0.65–0.53 (m, 2H). ^13^C NMR (101 MHz, chloroform-*d*) δ 166.12, 150.19, 148.72, 146.89, 140.97, 136.73, 135.53, 120.03, 117.64, 114.07, 65.30, 55.12, 55.09, 55.06, 47.02, 21.56, 21.40, 21.13, 20.83, 15.11, 8.64. MS(ESI): calcd for C_23_H_29_F_2_N_4_O_2_^+^, [M + H]^+^, *m/z*, 447.2.

#### 6-cyclopropyl-*N*-(2,2-difluoro-3-hydroxypropyl)-4-((3,5,6-trimethylpyrazin-2-yl)methyl)-3,4-dihydro-2*H*-benzo[b][1,4]oxazine-8-carboxamide (B30)

Prepared from the general methods for **B35–37, B41,** and **B18**. ^1^H NMR (400 MHz, chloroform-*d*) δ 8.33 (t, *J* = 6.6 Hz, 1H), 7.24 (d, *J* = 2.2 Hz, 1H), 6.75 (d, *J* = 2.2 Hz, 1H), 4.80 (t, *J* = 7.8 Hz, 1H), 4.49 (s, 2H), 4.41 (t, *J* = 4.4 Hz, 2H), 3.94–3.80 (m, 2H), 3.73–3.56 (m, 2H), 3.52–3.43 (m, 2H), 2.55 (s, 3H), 2.49 (d, *J* = 2.3 Hz, 6H), 1.84–1.69 (m, 1H), 0.96–0.79 (m, 2H), 0.65–0.51 (m, 2H). ^13^C NMR (101 MHz, chloroform-*d*) δ 168.16, 150.26, 148.79, 148.67, 146.73, 141.24, 136.96, 135.64, 118.79, 117.54, 114.58, 65.50, 61.03, 54.97, 46.99, 41.02, 21.55, 20.80, 15.09, 8.68. MS(ESI): calcd for C_23_H_29_F_2_N_4_O_3_^+^, [M + H]^+^, *m/z*, 431.2.

#### 6-cyclopropyl-*N*-(4,4,4-trifluorobutyl)-4-((3,5,6-trimethylpyrazin-2-yl)methyl)-3,4-dihydro-2*H*-benzo[b][1,4]oxazine-8-carboxamide (B31)

Prepared from the general methods for **B35–37, B41,** and **B18**. ^1^H NMR (400 MHz, chloroform-*d*) δ 7.83 (t, *J* = 6.0 Hz, 1H), 7.24 (d, *J* = 2.1 Hz, 1H), 6.72 (d, *J* = 2.2 Hz, 1H), 4.48 (s, 2H), 4.36 (t, *J* = 4.4 Hz, 2H), 3.58–3.47 (m, 2H), 3.43 (t, *J* = 4.4 Hz, 2H), 2.80 (s, 2H), 2.55 (s, 3H), 2.49 (s, 6H), 2.30–2.09 (m, 2H), 1.81–1.70 (m, 1H), 0.91–0.79 (m, 2H), 0.65–0.52 (m, 2H). ^13^C NMR (101 MHz, chloroform-*d*) δ 166.03, 150.18, 148.73, 146.93, 140.78, 136.72, 135.46, 120.61, 117.52, 113.83, 65.22, 55.10, 47.02, 38.60, 38.32, 22.49, 21.67, 20.86, 15.12, 8.63. MS(ESI): calcd for C_24_H_30_F_3_N_4_O_2_^+^, [M + H]^+^, *m/z*, 463.2.

#### 6-cyclopropyl-*N*-(4,4-difluorocyclohexyl)-4-((3,5,6-trimethylpyrazin-2-yl)methyl)-3,4-dihydro-2*H*-benzo[b][1,4]oxazine-8-carboxamide (B32)

Prepared from the general methods for **B35–37, B41,** and **B18**. ^1^H NMR (400 MHz, chloroform-*d*) δ 7.72 (d, *J* = 7.7 Hz, 1H), 7.23 (d, *J* = 2.1 Hz, 1H), 6.71 (d, *J* = 2.2 Hz, 1H), 4.48 (s, 2H), 4.35 (t, *J* = 4.4 Hz, 2H), 3.43 (t, *J* = 4.4 Hz, 2H), 2.54 (s, 3H), 2.49 (d, *J* = 1.8 Hz, 6H), 2.14–2.01 (m, 4H), 2.01–1.82 (m, 1H), 1.82–1.72 (m, 2H), 1.71–1.54 (m, 2H), 0.94–0.78 (m, 2H), 0.68–0.48 (m, 2H). ^13^C NMR (101 MHz, chloroform-*d*) δ 165.11, 150.19, 148.76, 146.93, 140.73, 136.74, 135.45, 120.74, 117.51, 113.78, 65.22, 55.10, 47.01, 46.02, 32.31, 32.06, 31.82, 28.58, 21.59, 20.89, 15.13, 8.63. MS(ESI): calcd for C_26_H_33_F_2_N_4_O_2_^+^, [M + H]^+^, *m/z*, 471.2.

#### 6-cyclopropyl-*N*-methyl-*n*-(2,2,2-trifluoroethyl)-4-((3,5,6-trimethylpyrazin-2-yl)methyl)-3,4-dihydro-2*H*-benzo[b][1,4]oxazine-8-carboxamide (B34)

Prepared from the general methods for **B35–37**, **B41,** and **B18**. ^1^H NMR (400 MHz, chloroform-*d*) δ 6.65–6.56 (m, 1H), 6.36–6.27 (m, 1H), 4.46 (s, 2H), 4.24 (s, 3H), 4.01–3.61 (m, 1H), 3.39–3.31 (m, 2H), 3.18 (s, 1H), 3.00 (s, 2H), 2.54 (s, 3H), 2.49 (d, *J* = 3.0 Hz, 6H), 1.81–1.67 (m, 1H), 0.92–0.78 (m, 2H), 0.60–0.49 (m, 2H). ^13^C NMR (101 MHz, chloroform-*d*) δ 170.76, 150.10, 148.87, 148.60, 147.06, 138.02, 137.08, 135.43, 123.54, 123.02, 113.59, 111.28, 64.86, 54.76, 47.28, 37.27, 34.13, 21.55, 21.49, 20.88, 20.83, 15.12, 8.55. MS(ESI): calcd for C_23_H_28_F_3_N_4_O_2_^+^, [M + H]^+^, *m/z*, 449.2.

## SUPPLEMENTAL MATERIAL

Supplemental information spectrum.04721-22-s0001.pdf Fig. S1 and S2; Table S1.

ASM does not own the copyrights to Supplemental Material that may be linked to, or accessed through, an article. The authors have granted ASM a non-exclusive, world-wide license to publish the Supplemental Material files. Please contact the corresponding author directly for reuse.
