# Supplementary material for: Discovery of novel reversible inhibitor of DprE1 based on benzomorpholine for the treatment of tuberculosis
Source: Microbiol Spectr. 2023 Sep 12;11(5):e04721-22. doi: 10.1128/spectrum.04721-22 (PMC10581193; doi:10.1128/spectrum.04721-22)
Supplement: Supplemental information — Fig. S1 and S2; Table S1. [file spectrum.04721-22-s0001.pdf]

Supplement information

Discovery of Novel Reversible Inhibitor of DprE1 Based on Benzomorpholine for the Treatment of Tuberculosis

Wang Xiang[1,a], Hualong He[1,a], Xianjie Duan[1,a], Zhiqun He[d], Xinyue Xu[d], Mengya Liao[e], Fei Teng[a], Xiao Li[a], Tianwen Luo[a], Jumei Zeng*[d], Luoting Yu*[a] and Chao Gao*[b,c]

a. State Key Laboratory of Biotherapy/Collaborative Innovation Center for Biotherapy, West China Hospital, West China Medical School, Sichuan University, Chengdu, Sichuan 610041, China

b. Laboratory of Human Diseases and Immunotherapies, West China Hospital, Sichuan University, Chengdu, 610041, China

c. Institute of Immunology and Inflammation, Frontiers Science Center for Disease-related Molecular Network, West China Hospital, Sichuan University, Chengdu 610041, China

d. West China School of Public Health and West China Fourth Hospital, Sichuan University, Chengdu, Sichuan, 610041, PR China.

e. Center of Gerontology and Geriatrics, West China Hospital, Sichuan University, Chengdu, China

[1]These authors contributed equally to this work

*Corresponding Authors

Table of Contents

(1) Figure S1. Compounds reported related to TBA-7371

(2) Figure S2. Inhibition rate of **B18** in H37Ra

(3) Table S1. Drug resistance list of clinical isolates

(4) ^{1}H NMR and ^{13}C NMR of representative compounds

(5) HPLC for purity determination of representative compounds

(1) Figure S1. Compounds reported related to TBA-7371

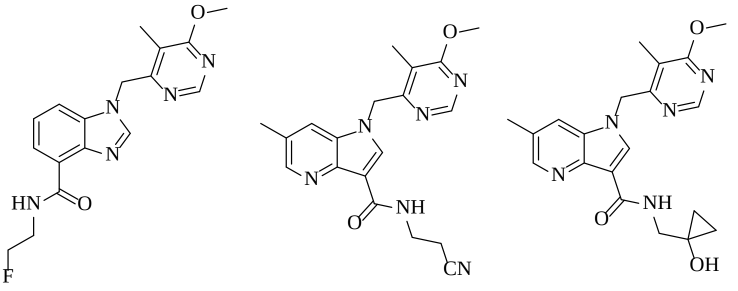

1	2	3
**Mtb MIC = 1.56 μM**<br>**DprE1 IC_{50} = 0.034 μM**	**Mtb MIC = 0.1953 μg/ml**	**Mtb MIC = 0.0625 μM**

(2) Figure S2. Inhibition rate of **B18** in H37Ra

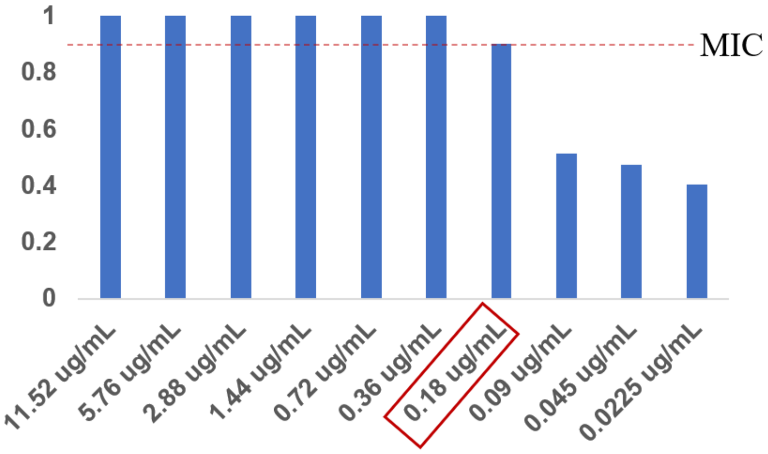

(3) Table S1. Drug resistance list of clinical isolates

strains / H37Rv	SM	INH	RMP	EMB	MOX	PA	RFB	RFT	PZA
Y17	ND	R	R	ND	ND	ND	ND	ND	ND
Y48	ND	R	R	R	S	S	ND	ND	ND
Y198	R	R	R	R	R	R	R	R	R

SM, streptomycin; INH, isoniazid: RMP rifampicin; EMB, ethambutol; MOX, moxifloxacin; PA, *p*-aminosalicylic acid; RFB, rifabutin; RFT, rifapentine; PZA, pyrazinamide. R, resistance; S, sensitivity. ND = not determined.

(4) ^{1}H NMR and ^{13}C NMR of representative compounds

Compound **A1**

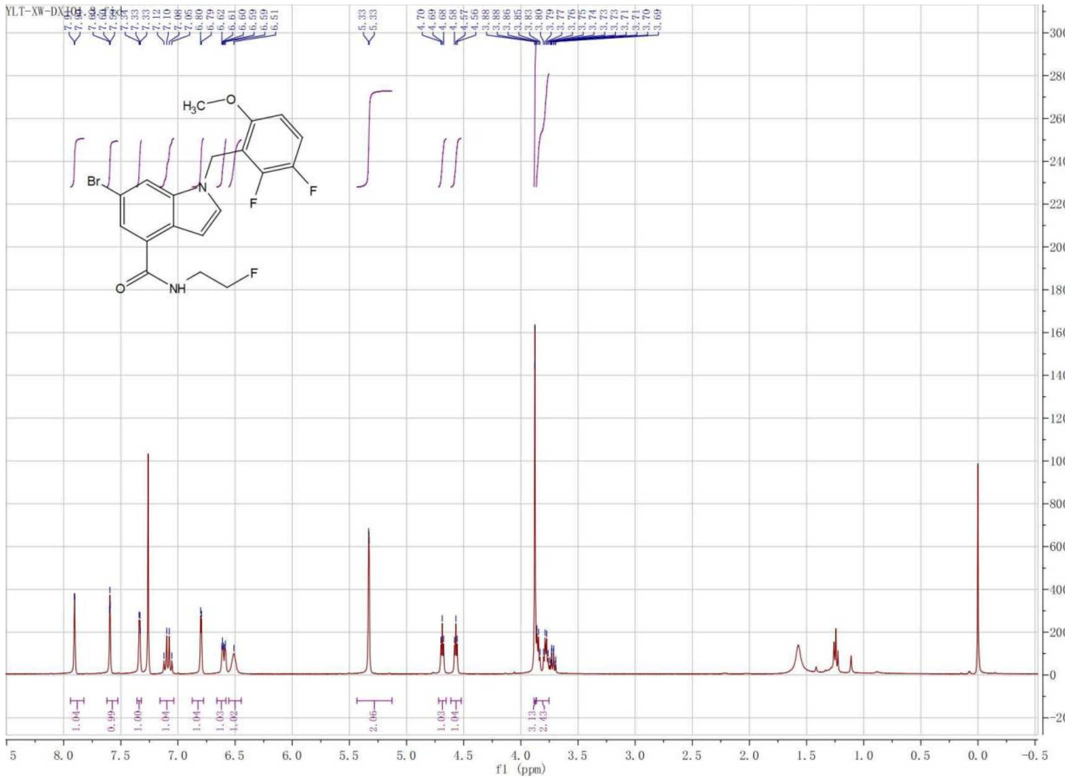

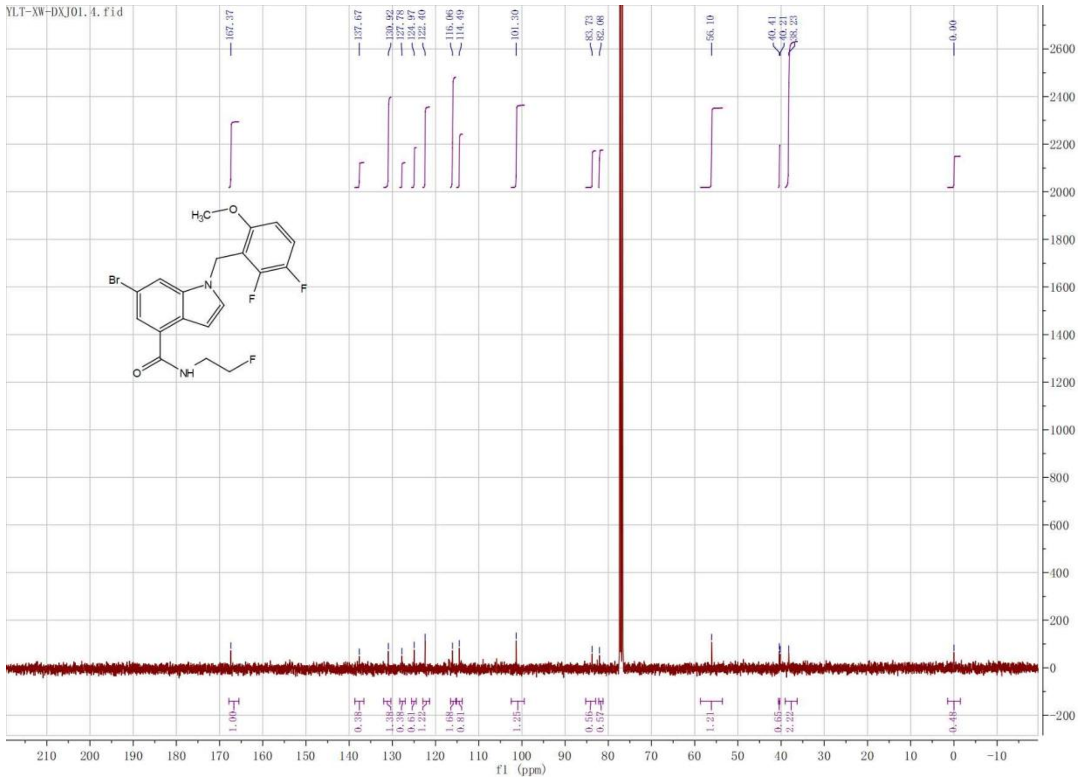

Compound **A2**

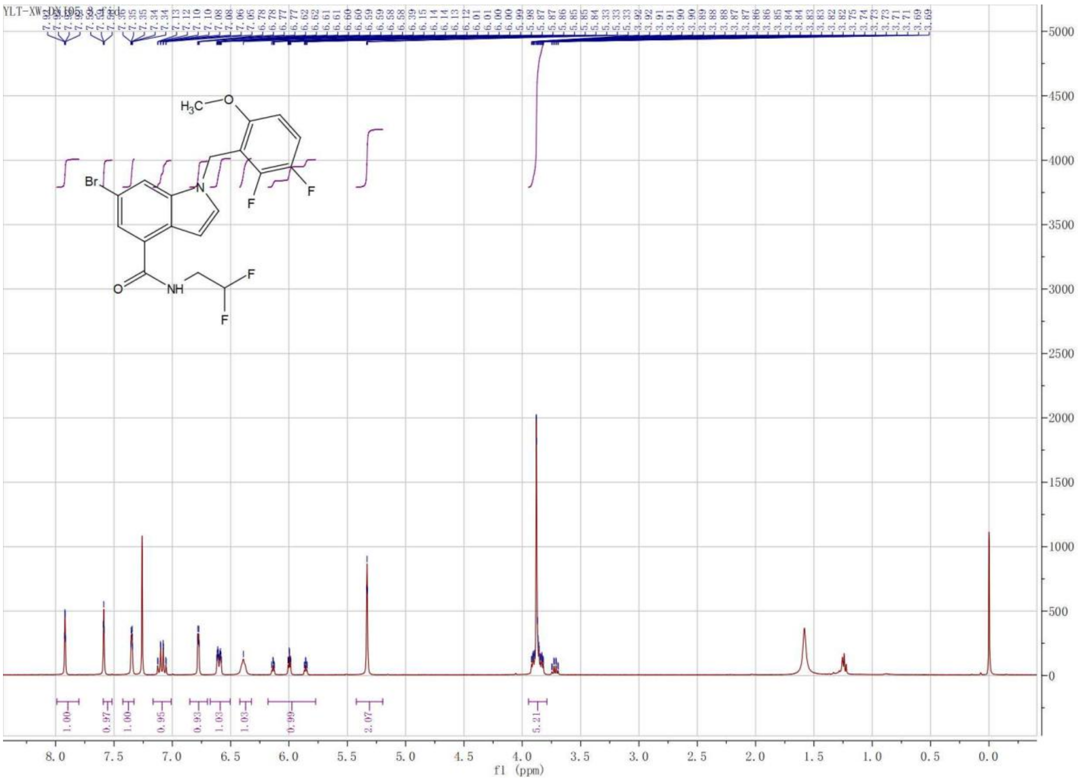

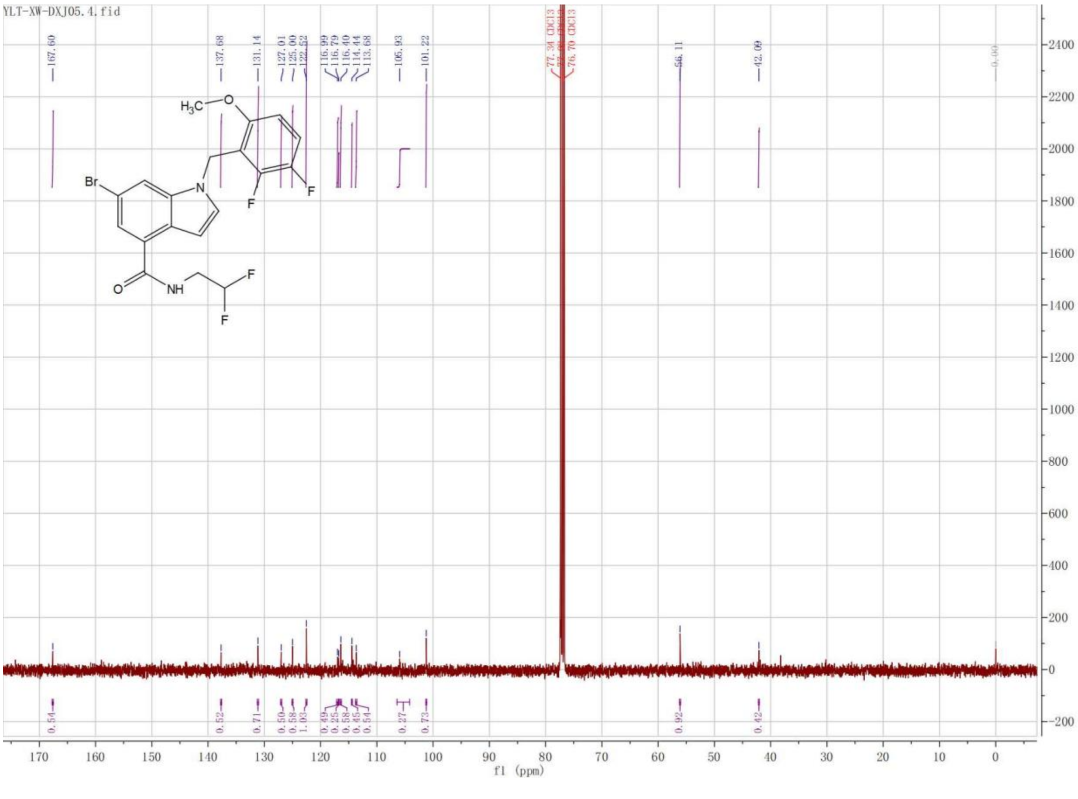

Compound **A4**

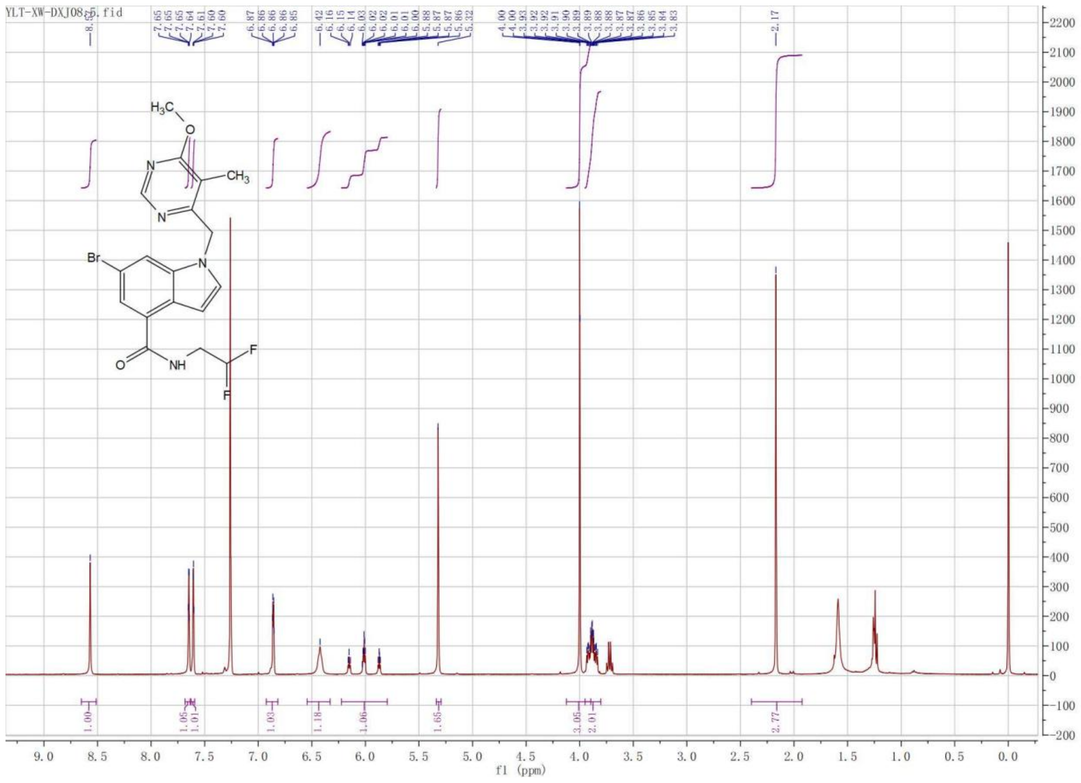

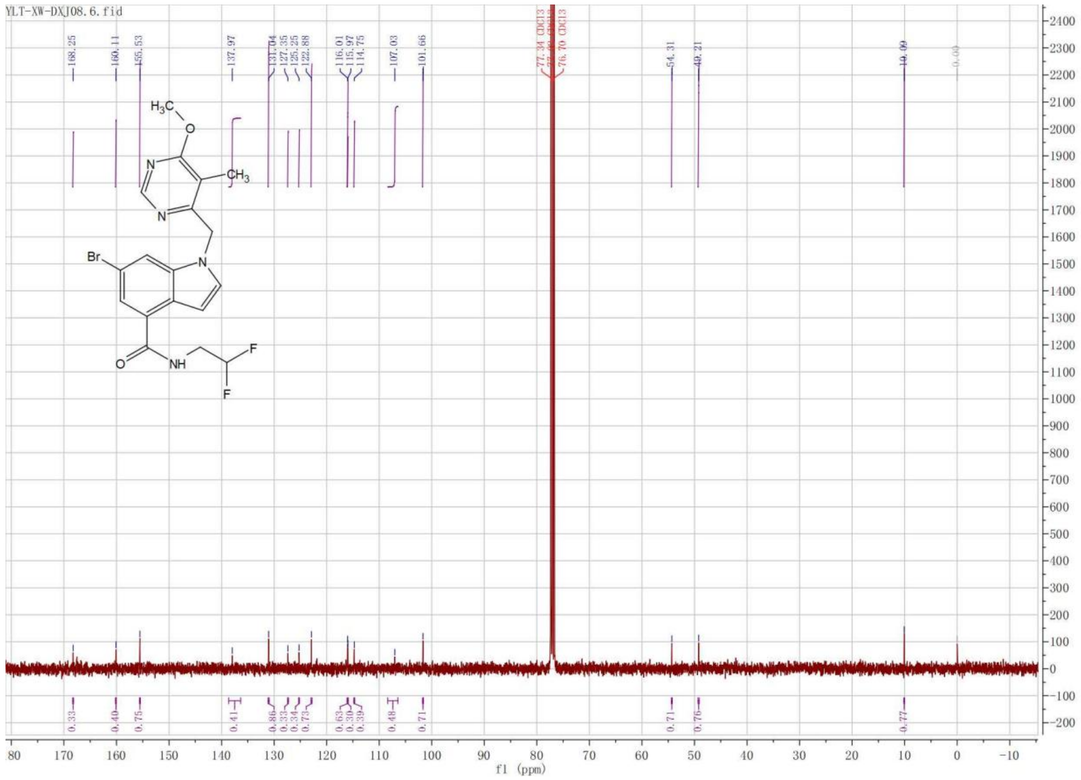

Compound **A6**

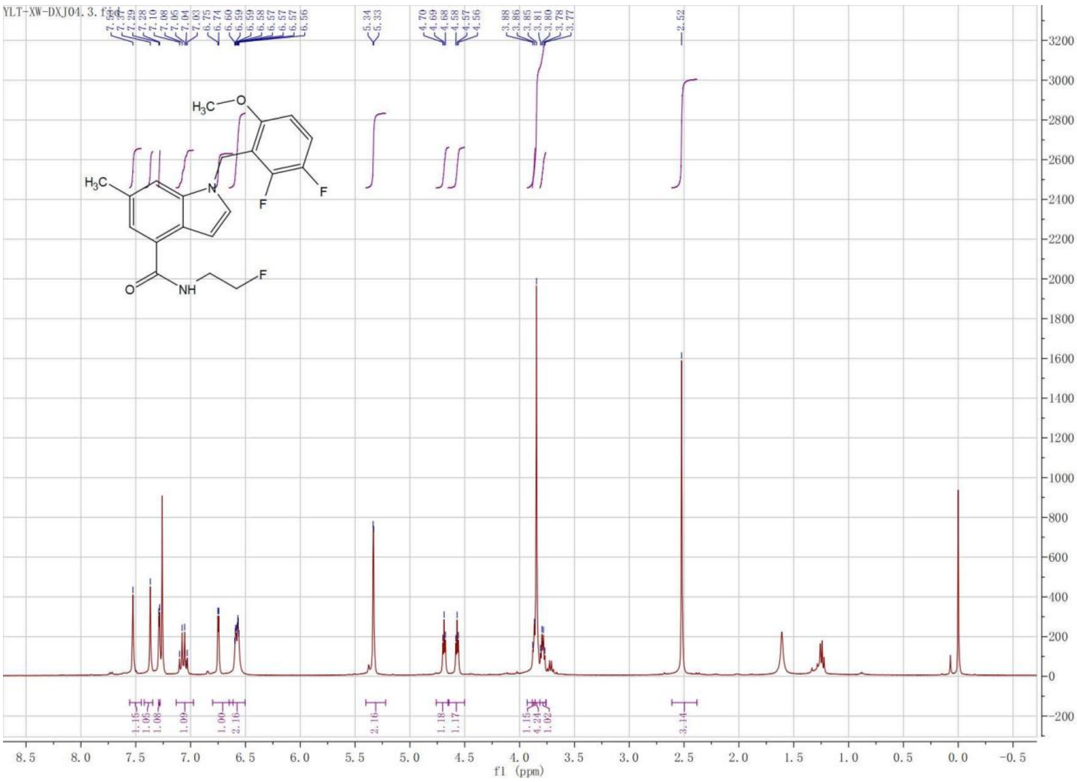

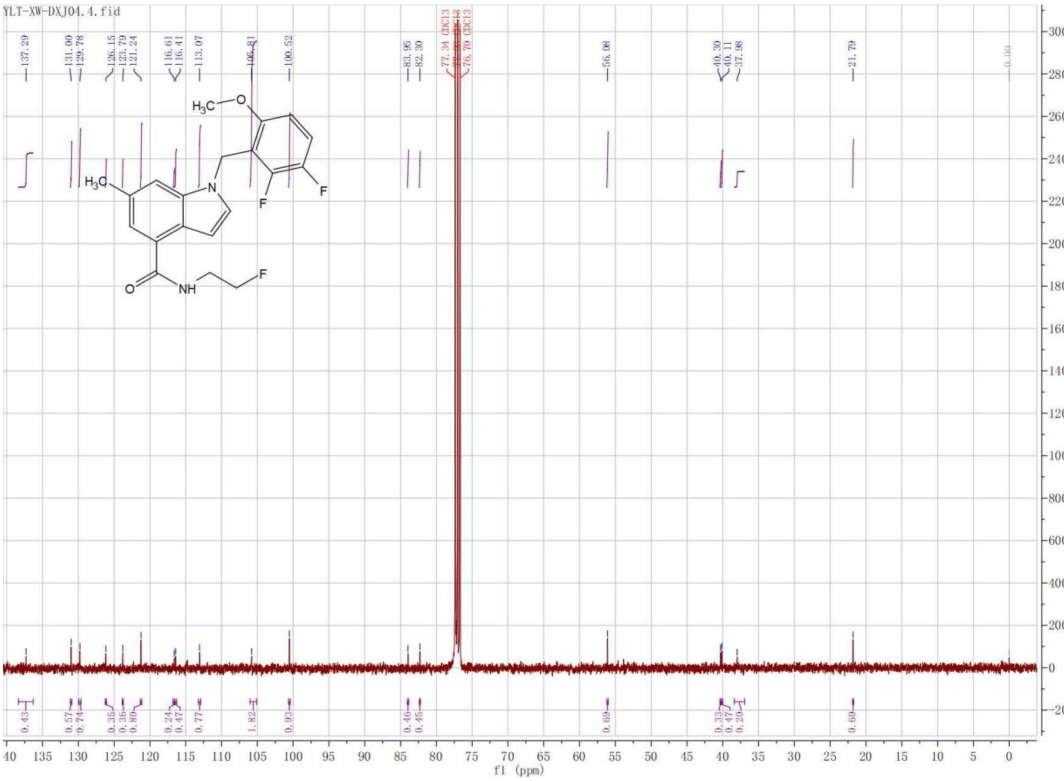

Compound **A9**

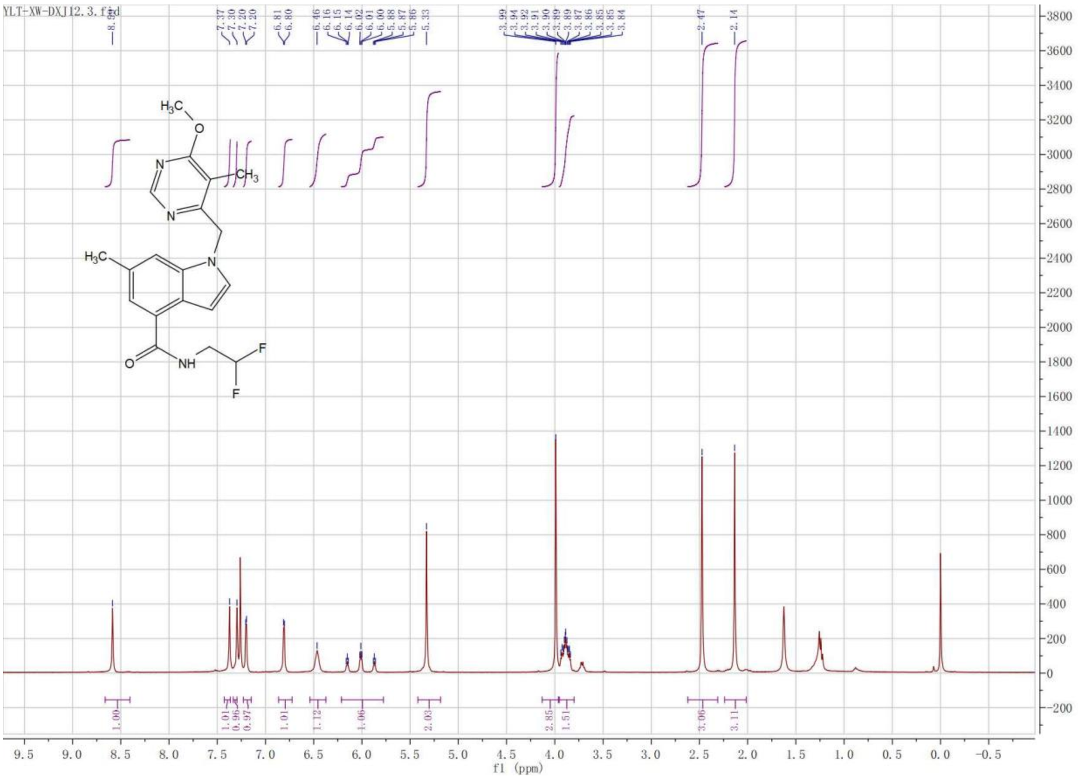

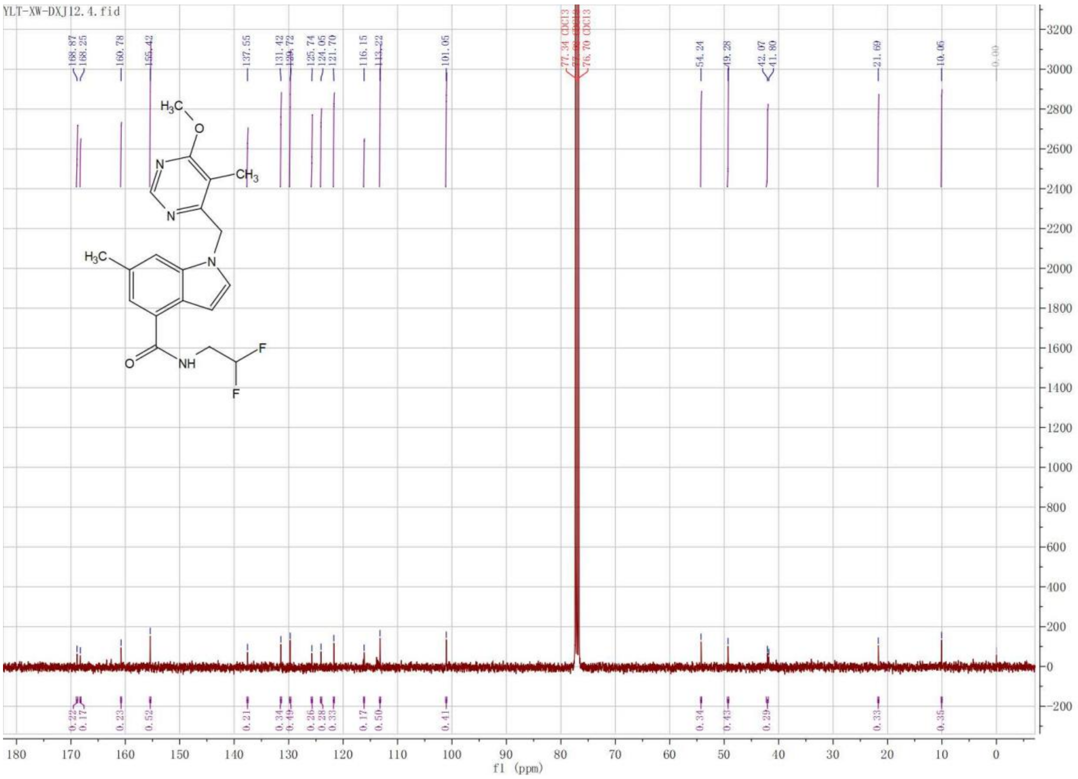

Compound **A10**

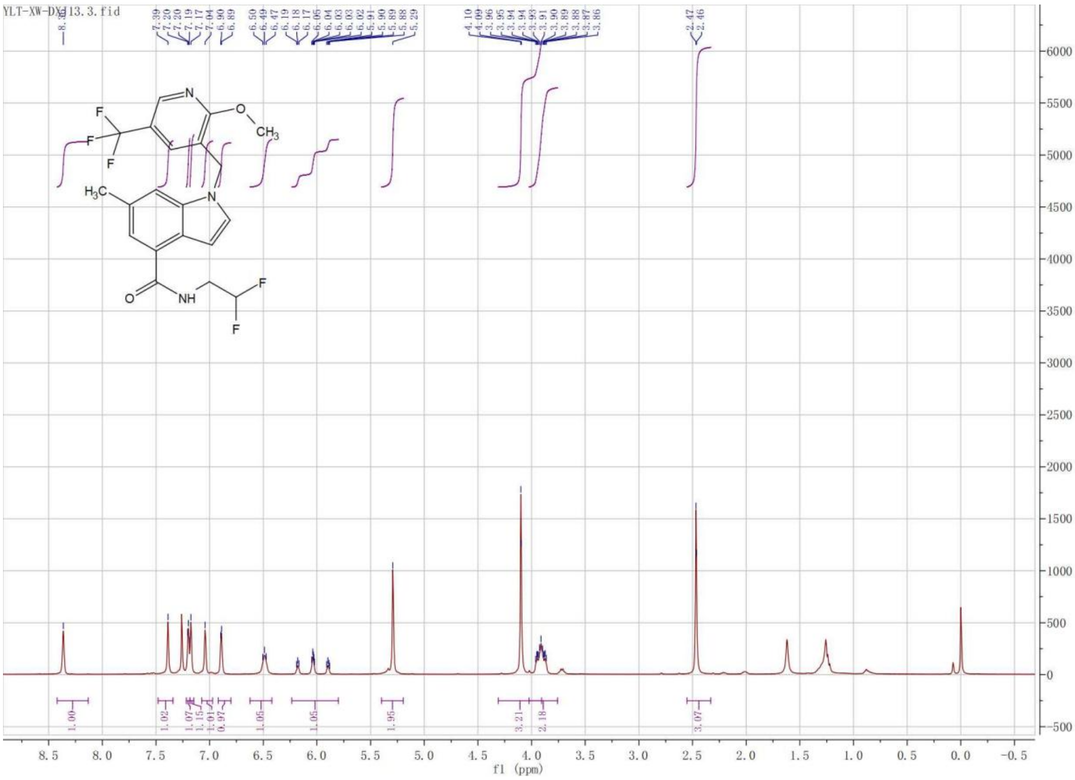

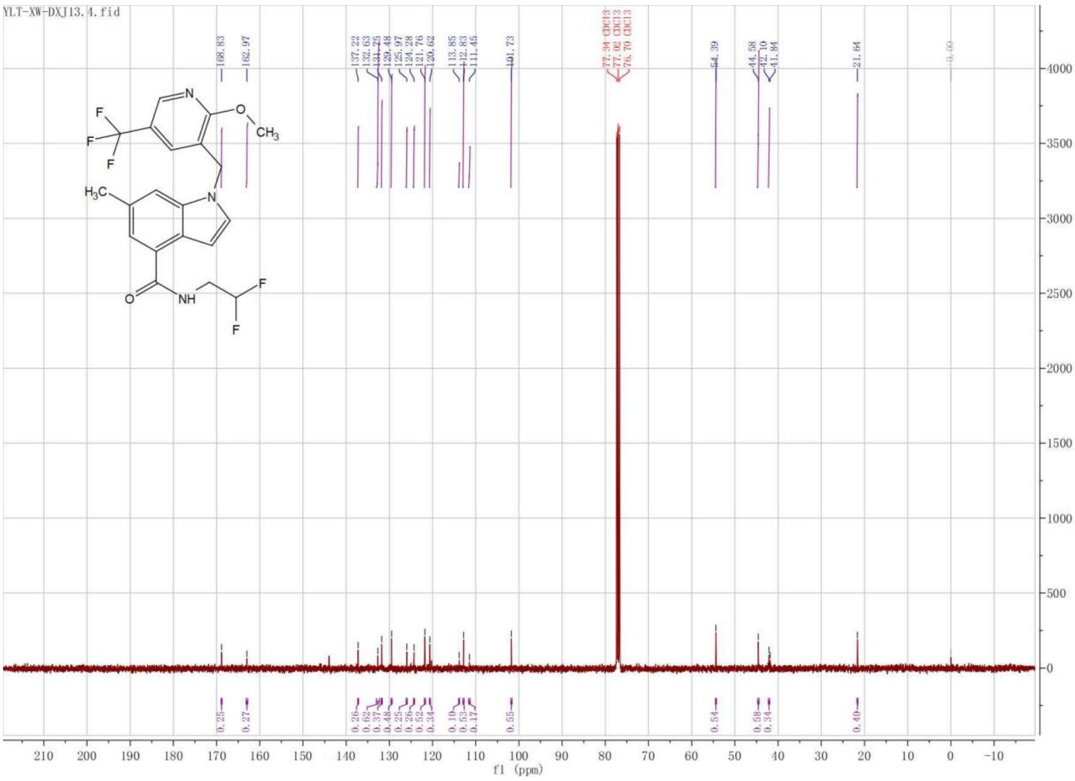

Compound **B2**

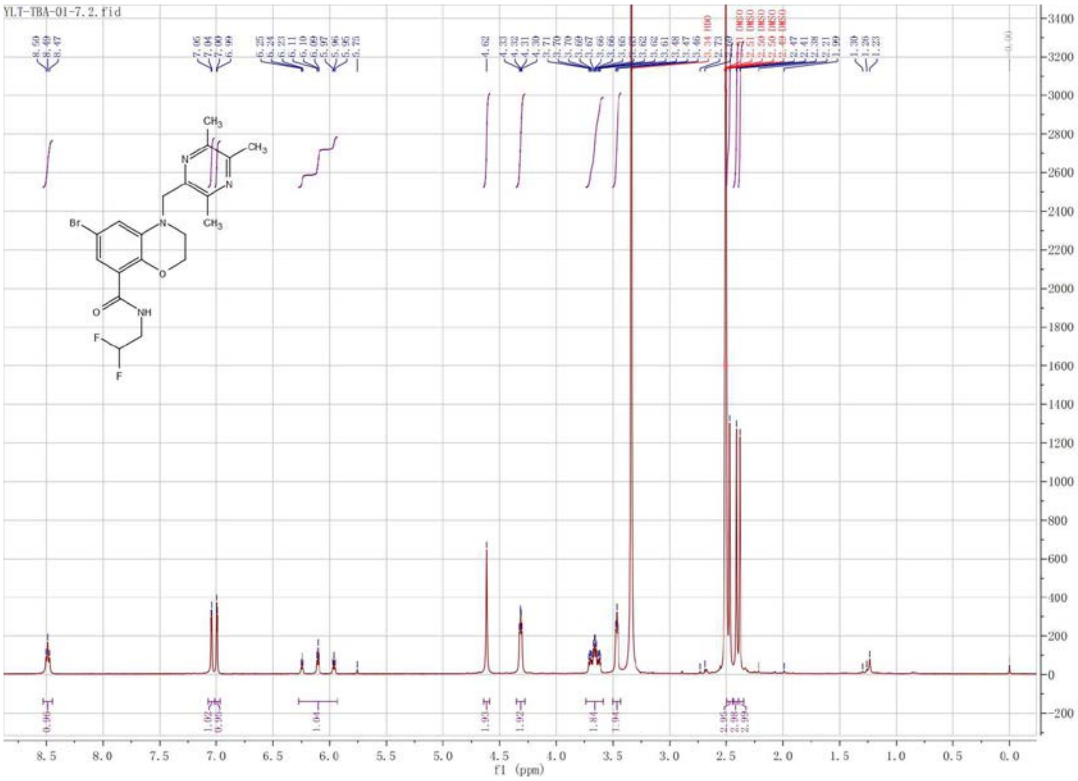

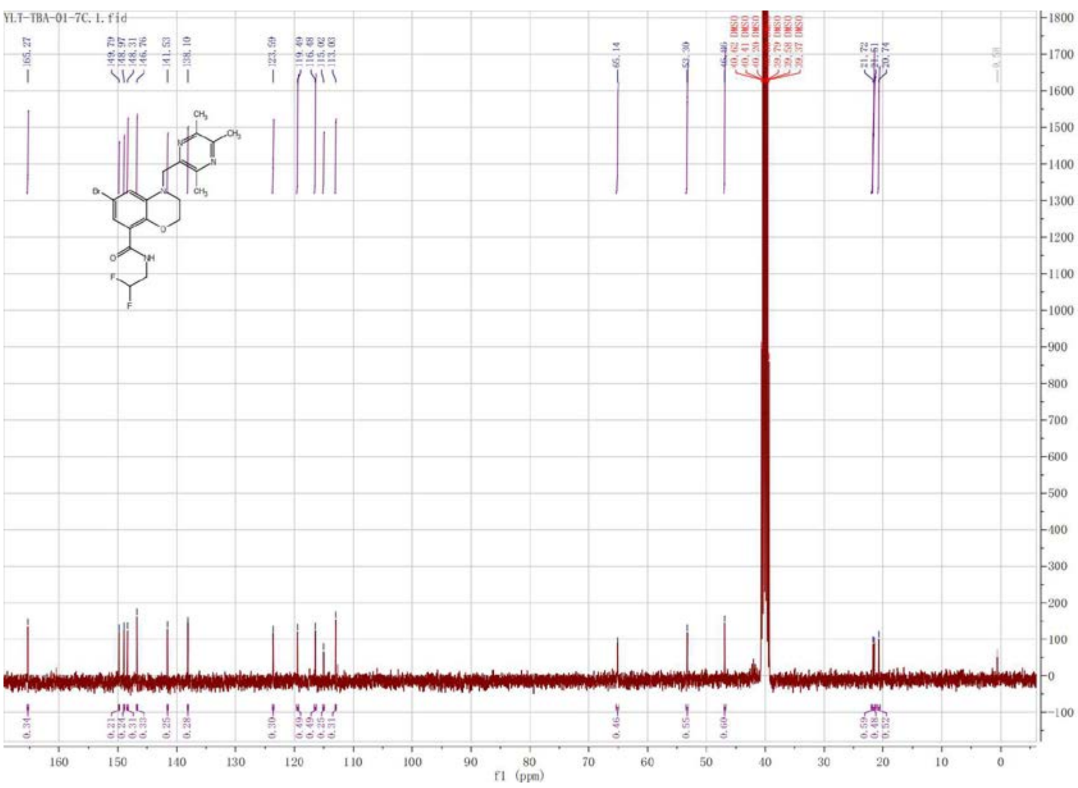
YLT-TBA-01-7C.1.fid
f1 (ppm)

Compound **B4**

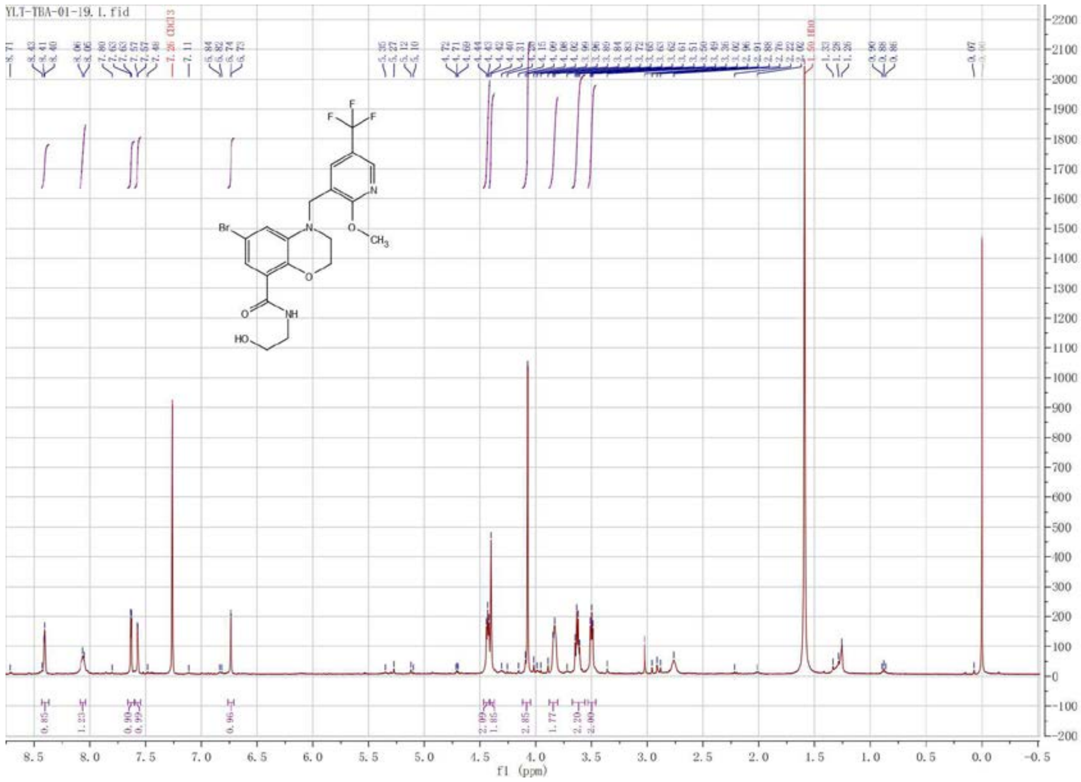

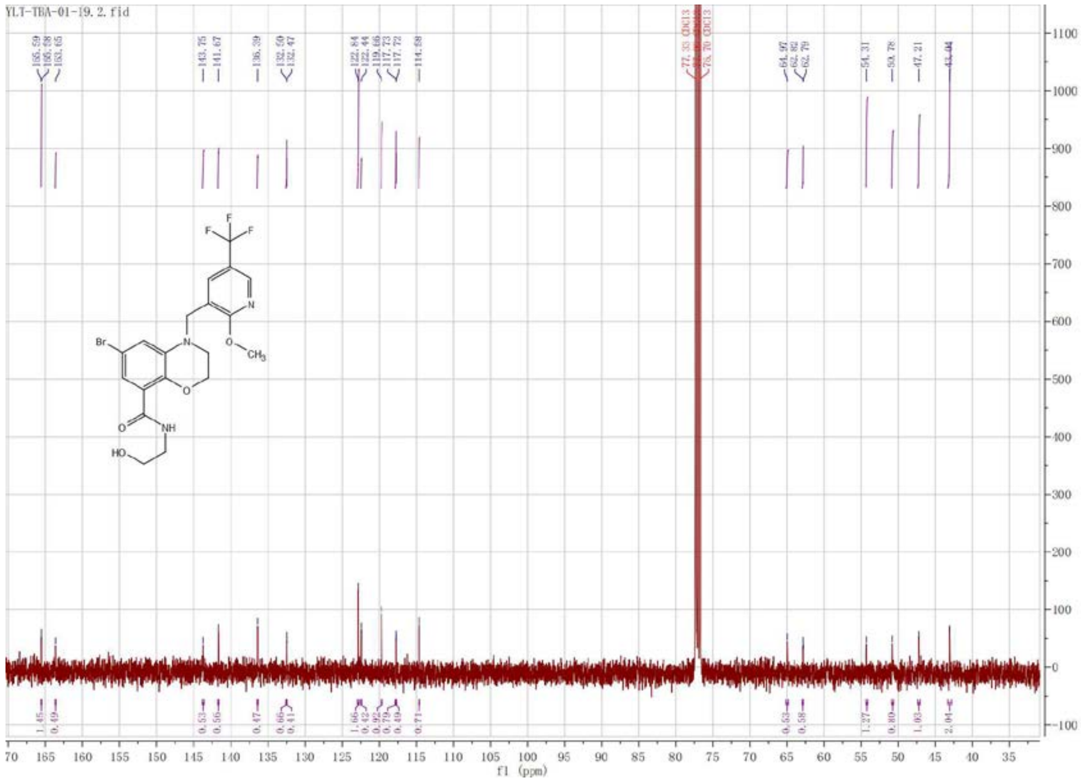

Compound **B11**

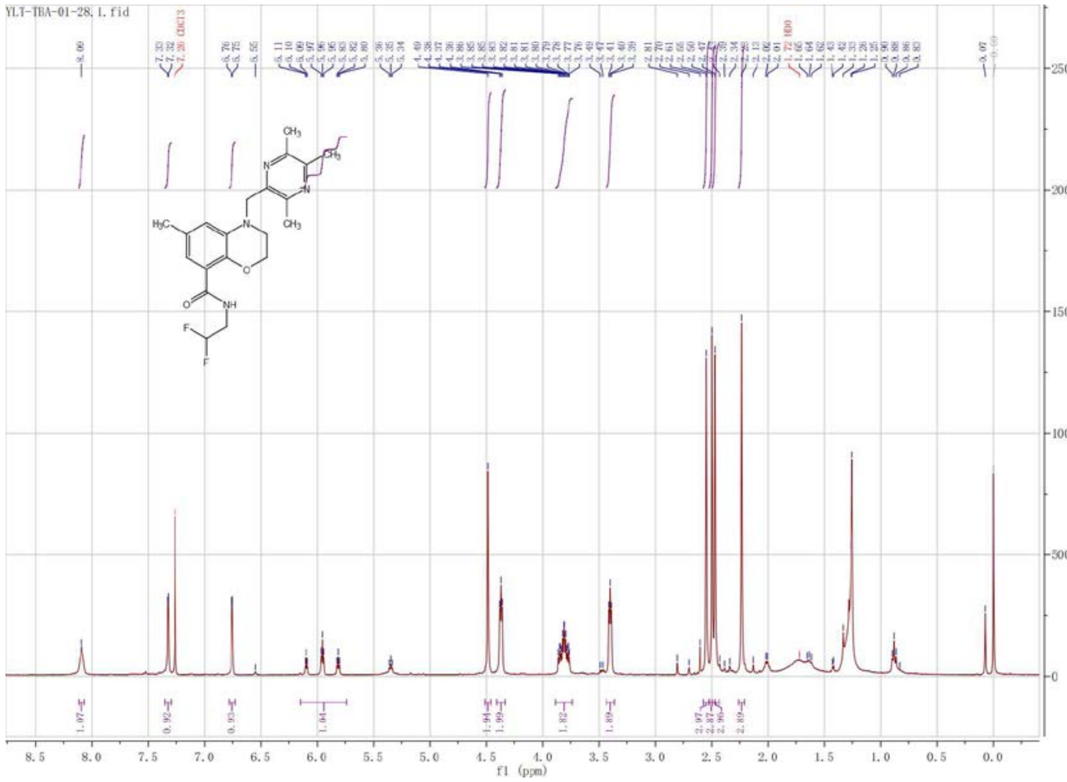

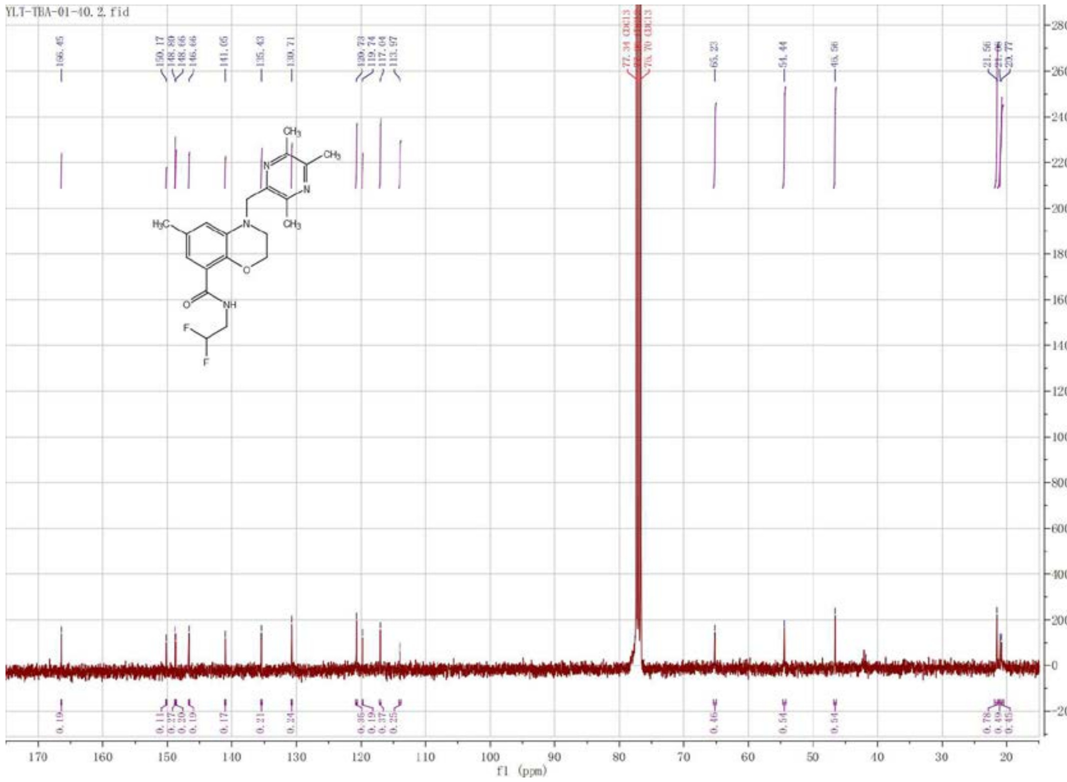

Compound **B19**

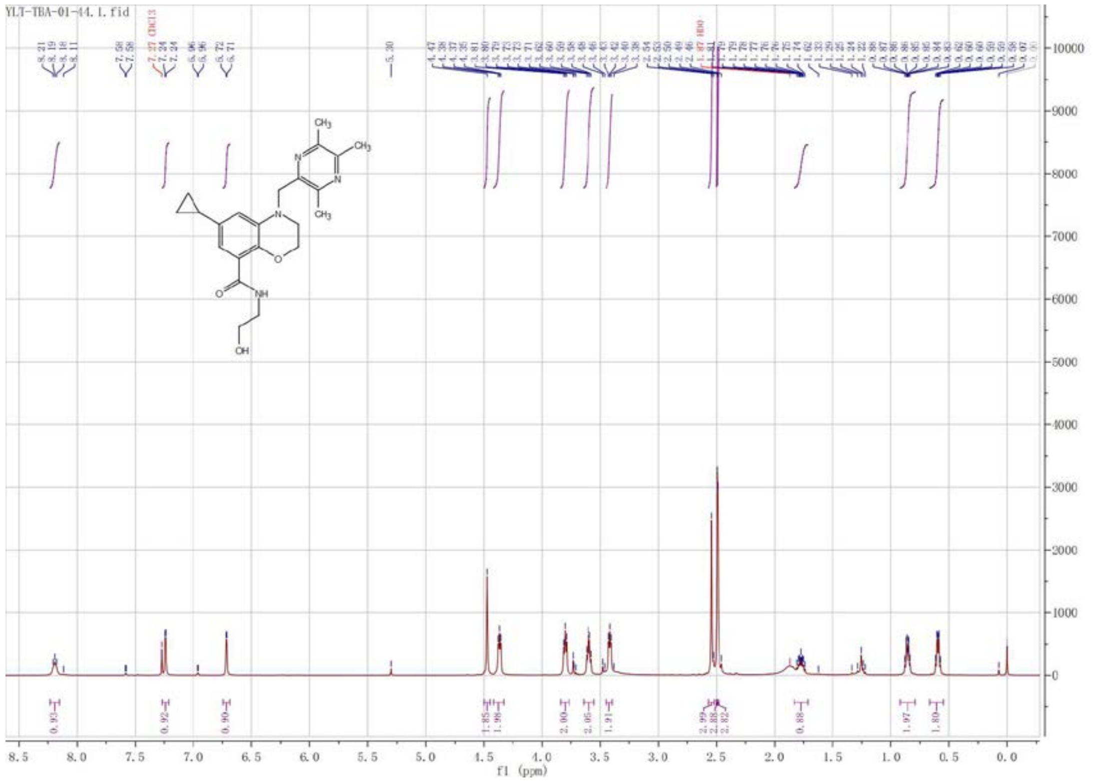

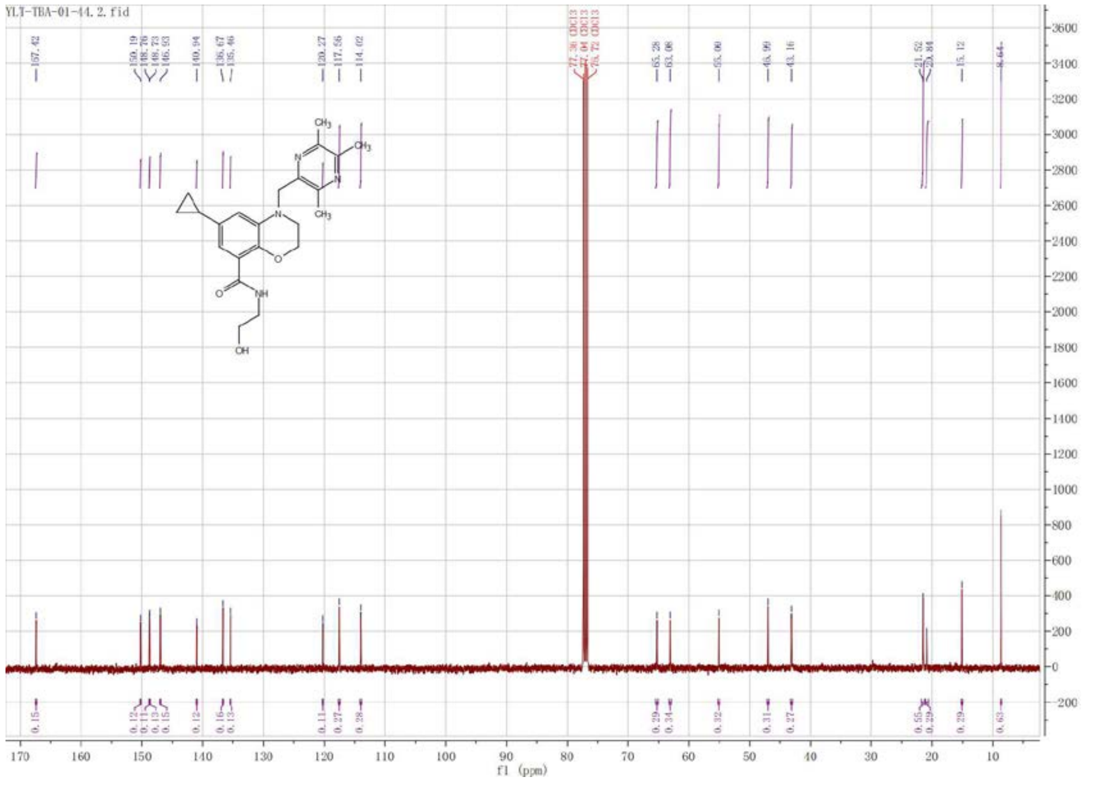

Compound **B18**

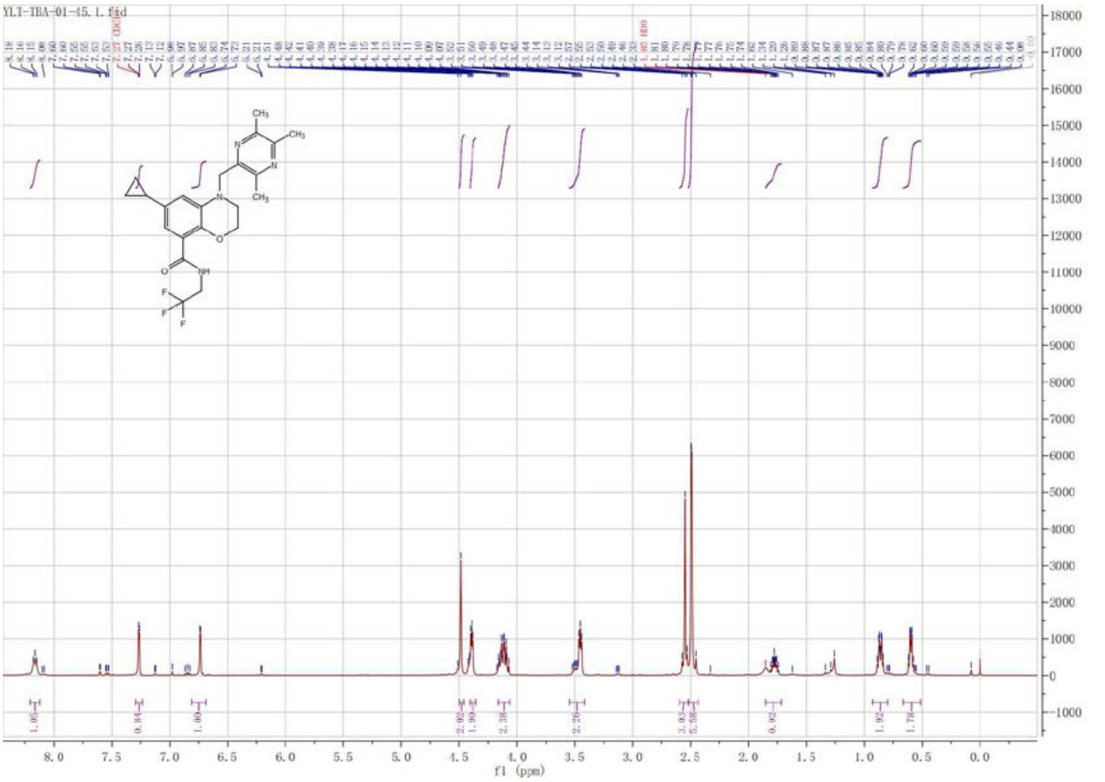

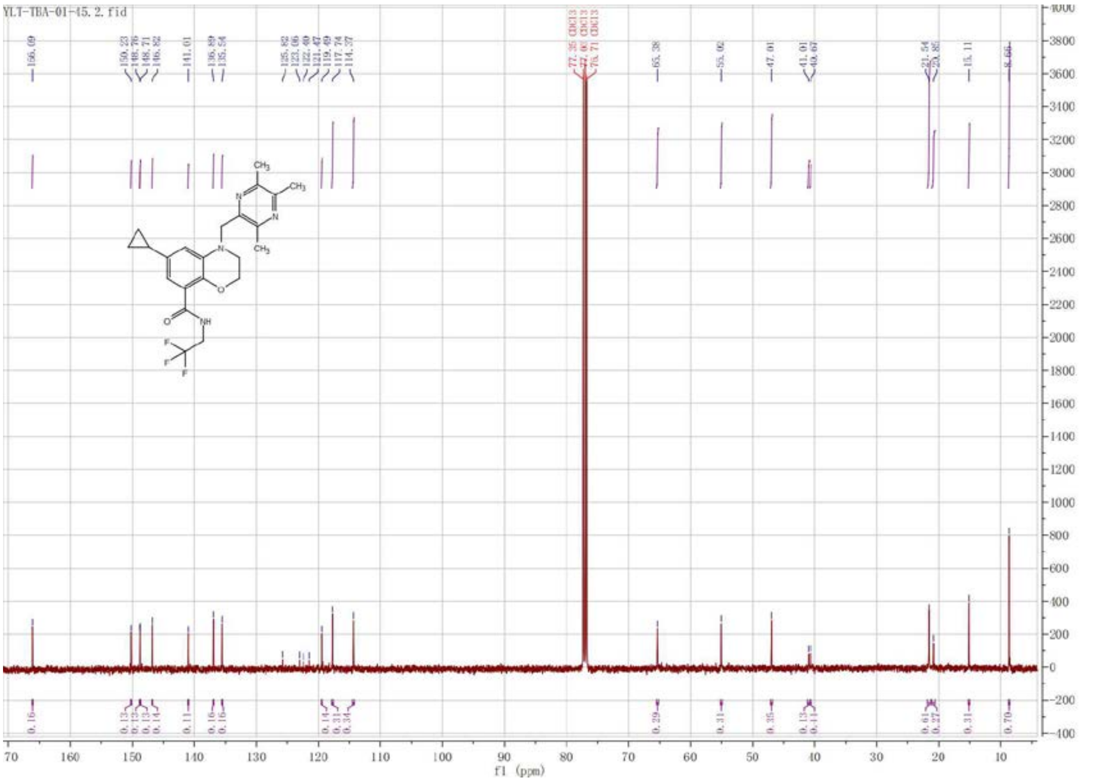

Compound **B15**

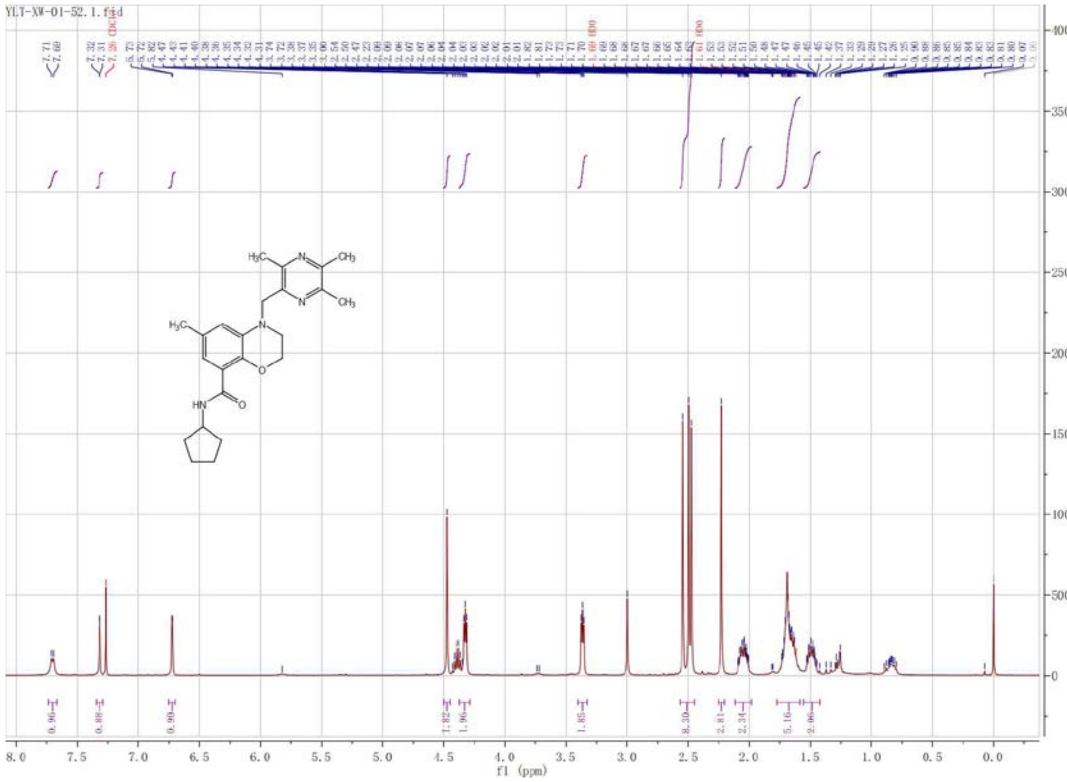

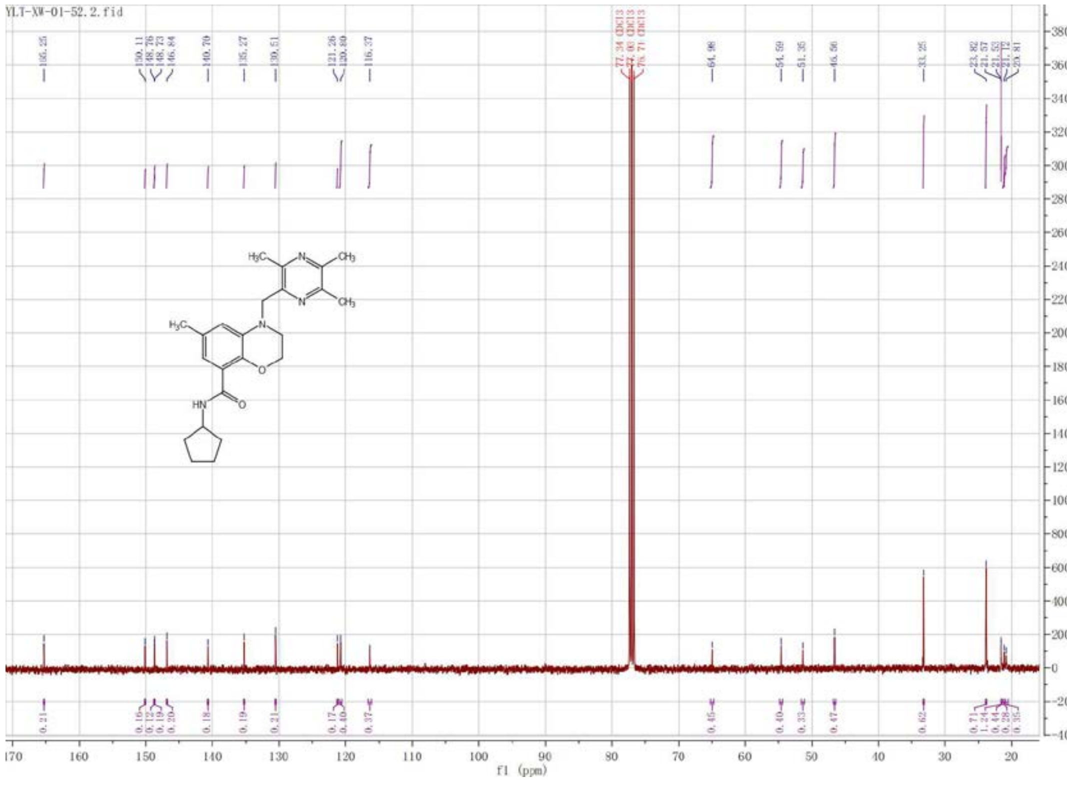

Compound **B22**

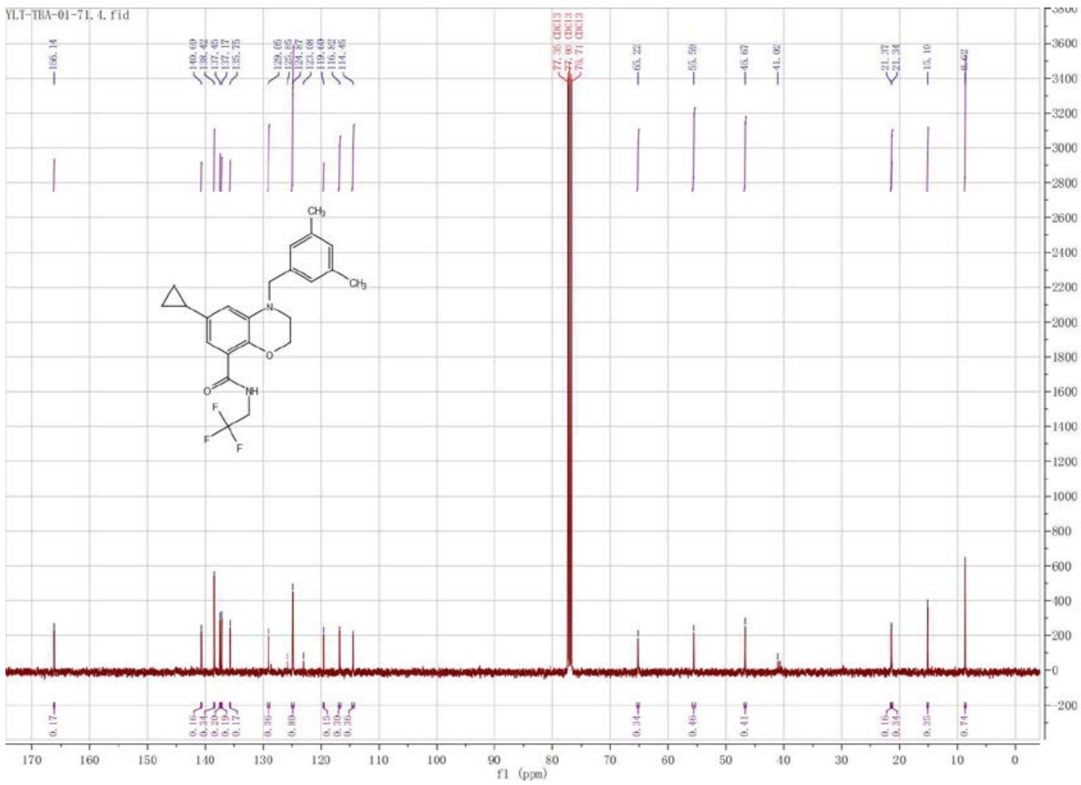

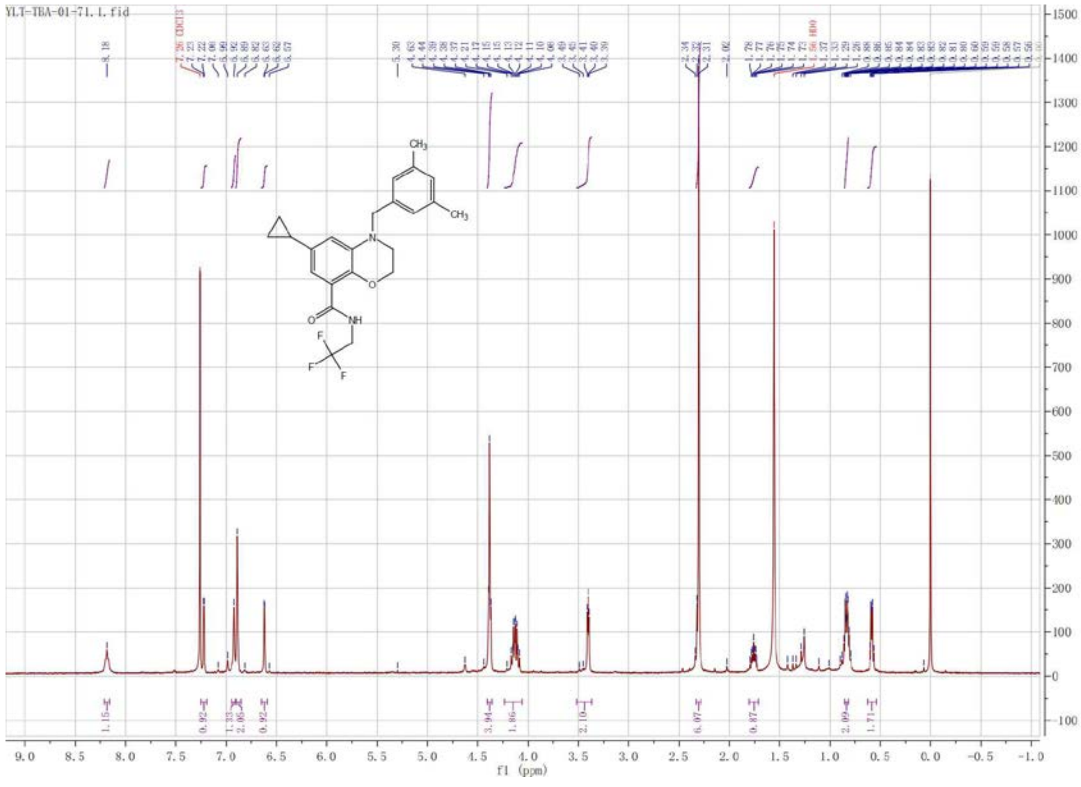

Compound **B23**

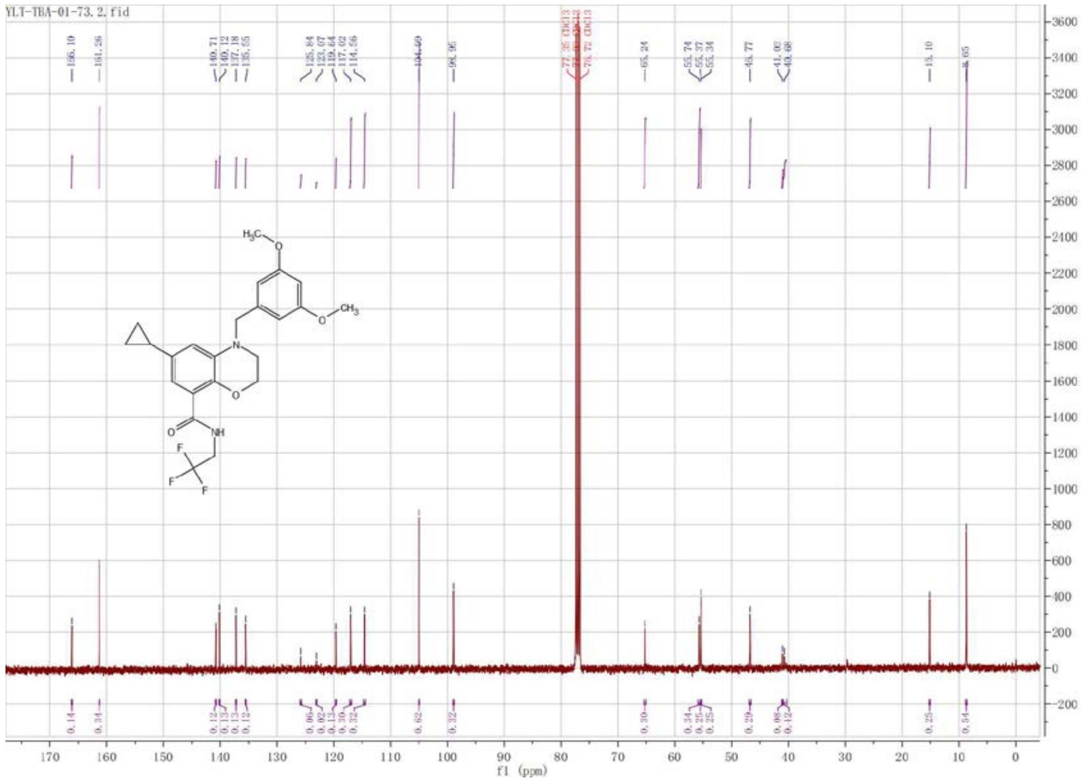

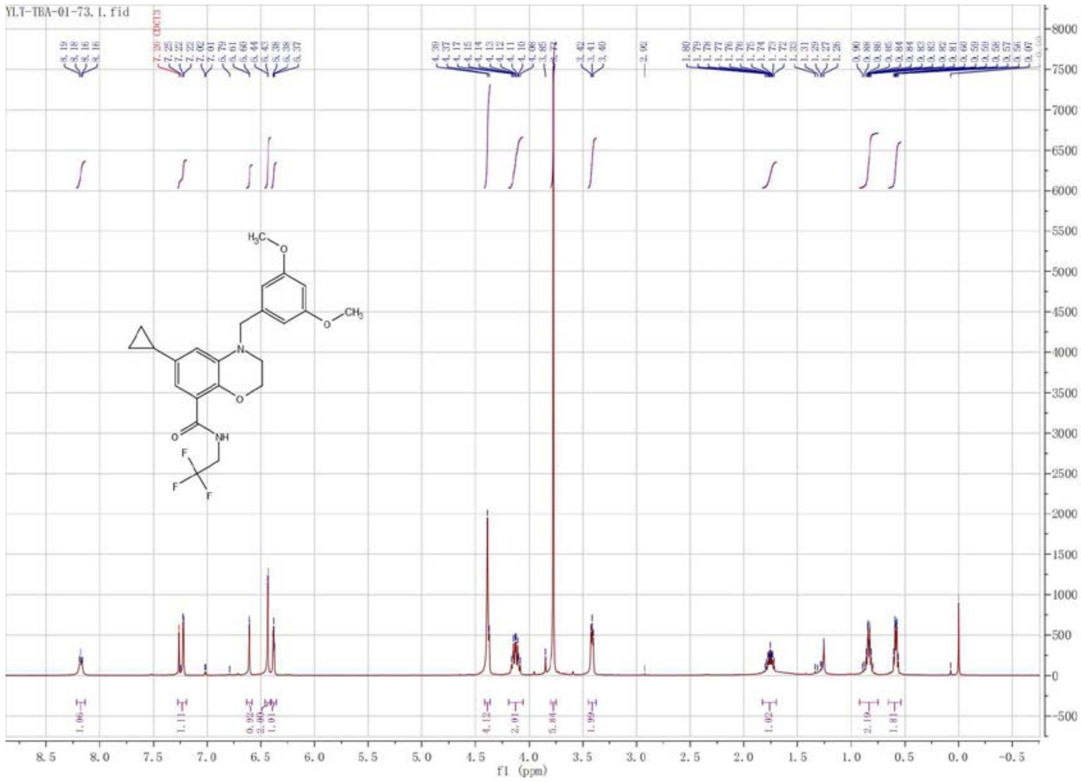

Compound **B32**

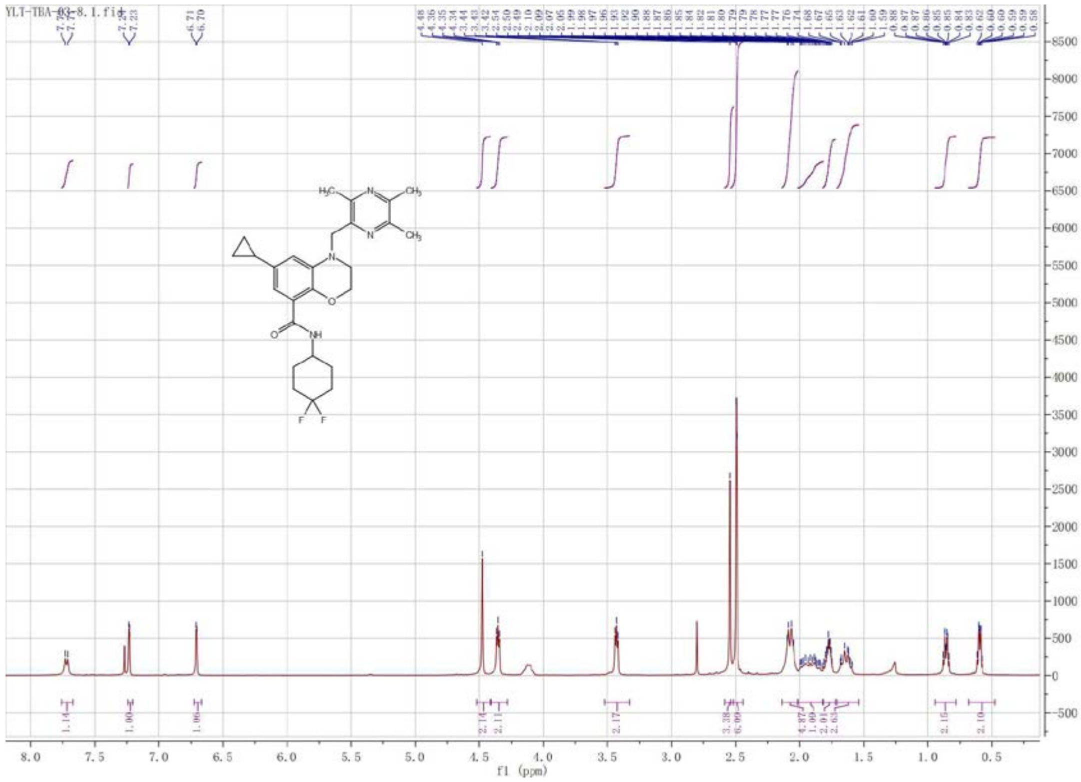

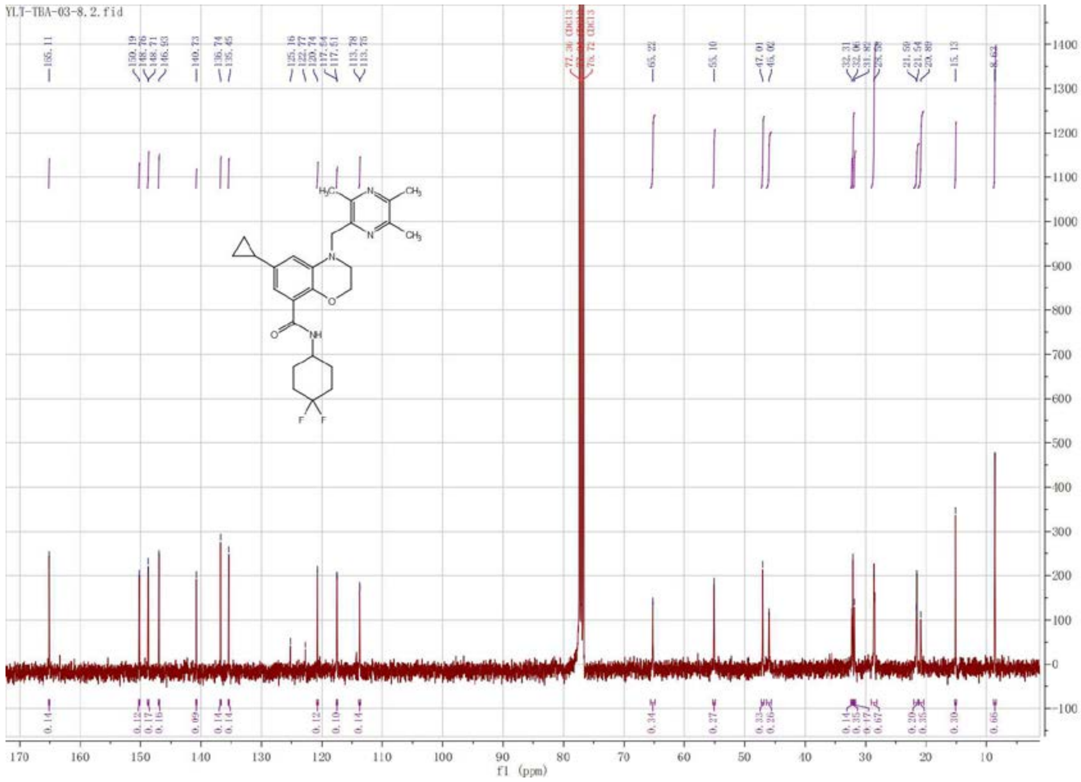

Compound **B31**

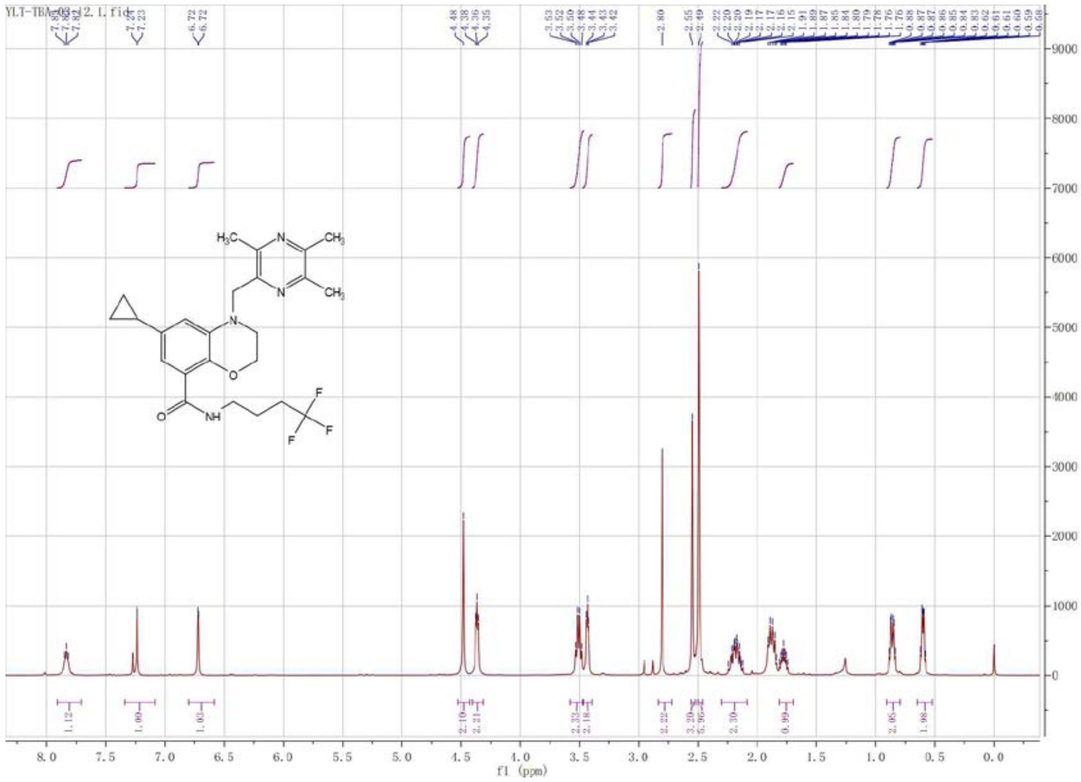

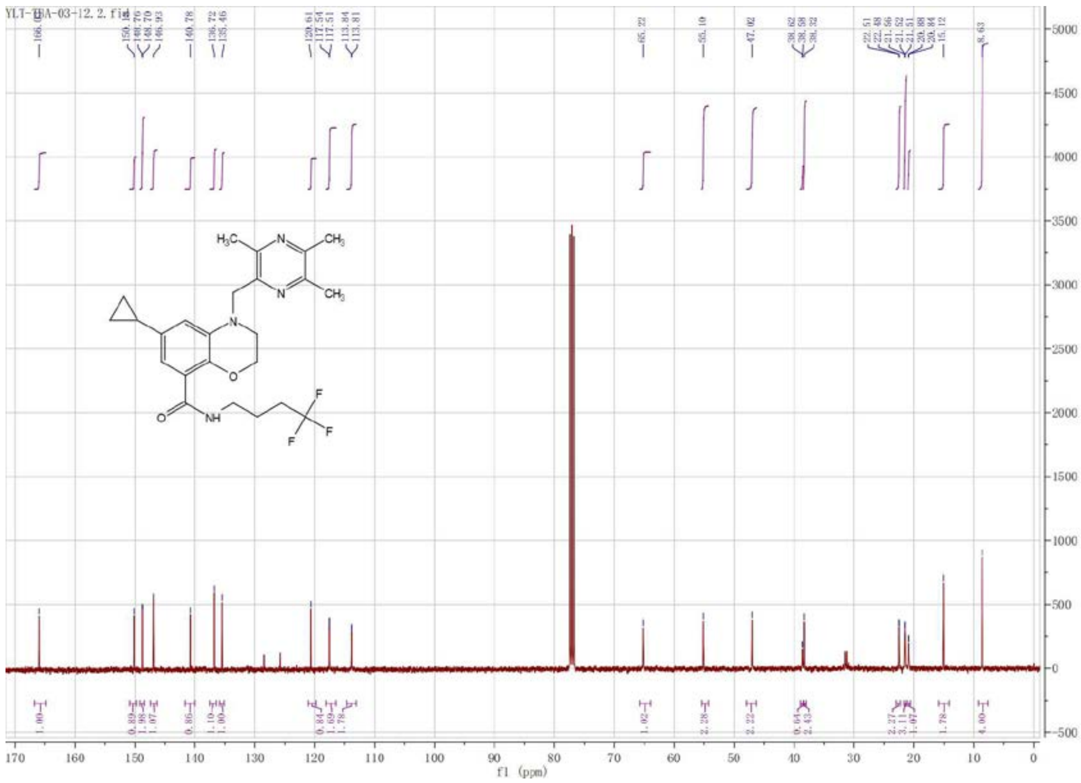

(5) HPLC for purity determination of representative compounds

Compound **B11**

Sample Name:	11	Injection Volume:	20.0
Vial Number:	RA5	Channel:	UV_VIS_2
Sample Type:	unknown	Wavelength:	240.0
Control Program:	80%MeOH-20%H2O	Bandwidth:	4
Quantif. Method:		Dilution Factor:	1.0000
Recording Time:	2021/11/22 14:03	Sample Weight:	1.0000
Run Time (min):	15.00	Sample Amount:	1.0000

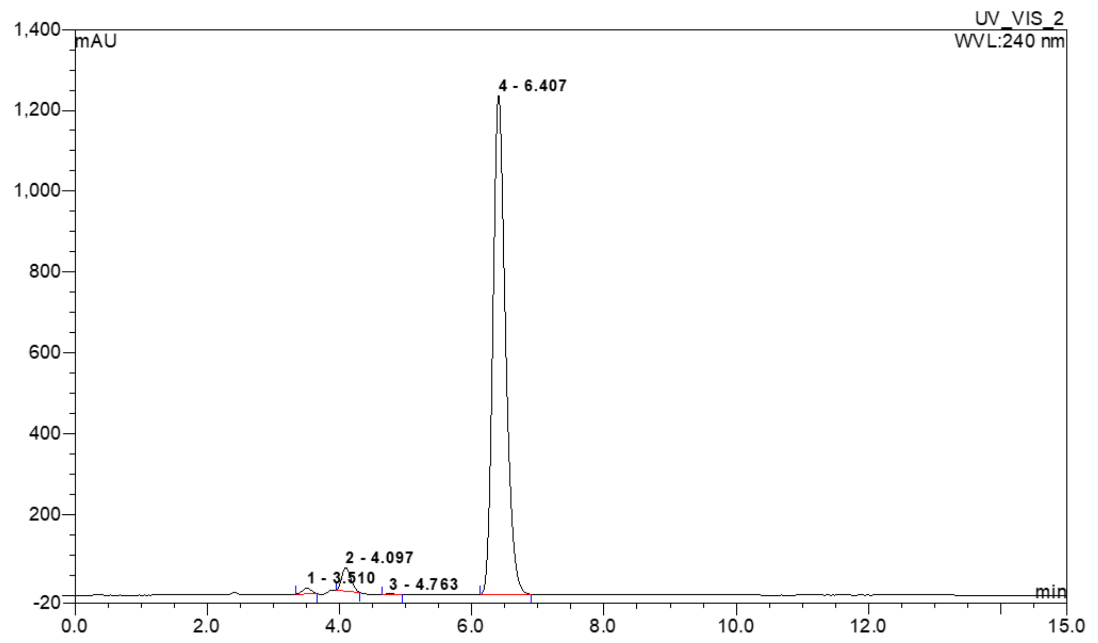

No.	Ret.Time min	Peak Name	Height mAU	Area mAU*min	Rel.Area %	Amount	Type
1	3.51	n.a.	15.414	2.279	0.80	n.a.	BMB*
2	4.10	n.a.	58.326	9.185	3.23	n.a.	BMB*
3	4.76	n.a.	1.848	0.278	0.10	n.a.	BMB*
4	6.41	n.a.	1234.965	272.598	95.87	n.a.	BMB*
Total:			1310.553	284.339	100.00	0.000	

Compound **B15**

Sample Name:	**15**	*Injection Volume:*	**20.0**
Vial Number:	**RB7**	*Channel:*	**UV_VIS_2**
Sample Type:	**unknown**	*Wavelength:*	**240.0**
Control Program:	**80%MeOH-20%H2O**	*Bandwidth:*	**4**
Quantif. Method:		*Dilution Factor:*	**1.0000**
Recording Time:	**2021/11/22 16:48**	*Sample Weight:*	**1.0000**
Run Time (min):	**15.00**	*Sample Amount:*	**1.0000**

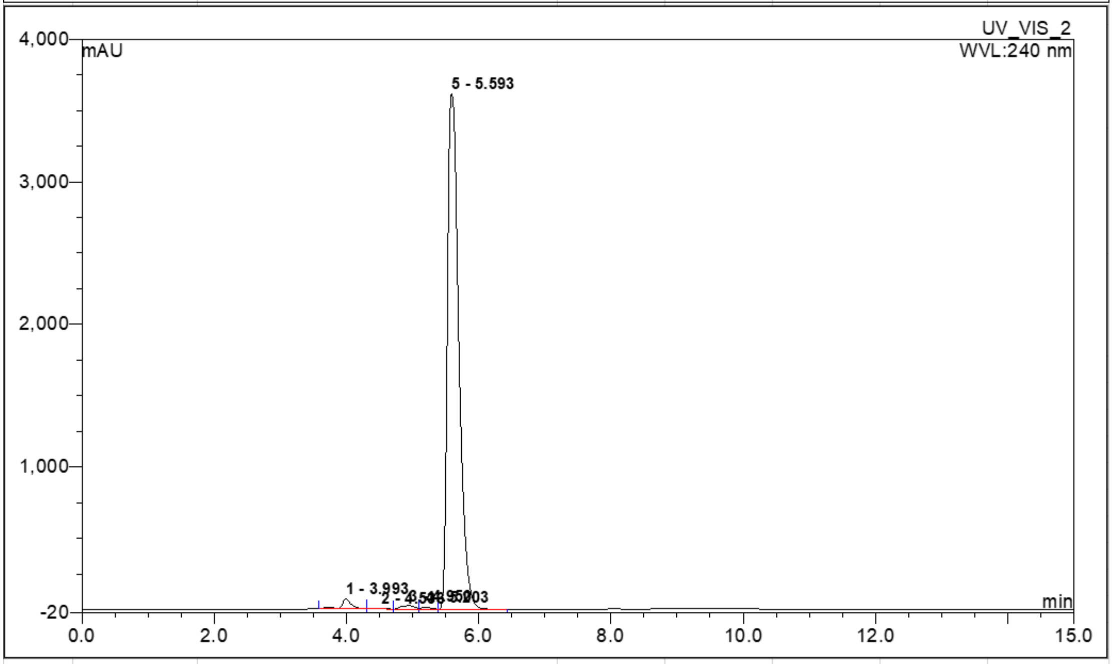

No.	Ret.Time<br>min	Peak Name	Height<br>mAU	Area<br>mAU*min	Rel.Area<br>%	Amount	Type
1	3.99	n.a.	71.975	12.298	1.66	n.a.	BMb*
2	4.53	n.a.	4.857	0.712	0.10	n.a.	bMB
3	4.95	n.a.	25.215	5.837	0.79	n.a.	BM
4	5.20	n.a.	14.532	2.161	0.29	n.a.	MB
5	5.59	n.a.	3612.992	721.566	97.17	n.a.	BMB
**Total:**			3729.571	742.573	100.00	0.000	

Compound **B18**

Sample Name:	18	*Injection Volume:*	20.0
Vial Number:	RB3	*Channel:*	UV_VIS_2
Sample Type:	unknown	*Wavelength:*	240.0
Control Program:	80%MeOH-20%H2O	*Bandwidth:*	4
Quantif. Method:		*Dilution Factor:*	1.0000
Recording Time:	2021/11/22 15:42	*Sample Weight:*	1.0000
Run Time (min):	15.00	*Sample Amount:*	1.0000

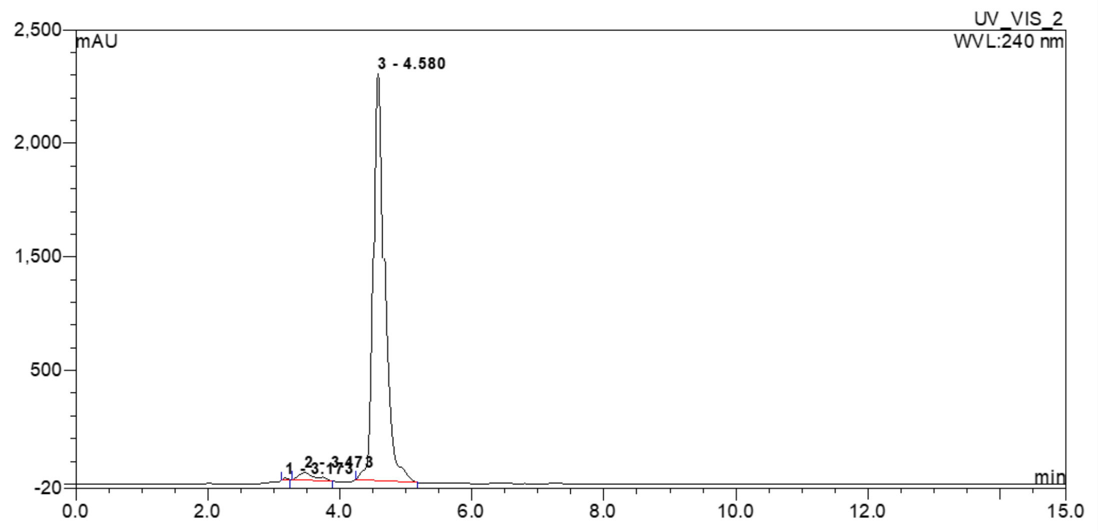

No.	Ret.Time min	Peak Name	Height mAU	Area mAU*min	Rel.Area %	Amount	Type
1	3.17	n.a.	8.722	0.629	0.14	n.a.	BMB*
2	3.47	n.a.	33.236	9.599	2.14	n.a.	BMB*
3	4.58	n.a.	2290.911	438.256	97.72	n.a.	BMB*
**Total:**			2332.869	448.484	100.00	0.000	

Compound **B30**

Sample Name:	**30**	*Injection Volume:*	**20.0**
Vial Number:	**RA5**	*Channel:*	**UV_VIS_2**
Sample Type:	**unknown**	*Wavelength:*	**240.0**
Control Program:	**80%MeOH-20%H2O**	*Bandwidth:*	**4**
Quantif. Method:		*Dilution Factor:*	**1.0000**
Recording Time:	**2021/11/22 17:37**	*Sample Weight:*	**1.0000**
Run Time (min):	**15.00**	*Sample Amount:*	**1.0000**

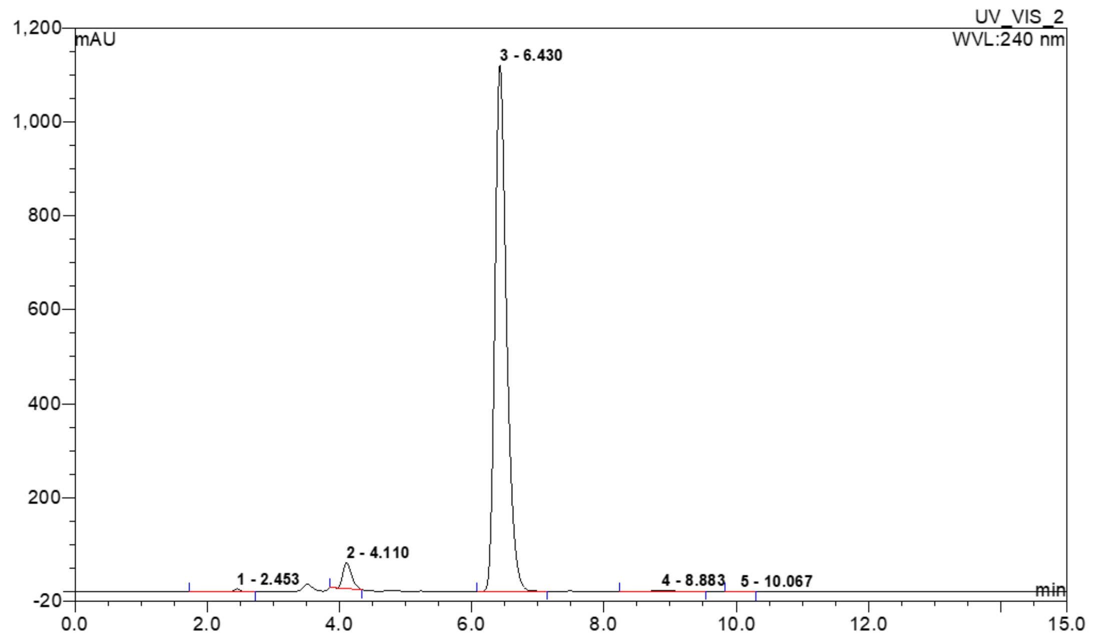

No.	Ret.Time min	Peak Name	Height mAU	Area mAU*min	Rel.Area %	Amount	Type
1	2.45	n.a.	5.773	0.707	0.30	n.a.	BMB*
2	4.11	n.a.	55.946	8.505	3.55	n.a.	BMB*
3	6.43	n.a.	1120.095	228.951	95.64	n.a.	BMB*
4	8.88	n.a.	2.713	1.127	0.47	n.a.	BMB*
5	10.07	n.a.	0.336	0.090	0.04	n.a.	BMB*
**Total:**			1184.863	239.381	100.00	0.000	
